# Taxonomic-Level Protein Quantification in Metaproteomics Using a Biomass-Constrained Expectation–Maximization Approach

**DOI:** 10.1021/jasms.5c00332

**Published:** 2026-01-15

**Authors:** Gelio Alves, Mehdi B. Hamaneh, Aleksey Y. Ogurtsov, Yi-Kuo Yu

**Affiliations:** Division of Intramural Research, 10952 National Library of Medicine, National Institutes of Health, Bethesda, Maryland 20894, United States

## Abstract

Microbiome communities are found across diverse environments and play critical roles in both ecosystem function and human health. Mass-spectrometry-based metaproteomics provides a powerful means for directly identifying and quantifying microbial proteins. However, its application is hindered by the shared peptide problem, where peptides map to multiple proteins across taxa, complicating taxon–protein quantification. To address this challenge, we extend a previously published modified expectation–maximization algorithm that incorporates taxonomic biomass constraints into the Microorganism Classification and Identification (MiCId) workflow. This enhanced expectation–maximization algorithm is used to quantify taxon–protein pairs derived from clusters of identified taxon–protein pairs, thereby enabling more accurate quantification and representation of taxonomic-level proteomes. The performance of the approach is evaluated using synthetic datasets consisting of simple mixtures with known relative species abundances, a more complex 24-species synthetic dataset, and a clinical human stool microbiome dataset. It is shown that, in simple synthetic datasets, fold changes computed for species–protein pairs closely match the expected values and are consistent with those obtained from MaxQuant. Using the 24-species synthetic dataset, we show that the algorithm accurately redistributes peptide extracted ion count among taxon–protein pairs that share peptides. Finally, analyzing the clinical stool microbiome dataset, we demonstrate that MiCId’s results are accurate and consistent with previously reported findings. These results demonstrate the robustness of MiCId’s algorithm for quantifying taxon–protein pairs in complex microbial communities. By resolving the shared peptide problem, the method enables accurate representation of taxonomic-level proteomes, thereby advancing the application of metaproteomics in microbiome research.

## Graphical abstract

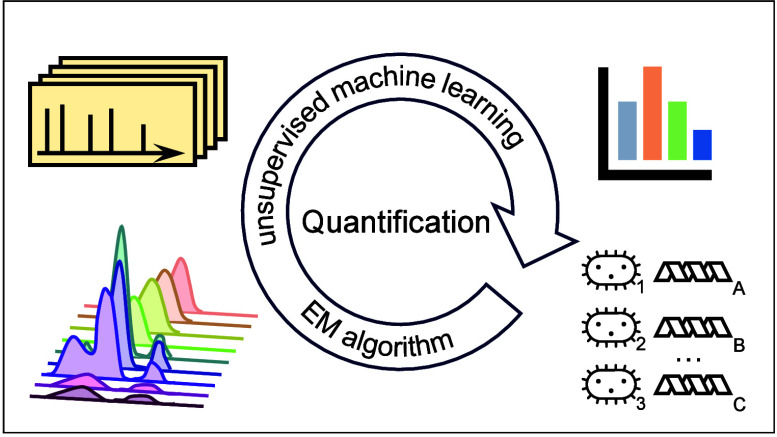

## Introduction

Microbiome communities exist almost everywhere on earth. They are present in a diversity of environments and interact with living and nonliving systems. Microbiome communities can be found, for example, in animals, plants, soil, lakes, rivers, oceans, homes, workplaces, and hospitals. Microbiomes are essential to the operation of nearly all ecosystems, facilitating essential functions such as nutrient cycling, decomposition, climate regulation, and the well-being of living organisms.^1–7^

The human microbiome is made of trillions of microorganisms.^8^ These microorganisms play a significant role in human health and disease. For instance, imbalance in the microbial communities of the human intestines are correlated with conditions such as inflammatory bowel diseases, type 2 diabetes, obesity, atherosclerosis, and neurodevelopmental disorders.^1,9^ Furthermore, microorganisms in the human gut have an assemblage of enzymes that can alter drugs, potentially activating, deactivating, or even toxifying them.^10^ As another example, microorganisms in the human oral microbiome are involved in oral diseases such as dental caries, periodontal disease, and oral cancer.^11,12^

Given the significant roles that microbiome communities play in human health, disease, and in our society, there is a need for high-throughput methods capable of interrogating these complex systems. While techniques such as metagenomics and metatranscriptomics provide valuable insights into the composition, genetic potential, and gene expression profiles of microbial communities, it is high-throughput, mass-spectrometry (MS)-based metaproteomics that allows for the direct identification and quantification of the proteins produced from these genes.^13–17^ Moreover, metaproteomics enables the estimation of microbial biomass distribution and characterization of protein states due to post-translational modifications (PTMs), which can cause change in protein structure and function.^18–21^

To gain a detailed understanding of microbiome communities through metaproteomics, data-analysis workflows must be able to report, for each identified taxon, its corresponding expressed proteins, i.e., taxon–protein pairs, thereby revealing each taxon’s contribution to the community. However, reporting taxon-protein pairs across different taxonomic levels is a nontrivial task due to the *shared peptide problem*. The shared peptide problem in mass-spectrometry-based proteomics was first recognized in studies of samples containing a single eukaryotic organism.^22^ This issue complicates protein inference, particularly for highly homologous proteins, but it can also arise for nonhomologous proteins that nevertheless share a small number of tryptic peptides. As a result, proteins are typically grouped based on sequence similarity or the set of nonredundant, confidently identified peptides during protein inference.^23^ The protein with the highest number of such peptides is often selected as the representative of the group, a practice that contributes to the ongoing debate surrounding protein inference in mass-spectrometry-based proteomics.

In metaproteomics, this problem is even more pronounced because peptides may be shared not only among homologous proteins within a single taxon but also across proteins from multiple taxa represented in the microbial protein database.^22–26^ Similar to conventional proteomics workflows, proteins that are homologous or share a substantial number of peptides are typically clustered together during protein inference. This clustering further complicates the accurate reporting of taxon–protein pairs across taxonomic levels, as shared peptides obscure the ability to uniquely assign proteins to specific taxa.

To address the shared peptide problem, most MS-based metaproteomics workflows employ the Lowest Common Ancestor (LCA) algorithm to assign taxonomic information to identified peptides, proteins, and biological functions.^27–33^ While useful, the LCA algorithm does not guarantee taxonomic or functional assignments across the full lineage of each identified microorganism. Additionally, LCA suffers from large database issues. In particular, the accuracy of Unipept,^29^ an application that uses LCA for metaproteomics analyses, varies depending on the size of the search database.^34^ To overcome some of these challenges, in the context of functional analysis, recent studies have introduced three alternative approaches aimed at improving the accuracy of taxon and taxon–function pair assignments in metaproteomics.

The first approach, implemented in the metaQuantome workflow,^33,35^ can be summarized as follows: peptides and their associated features are annotated with the LCA method in Unipept to the most specific taxonomic level supported by the data. These annotations are then propagated upward through all ancestral taxonomic levels, such that the abundance of a taxon or functional term at a given level is calculated as the sum of abundances from peptides specific to that level, along with contributions from descendant peptides. This approach enables quantification of taxonomic and functional profiles across hierarchical levels but relies exclusively on taxon-specific peptides. Consequently, peptide features can be merged upward but not partitioned downward among multiple taxa, reflecting the method’s intentional avoidance of fractional or probabilistic assignment of shared peptides. In other words, the technique can aggregate peptide contributions but cannot split them, a limitation that restricts proper handling of shared peptides across related taxa and functional terms.

The Second approach, implemented in the MetaX workflow,^36^ adopts a peptide-centric strategy for both taxonomic and functional assignments. For each identified peptide, a taxon proportion is calculated as the ratio of the occurrences of the most frequent taxon among all associated taxon–protein pairs containing the peptide to the total number of such pairs. This proportion is evaluated at each taxonomic rank, beginning at the genome level and progressing up to the domain level, and the peptide is assigned to the most specific rank where the computed proportion meets or exceeds a user-defined threshold. Functional annotation follows a similar procedure: the most frequent function among all associated protein-function pairs is selected for each peptide, with its proportion defined as the ratio of the most frequent function to the total number of protein-function pairs. Because this approach employs a locally optimal choice, peptides are not assigned to all taxa or protein-function pairs sharing them, but only to the most representative ones.

The third approach, implemented in the MiCId workflow,^37^ employs a modified expectation–maximization (EM) algorithm to distribute the extracted ion count (EIC) of identified peptides among all taxa and taxon-function pairs that share those peptides across taxonomic levels. In this framework, peptides are mapped to taxon-function pairs through the proteins of the corresponding taxon-protein pairs. The procedure is applied across multiple taxonomic ranks, from species to phylum, with an additional root level representing the highest taxonomic category. Using the EIC from identified peptides, the modified EM algorithm first estimates a probability for each taxon, inferring taxonomic identifications from the peptide set. These computed probabilities are then used as constraints to determine the joint probability of observing a biological function within a given taxon.

In this study, we extend the previously published modified EM algorithm^37^ which incorporates taxonomic biomass constraints to compute the abundances of taxon–protein pairs across multiple taxonomic levels. This approach leverages evidence from all nonredundant identified peptides, rather than relying solely on unique peptides. Using the EIC from confidently identified peptides, the modified EM algorithm first calculates a probability for each identified taxon, where taxonomic identifications are inferred from the set of confidently identified peptides. Next, utilizing these computed probabilities as constrains, the EM algorithm determines the joint probability of observing a protein from a given taxon. An outline of the workflow is provided in Figure 1 and details of the steps involved in the proposed approach, as implemented in the MiCId workflow, are provided in the Methods section. Our objective is to demonstrate that this EM algorithm can be applied to quantify taxon–protein pairs decomposed from clusters of identified taxon–protein pairs, thereby enabling accurate quantification and representation of taxonomic-level proteomes, an essential capability for advancing microbiome research through metaproteomics.

**Figure 1:**
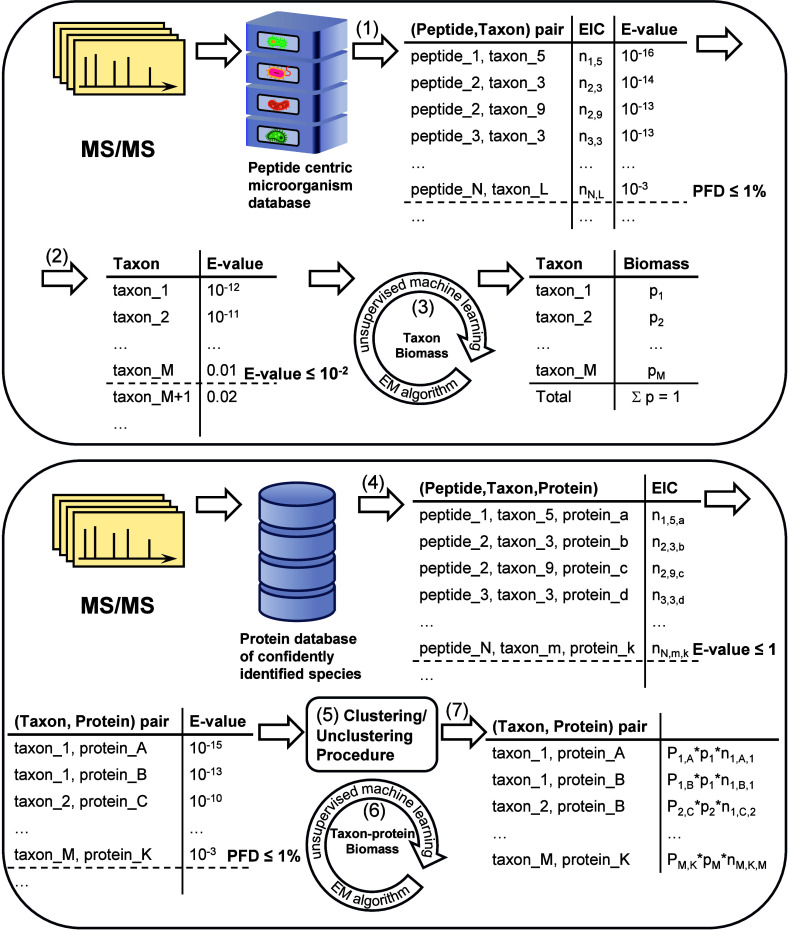
Workflow of MiCId. The seven major steps of the microorganism identification and protein–taxon pair quantification workflow, labeled (1)–(7), are depicted. The numbered labels in parentheses correspond to the step numbers described in the main text. In this workflow, EIC and PFD refer to extracted ion count and proportion of false discoveries, respectively. The EIC associated with peptide π_*i*_ and taxon *t*_α_ is represented by *n*_*i*,α_ in step (1). When the EIC is instead associated with protein *k*, as in step (4), it is represented as *n*_*i*,α,*k*_. Finally, the portion of the EIC of peptide π_*i*_ that is distributed to protein *k* from taxon *t*_α_ is expressed as *P*_α,k_*p_α_*n_*i*,α,*k*_, as shown in step (7). Here, *p*_α_ and *P*_α,*k*_ are probabilities estimated using the proposed EM algorithm in steps (3) and (6), respectively.

To illustrate the problem we aim to address, consider a simple case involving two closely related bacterial species, A and B. When analyzing samples containing each species individually using MS-based proteomics, we identify n_*A*_ and n_*B*_ protein clusters for species A and B, respectively. However, due to the shared-peptide problem, analysis of a mixed sample containing both species may yield only n_*C*_ protein clusters, where n_*C*_ is smaller than the expected n_*A*_ + n_*B*_ protein clusters. The proposed EM approach overcomes this limitation by decomposing clusters of identified taxon–protein pairs into their corresponding individual taxon–protein pairs, while simultaneously providing quantitative estimates across multiple taxonomic levels. This extended EM algorithm has been fully implemented within the MiCId workflow.

MiCId, which stands for Microorganism Classification and Identification, is a self-contained MS-based metaproteomics workflow that was originally developed for the rapid identification of samples composed of single microorganisms.^38^ It was later expanded to support the identification of mixed microbial samples.^39^ More recently, MiCId has been augmented with capabilities for detecting antibiotic-resistance proteins,^40^ estimating microbial biomass,^41^ and performing functional analysis.^37^ At its core, MiCId incorporates a robust statistical significance framework that assigns *E*-values to identified peptides, proteins, and microorganisms.^42–44^ When accurately computed, these *E*-values provide a principled means of controlling the number of false-positive peptide identifications,^45,46^ as well as false-positive protein and microorganism identifications. Rigorous control of false positives, particularly at the peptide level, is a key requirement for the proper functioning of the proposed EM algorithm.

To assess the performance of the proposed EM algorithm implemented in MiCId, we analyzed both synthetic and clinical datasets. Using three simple synthetic datasets with known relative species abundances,^47–49^ we show that the fold changes computed for species–protein pairs closely match the expected values and are consistent with those obtained using MaxQuant. Using a more complex 24-species synthetic dataset designed to evaluate protein assignment in challenging mixtures,^50^ we demonstrate that the algorithm can accurately distribute peptide EICs among taxon–protein pairs that share peptides. Finally, we evaluate the proposed EM algorithm using a clinical human stool microbiome dataset^16^ and show that (1) the log fold changes computed for protein–taxon pairs across technical replicates are, as expected, close to zero, (2) the estimated relative biomass abundances and protein abundances are consistent with those reported in the original study, and (3) Gene Ontology (GO)^51^ term enrichment analysis of the identified host (human) proteins reveals enrichment in inflammatory bowel disease (IBD) samples but not in normal samples, with several of these GO terms previously implicated in IBD through genomic studies. Together, these results highlight the robustness of the proposed EM algorithm for quantifying taxon–protein pairs in complex microbiome samples where shared peptides are common.

## Methods

### Overview of MiCId Taxon-Specific Protein Quantification

To identify and quantify taxon-protein pairs at different taxonomic levels, MiCId takes several steps: (1) taxonomic-level peptide identification, (2) taxonomic-level microorganism identification, (3) taxonomic-level relative biomass calculation, (4) taxon-protein pair identification, (5) taxon-protein pair clustering/unclustering, (6) splitting peptide EIC between different taxon-protein pairs, and (7) taxon-protein pair quantification. It is important to note that all steps, except for peptide/protein identification, are repeated for each taxonomic level. These steps are depicted in Figure 1 and are described in detail in the following subsections.

### Taxonomic-Level Peptide Identification

In the first step of the process, MiCId uses the raw MS/MS files and a peptide-centric database for peptide identification.^38,39^ Specifically, given the spectra obtained from the raw files, a search is conducted to identify matching peptides in the database. Each identified peptide is then assigned an *E*-value.^42^

### Taxonomic-Level Microorganism Identification

Next, peptides with *E*-values below a cutoff that controls the proportion of false discoveries (PFD) at 1% are selected for microorganism identification. These peptides are then used to compute combined *E*-values for each taxon, and the taxa with *E*-values ≤ 0.01 are considered confidently identified. Further details on microorganism identification and *E*-value assignment are provided elsewhere.^38^

### Taxonomic-Level Relative Biomass Calculation

At this step, MiCId employs a modified version of the EM algorithm to estimate the biomass of all confidently identified taxa. This algorithm has been described in detail in Text S1. A brief description of the algorithms is also provided in the “Splitting Peptide EIC between Different Taxon-Protein Pairs” subsection, which also explains the second round of using the EM algorithm for estimating taxon-protein pair abundances at each taxonomic level. This second round uses the biomass values, which are constrained to sum to 1, obtained in this step.

### Taxon-Protein Pair Identification

Since microorganisms in the sample have been already identified confidently, to have better statistical power, a reduced database containing the protein sequences from confidently identified species is constructed. A second peptide search against this reduced database is conducted and the identified peptides are assigned *E*-values.^38^ The confidently identified peptides (with *E*-value ≤ 1) are then used to identify taxon-protein pairs and calculate their corresponding *E*-values and PFD. The approach taken for protein identification and statistical significance assignment is detailed in a previous publication.^43^ At this stage, the confidently identified peptides are also quantified using their EIC from the raw MS/MS files.

### Taxon-Protein Pair Clustering/Unclustering

Next, the identified taxon-protein pairs are clustered based on the number of confidently identified nonredundant peptides shared between them. The clustering algorithm is described in detail elsewhere,^37^ and in the interest of brevity is not repeated here. The clustering is followed by an unclustering procedure as follows. First, taxon-protein clusters containing at least one taxon-protein with PFD ≤ 1% are selected. Next, within each cluster, the taxon-protein pairs are grouped based on the taxon they belong to. If a protein is shared between multiple taxa, it will appear in all corresponding groups. From each group in each cluster, the protein with the lowest *E*-value is then selected. If some proteins in a group within a cluster are tied (in terms of *E*-value), no protein is selected from the group. Finally, taxon-protein pairs with PFD > 1% are filtered out. The end result of this procedure is a list of taxon-protein pairs that is saved for subsequent analyses.

### Splitting Peptide EIC between Different Taxon-Protein Pairs

Since each confidently identified peptide may be shared among multiple taxon–protein pairs, its EIC must be appropriately partitioned across these pairs. In MiCId, this distribution is achieved through an EM algorithm. The EM algorithm is a powerful statistical approach that maximizes the likelihood of observed data by iteratively updating model parameters and estimating the expectation of hidden or unobserved variables. In this context, the model parameters correspond to the probabilities that confidently identified peptides originate from proteins belonging to confidently identified taxa, the hidden variables represent the true contributions of each peptide’s EIC to the proteins and taxa from which the peptides arose, while the observed data represent the EIC of confidently identified peptides.

Because the distributions of these hidden variables depend on the model parameters, direct likelihood optimization is difficult. The EM algorithm circumvents this challenge by dividing the optimization into three steps: initialization, expectation, and maximization. In the initialization step, the model parameters are assigned starting values. For example, for taxonomic biomass calculations the model parameters are initialized to 1/*M*, where *M* is the total number of confidently identified taxa. The expectation step then uses these parameters to estimate the expected contributions of the hidden variables. In the maximization step, the parameters are updated to maximize the likelihood using these expected values in place of the unknown variables. The expectation and maximization steps are repeated iteratively until convergence. For the problem at hand, the modified EM algorithm is applied in two rounds at each taxonomic level, enabling consistent estimation and assignment of protein–taxon pairs across multiple taxonomic resolutions.

In the first round of the modified EM algorithm it estimates probabilities (see the “Taxonomic-Level Relative Biomass Calculation” subsection), denoted as *p*(*t*_α_), for each identified taxon *t*_α_ based on the MS^1^ EIC of confidently identified peptides. These taxon-level probabilities are constrained such that their total across all identified taxa equals 1. Importantly, these probability estimates represent the relative biomass contributions of the taxa in the sample. In particular, *p*(*t*_α_) corresponds to the relative biomass of taxon *t*_α_.

In the second round of the modified EM algorithm, the previously estimated *p*(*t*_α_)­s values are used as constraints to compute the joint probability *p*(*k*|*t*_α_)*p*(*t*_α_) for each identified protein-taxon pair *k*, based on the MS^1^ EIC of confidently identified peptides. This joint probability represents the likelihood of observing protein *k* as originating from taxon *t*_α_. In this framework, *p*(*k*|*t*_α_)*p*(*t*_α_) reflects the portion of taxon *t*_α_’s biomass attributed to protein *k*. Due to the biomass normalization constraint, summing *p*(*k*|*t*_α_)*p*(*t*_α_) over all protein–taxon pairs linked to taxon *t*_α_ yields the estimated biomass fraction of taxon *t*_α_. This ensures that the probabilities *p*(*k*|*t*_α_)*p*(*t*_α_) estimated for the proteins of *t*_α_ collectively sum to the relative biomass of taxon *t*_α_.

In the first round of the EM algorithm, the probabilities *p*(*t*_α_)­s are initialized to 1/*M*, where *M* is the total number of confidently identified taxa. The expectation and maximization steps are repeated until numerical convergence is achieved for the probabilities *p*(*t*_α_)­s. Once the taxa probabilities are determined, the algorithm proceeds to the second round of the EM step. Here, the *p*(*k*|*t*_α_)­s values are initialized to 1/*K*, where *K* is the total number of proteins. A detailed derivation of the EM formulation is provided in Text S1, and a high-level schematic of the complete workflow is shown in Figure 1.

### Taxon-Protein Pair Quantification

At the final step, the peptide EIC for each taxon-protein pair is used to calculate the relative abundance of the pair. Although MiCId quantifies taxon-protein pairs, i.e., computes relative taxon-protein intensities from the peptide EIC, the computed values across different samples may not be comparable. In other words, between-sample normalization should be performed to get the final relative abundances. Currently, such a normalization method is not implemented in MiCId. Thus, we used the normalization approach implemented in directLFQ (see the “Protein quantification using directLFQ” section)^52^ for normalization. As mentioned previously, except for peptide/protein identification, all steps in the MiCId workflow are repeated for all taxonomic levels. Hence, the final output of this multistep workflow is a list of intensities for all confidently identified taxon-protein pairs at different taxonomic levels.

### MS/MS Data

To test MiCId’s ability to identify/quantify taxon-protein pairs at different taxonomic levels, we used five publicly available MS/MS datasets comprising four synthetic (with known protein abundance ratios) and one natural datasets. These datasets, which were downloaded from ProteomeXchange (https://www.proteomexchange.org/),^53^ are briefly described below and are summarized in Table 1.

**Table 1:** Datasets Used for Testing MiCId

	ID	N. of files	N. of species	Description
Dataset 1	PXD028735	24	3	Synthetic mixture
Dataset 2	PXD007683	11	2	Synthetic mixture
Dataset 3	PXD006109	6	2	Synthetic mixture
Dataset 4	PXD005776, PXD005778	3, 24	24	Synthetic mixture, individual species
Dataset 5	PXD005619	45	Unknown	Clinical human stool microbiome

**Dataset 1:** Synthetic dataset including mixtures of Yeast, Human, and *E. coli*,^47^ with raw files obtained under different conditions, using both data-dependent acquisition (DDA) and data-independent acquisition (DIA), and employing various instruments. For this study only DDA data obtained using Oribtrap instruments and under conditions “A” and “B” were included. Samples under the two conditions have different (but known) quantities of Yeast and *E. coli*.

**Dataset 2:** Dataset containing three synthetic mixtures of known amounts of Yeast and Human biomass.^48^ For this study, we used only the label-free data.

**Dataset 3:** Synthetic dataset including two mixtures of known quantities of *E. coli* and Human biomass.^49^

**Dataset 4:** Synthetic dataset containing three raw files representing technical replicates of an equimolar mixture of 24 species, including *Bacillus cereus*, *Bacillus subtilis*, *Bacillus thuringiensis*, *Bordetella parapertussis*, *Cellulophaga lytica*, *Deinococcus deserti*, *Deinococcus geothermalis*, *Deinococcus proteolyticus*, *Kineococcus radiotolerans*, *Marivirga tractuosa*, *Oceanicola granulosus*, *Phaeobacter inhibens*, *Pseudomonas putida*, *Pseudopedobacter saltans*, *Rhizorhabdus wittichii*, *Roseobacter denitrificans*, *Roseovarius nubinhibens, Ruegeria pomeroyi*, *Sagittula stellata*, *Salmonella bongori*, *Shigella flexneri, Staphylococcus carnosus*, *Sulfitobacter indolifex*, and *Vibrio harveyi*.^50^ In addition to the three files containing data from the mixture, 24 raw files each corresponding to one of the species were also used for comparison.

**Dataset 5:** Human gut microbiome dataset comprising stool samples from four children. Two samples are from children with ulcerative colitis (HM604, HM621), one sample from a child with Crohn’s disease in remission (HM541), and one control sample without inflammatory bowel disease (IBD) (HM609).^16^ Counting technical replicates, this dataset has 9 samples, and for each sample two-dimensional liquid chromatography coupled with tandem mass spectrometry was conducted yielding a total of 45 raw data files.

### Running MiCId

For the synthetic datasets we generated peptide-centric databases for each dataset containing the reference proteome of the species presented in the samples. For the clinical human stool microbiome dataset the constructed peptide-centric database included 37,969 organisms. The protein sequences for this database were obtained via the National Center for Biotechnology Information (NCBI) BioSample database.^54^ In each case, we used the approach described in ref 38 to construct the peptide-centric database. The microorganisms included in these peptide-centric databases are listed in Supplementary Table S1.

When searching these databases using MiCId,^39^ the following parameters were applied. The digestion rules for trypsin and Lys-C were assumed, and up to two missed cleavage sites per peptide were allowed. Carbamidomethylation of cysteine was set as a fixed modification. The precursor and product ion mass error tolerance were extracted from the raw files for each dataset.

### Protein Intensity Normalization Using DirectLFQ

To compute the normalized protein intensities from quantified peptides, we employed directLFQ^52^ version 0.3.1. For each protein, directLFQ takes as input a list of identified ions (peptides/charges) and their calculated intensities in all samples and computes the normalized protein intensities across all samples. When running directLFQ, in all cases the default parameters were used.

### Running MaxQuant

We also compared the performance of MiCId with that of MaxQuant in regard to protein quantification for Datasets 1 (Table 1). MaxQuant was run using the same fasta and raw files used for running MiCId, and each raw file was considered as a separate experiment. All cysteines were considered to be carbamidomethylated (fixed modification). No other modification was included. The rest of the parameters were set at their default values. Since directLFQ has been reported to outperform^52^ the MaxLFQ algorithm implemented in MaxQuant, and to have a fair comparison with MiCId+directLFQ, instead of using protein abundances reported by MaxQuant we used the “evidence.txt” output file of MaxQuant as input to directLFQ for protein quantification. The performance of MiCId+directLFQ was then compared with that of MaxQuant+directLFQ.

## Results and Discussion

In this section the results of the assessment of MiCId using the 5 testing datasets are provided. First, we evaluate MiCId’s protein quantification results using simple synthetic cases (Dataset 1, 2, and 3), where proteins from different species are not likely to cluster together. Next, we assess quantification results for the synthetic case where a significant number of peptides are shared between species and clusters containing multiple species are common (Dataset 4). We then show that, for the clinical human stool microbiome dataset (Dataset 5), MiCId produces results that are in agreement with those reported in the literature. Finally, limitations of MiCId are briefly discussed.

### Assessment of Protein Quantification in Simple Cases

The synthetic datasets containing data from only a few species with known abundance ratios, namely Datasets 1,2, and 3 (Table 1), were used to evaluate the performance of MiCId+directLFQ in terms of protein quantification. For Dataset 1 (a 3-species mixture), at the species level, the distributions of the log fold changes under two experimental conditions are shown in Figures 2 A, B, and C for *E. coli*, Human, and Yeast, respectively. The figure indicates that the median log fold changes are close to the expected values (the red lines), and that the distributions are rather tight around the medians. The figures do show some outlier points, but these distributions are similar to those obtained using MaxQuant+directLFQ (see Figure S1). These observations indicate good (comparable with MaxQuant+directLFQ) quantification performance by MiCId+directLFQ.

**Figure 2:**
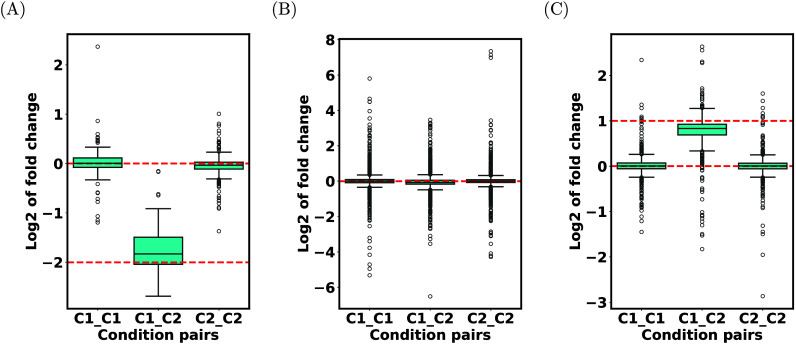
Species-level protein log fold changes for Dataset 1. Box plots of distributions of average log fold changes are shown for (A) *E. coli*, (B) human, and (C) yeast for Dataset 1 (PXD028735). The red lines show the expected values calculated based on the known abundances of the three species. C1 and C2 respectively represent the two conditions under which the data were collected. For the condition pair C*i*_C*j*, all pairs of distinct files consisting of one file from each condition were considered. In each case, the fold changes were computed for all considered file pairs and were averaged. For each pair of files, fold changes were computed only for proteins that were identified in both files.

For other taxonomic levels, we observed virtually identical distributions (Figures S2–S6). This is not surprising as the proteins of these species are not likely to cluster with each other. Also, good quantification performance was observed for Datasets 2 and 3. The log fold change distributions (at the species level only) for these two datasets are plotted in Figures S7 and S8.

### Assessment of Quantification of Taxon-Protein Pairs with Shared Peptides

To test the performance of MiCId in terms identification/quantification when proteins from multiple species cluster together and/or have shared peptides, we used Dataset 4 that contains three replicates of a mixture of 24 species (PXD005776) as well as 24 species-specific raw files each corresponding to one of the 24 species (PXD005728). Specifically, MiCId was run separately for each of the 24 single-species files, and separately for each of the three technical replicates containing the mixture of the 24 species. Of note, the experimental runs were much shorter for the mixture as opposed to species-specific. (For the mixture the experiment was run for 70 min that is around 3 min per species, but for each individual species the time was 90 min.) This resulted in a lower number of identified proteins for the mixture. To demonstrate the shorter run times were indeed responsible for the lower number of identified proteins in the mixture, we also ran MiCId for the 24 species-specific files together as one experiment, effectively generating a fake mixture that is henceforth referred to as the combined dataset. Figures 3 A and B respectively show the distributions of the number of species associated with each species-protein cluster for the combined and the mixture (averaged over the three replicates) cases. The two figures show rather similar trends, indicating a decrease in cluster frequency as the number of species in the cluster increases. The figures also show that a substantial proportion of clusters contain proteins from multiple species (26% in the combined and 14% in the mixture datasets), underscoring the need to split taxon–protein pairs from the identified clusters or groups. Furthermore, Figure S9 indicates that, on average, more than 21% of the peptides in the three mixture replicates, and 14% in the combined dataset, are shared among multiple taxon-protein pairs. This finding highlights the utility of the employed EM algorithm with biomass constraints to appropriately distribute EIC of confidently identified peptides across the taxon–protein pairs sharing them.

**Figure 3:**
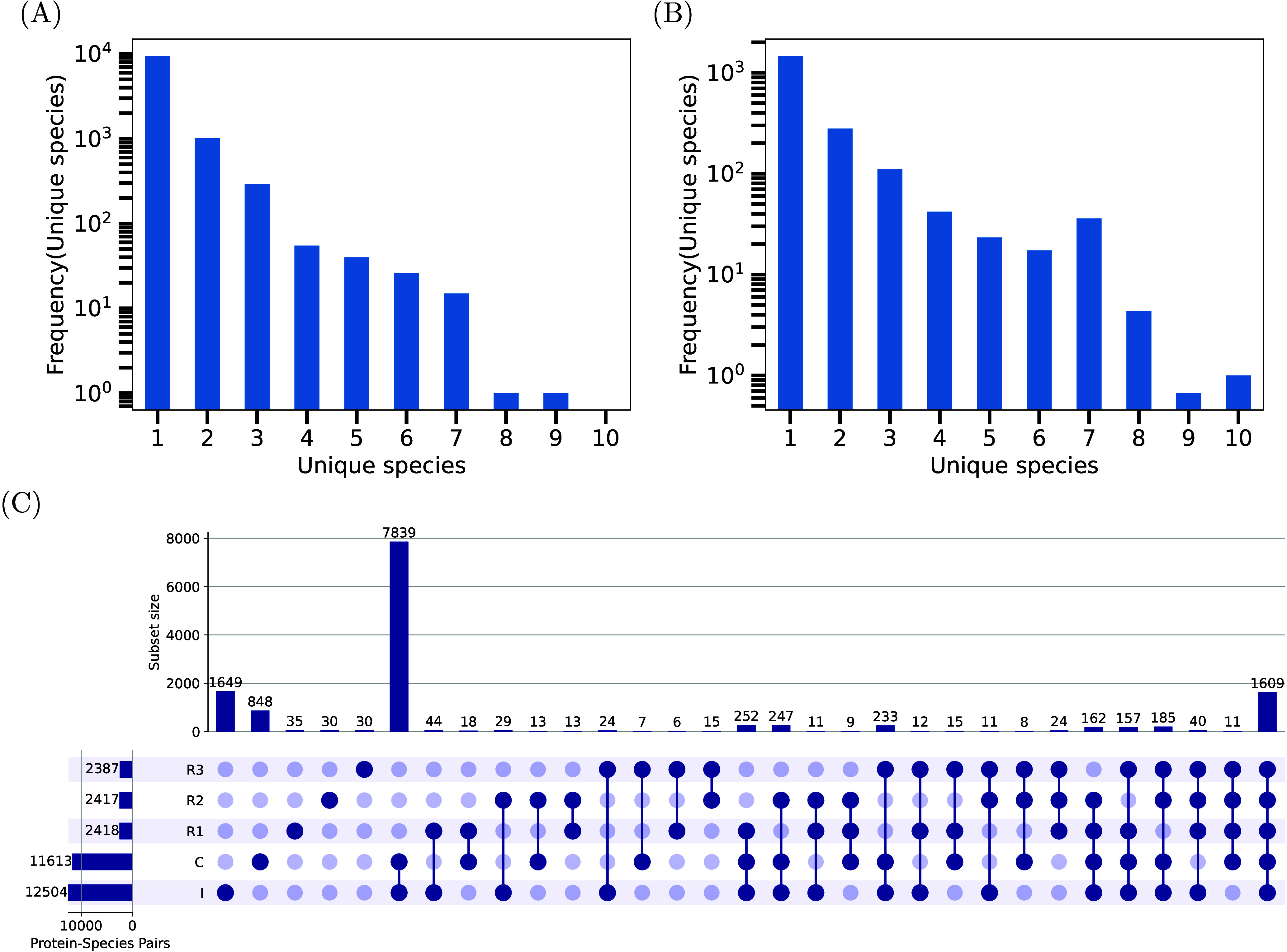
Assessing species-protein clustering and identification by MiCId. Panels A and B respectively show the distributions of the number of unique species in each species-protein cluster using the combined dataset and the three mixture files. In panel B the distribution has been averaged over the three files. Panel C contains a SetUp plot visualizing the overlap between the species-protein pairs identified by MiCId when replicate 1 (*R*1), replicate 2 (*R*2), replicate 3 (*R*3), the combined dataset (*C*), and the individual (*I*) raw files are used as input to MiCId. The horizontal bars (lower left) show the total numbers of identified pairs in each case. The vertical bars show the numbers of pairs exclusively identified in each case, or in any combination of the 5 cases. Species-protein pairs exclusively identified in two cases, for example, are the ones identified in both cases but missed in others. The (connected) black dot(s) below each vertical bar indicate(s) which cases were used to calculate the number of pairs represented by the bar.

The combined dataset can be used to test MiCId. Specifically, using the identification results obtained from the individual files as the gold standard, one can evaluate the ability of MiCId to recover these proteins from the combined dataset. The overlap between the sets of species-protein pairs identified using the combined dataset, denoted by *C*, the individual files, denoted by *I*, and each of the three replicate mixtures (*R*1, *R*2, and *R*3), are depicted in the SetUp plot shown in Figure 3 C. The SetUp plots at other taxonomic levels are shown in Figures S10–S14. To generate the set *I*, the union of the 24 sets of identified species-protein pairs obtained from the 24 individual files were used. Figure 3 C indicates that *I* and *C* have comparable sizes and that the two sets are highly concordant with 92% of the pairs in *C* also being present in *I*. As mentioned previously, because of the shorter experimental runs, *R*1, *R*2, and *R*3 sets are significantly smaller, but are almost subsets of *C* (more than 92% of the members of each of these three sets are also members of *C*). Also, these sets (*R*1, *R*2, and *R*3) are highly overlapped, with 77 to 80% of the members of each set being members of the other two as well. In this case the overlaps are not as high as the one observed between *I* and *C*, which is expected as these are separate experiments. Overall, our observations demonstrate that MiCId provides robust and accurate identification performance, correctly splitting taxon–protein pairs from protein clusters/groups that arise mainly when homologous proteins from different taxa share peptides.

We also computed the average log fold changes between the three replicate mixture files for all taxa at all taxonomic levels. Because the three files are technical replicates, for each taxon at each taxonomic level, the expected log fold change between the files is zero. As expected, Figure 4 A indicates relatively tight distributions of log fold changes around zero for all species. One of the 24 species in the mixture (*Bacillus thuringiensis*) is missing in the figure. This species was identified in only one of the three replicates, and so it was not possible to calculate the protein fold changes for this species. MiCId’s failure to identify this species in two out of the three replicates can be attributed to the fact that it is very difficult to distinguish *Bacillus cereus* and *Bacillus thuringiensis* as their genetic differences are plasmid-based.^55^

**Figure 4:**
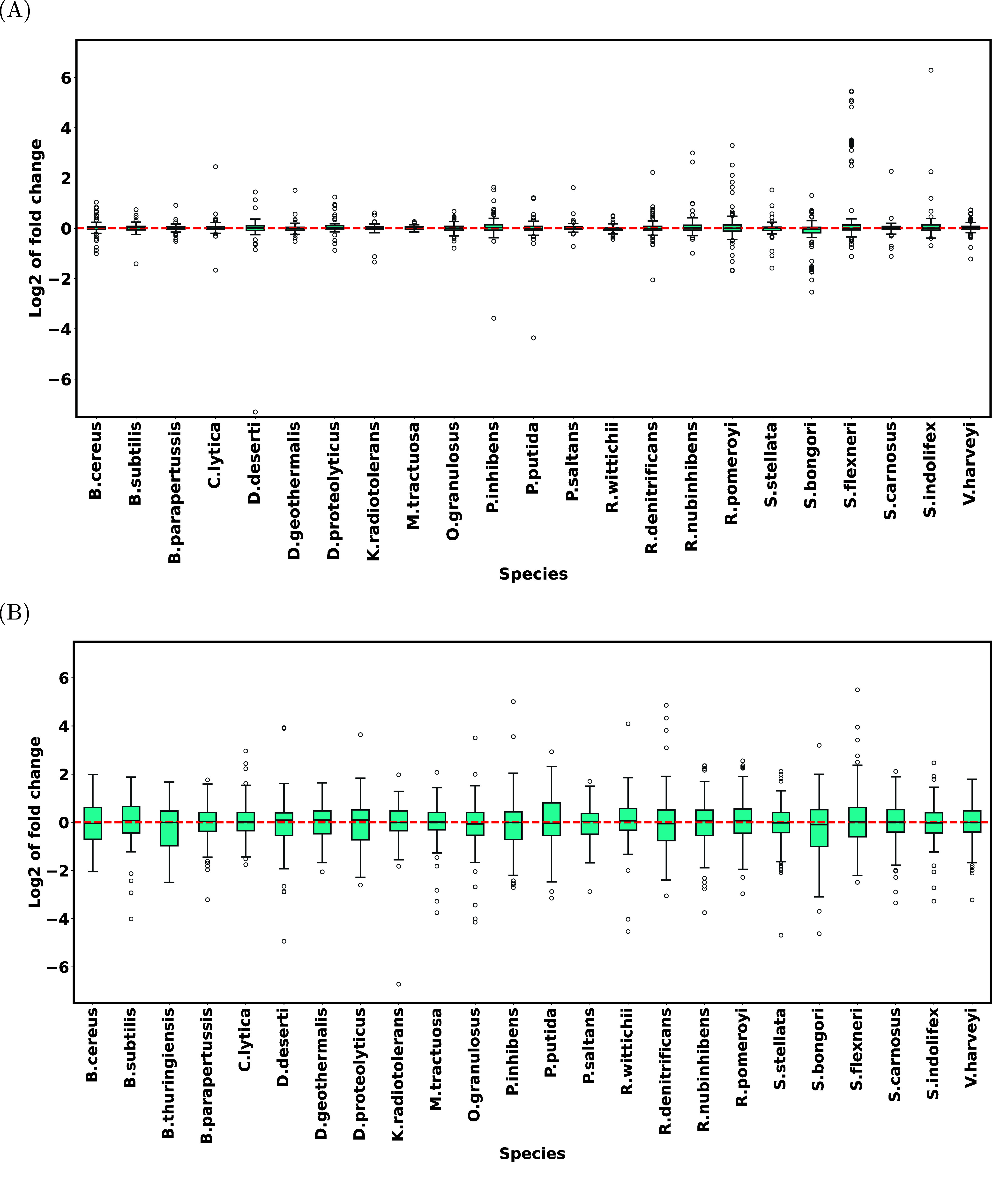
Log fold change comparison for Dataset 4 (PXD005776 and PXD005728). At the species level, the log fold changes are compared for each taxon between (A) technical replicates of the mixture and (B) the mixture and the species-specific files. To avoid long labels, the names of the species are abbreviated.

Figure 4 B shows the average log fold changes between the mixture and species-specific files. Again, the medians shown in Figure 4 B are close to the expected value, namely zero, although the distributions are not as tight as those shown in Figure 4 A. This may have to do with the fact that, due to shorter run times in the mixture, the number of identified peptides is significantly lower. The corresponding distributions at other taxonomic levels, centering around the expected value of zero, are shown in Figures S15–S19.

Of note, the fold change distributions are not compared between the combined and the species-specific cases. This is because there is no proper way to combine the peptide intensities in such a fake mixture.

### Assessment of MiCId Using a Clinical Human Stool Microbiome Dataset

As a final test, we evaluate our method using a clinical human stool microbiome dataset (Dataset 5; Table 1), originally studied by Zhang et al.^16^ It should be emphasized that the goal here is not providing insights into IBD, it is rather assessing MiCId. This is a challenging task as there is no true gold standard to compare to, there are many unknowns, and the results reported in the literature for IBD are sometimes conflicting (see the “Relative biomass of the identified taxa” subsection for some examples). It is therefore not possible to evaluate all findings based on MiCId results. Nonetheless, it is possible to use this dataset to assess MiCId in three different ways: (1) we show that, for all identified species, the median log fold changes (in protein abundances) between technical replicates are, as expected, close to zero and/or statistically insignificant, (2) we demonstrate that our results, in terms of relative biomass and protein abundances, largely agree with those reported in the original study^16^ that analyzed the same IBD dataset, and (3) using functional analyses, we show that many of the host (human) GO terms that we have identified as being enriched in IBD samples but not in the normal sample, have been reported in genomic studies to be indeed involved in IBD.

This dataset contains samples from four children with IDs HM541, HM604, HM609, and HM621. Out of these four, HM609 is normal but the remaining three patients have inflammatory bowel disease (IBD), with two diagnosed with ulcerative colitis (UC; HM604 and HM621), and HM541 having Crohn’s disease (CD). Since HM604 and HM621 have the same disease, we included only one of them (HM604) in our analyses.

### Distributions of the Numbers of Taxa in Clusters and Shared Peptides

Before assessing the identification/quantification results of MiCId, in this subsection we provide the distributions of the number of unique species in species-protein clusters (Figure S20) as well as those of the number of species-protein pairs sharing identified peptides (Figure S21). The distributions in Figure S20 suggest that approximately 22%, 34%, and 32% of the clusters in HM541, HM604, and HM609, respectively, contain multiple species. On the other hand, Figure S21 indicates that roughly 21%, 28%, and 30% of the peptides (respectively identified in HM541, HM604, and HM609) are shared between two or more species-protein pairs. (Note that these percentages are averaged over the replicates.) For each species, the numbers of identified proteins that are members of clusters containing multiple species are also plotted in Figures S22–S24 for HM541, HM604, and HM609, respectively. These figures show that most species have hundreds or tens of proteins that are in multispecies clusters. Once again, these findings affirm the importance of splitting the EIC (when peptides are shared) and demonstrate the usefulness of our approach.

### Relative Biomasses of the Identified Taxa

In this subsection, we compare the compositions of the three samples (HM541, HM604, and HM609), i.e., the relative biomasses of the taxa identified by MiCId at different taxonomic levels. The numbers of these taxa are given in Table 2. Since for each patient two or three technical replicates are available, the numbers given in Table 2 are averaged over the replicates. The average numbers of taxa with relative biomass of 0.01 or larger are given in parentheses in the table. It is important to note that, in terms of microorganism identification, MiCId has been rigorously tested in several other publications,^38–41,56,57^ showing high sensitivity and specificity. Therefore, in this paper MiCId’s ability to identify microorganisms is not evaluated.

**Table 2:** Average Number of Taxa Identified at Different Taxonomic Levels^a^

	Species	Genus	Family	Order	Class	Phylum
HM541	38.7 (18.0)	35.3 (14.0)	16.7 (7.0)	12.0 (7.0)	9.7 (6.0)	6.0 (4.0)
HM604	43.5 (18.0)	33.0 (15.0)	18.0 (8.0)	14.0 (6.5)	8.5 (5.5)	5.0 (4.5)
HM609	31.5 (19.0)	25.5 (15.5)	11.5 (7.5)	8.5 (7.0)	7.5 (6.0)	5.0 (4.0)

a The average numbers of taxa whose relative biomasses are larger than 0.01 are given in parentheses.

At the species level, the average relative biomasses are shown in Figure 5. For other taxonomic levels the corresponding plots are shown in Figures S25–S29. These figures demonstrate significant differences between the samples. To quantify the similarity between the samples’ compositions, for each pair of samples, we calculated the cosine similarity between the vectors containing the relative biomasses of all taxa, considering the relative biomasses of the unidentified taxa in each case to be zero. The mean cosine similarities (averaged over the replicates and rounded to three decimal points) are given in Table 3 for different taxonomic levels. The table indicates near perfect similarity between the technical replicates within a sample, but noticeably lower similarity between different samples, with HM604 and HM609 having the lowest similarity. That HM541 is more similar to HM609 (normal sample) is presumably due to the fact that patient HM541 was on therapy and in remission.^16^ Of note, many species identified in these samples have not yet been assigned proper scientific names in the NCBI database (the ones ending in “sp” as shown in Figure 5). Thus, it is not possible to compare the overlap between the taxa identified by MiCId and those identified in the original study.^16^ However, at the genus level this overlap is more than 90% (see ref 37 for more details).

**Figure 5:**
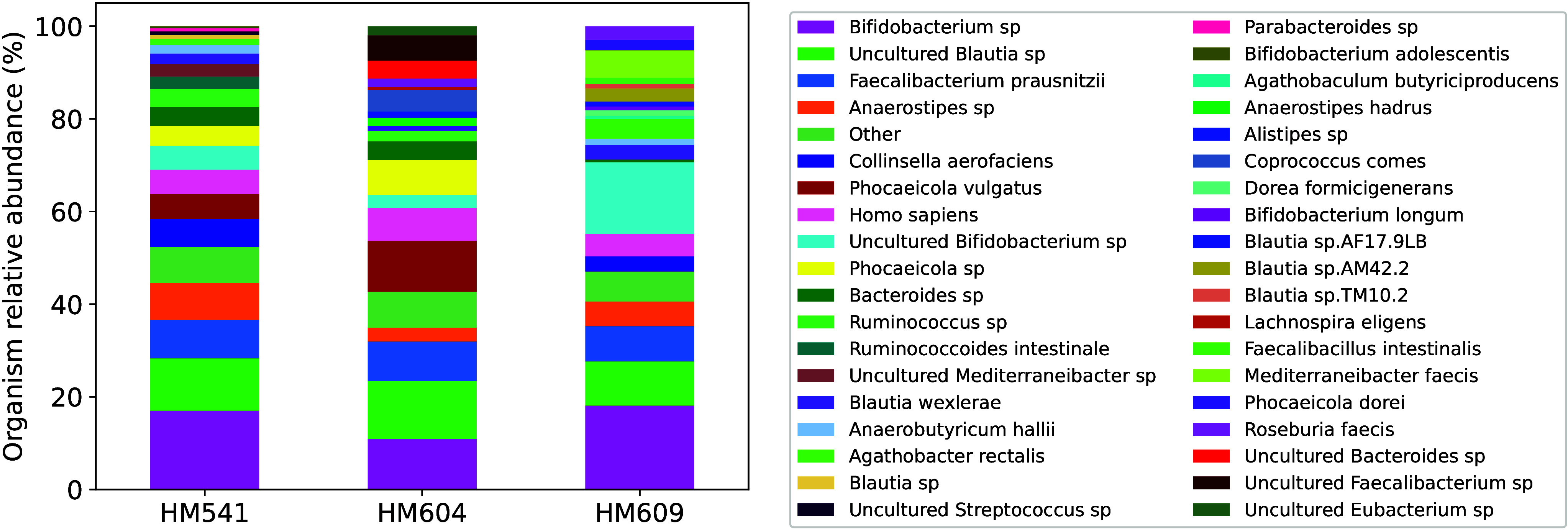
Species-level biomass plot. For each patient, the relative biomasses of different species are shown. In each case, the relative biomass of a species is averaged over the technical replicates. Species whose relative biomasses are lower than 0.01 are combined together and are referred to as “other” in the figure.

**Table 3:** Cosine Similarity between Compositions of the Three Samples at Different Taxonomic Levels

	Species	Genus	Family	Order	Class	Phylum
HM541–HM541	0.984	0.999	0.999	0.999	1.000	1.000
HM604–HM604	0.971	0.998	0.999	0.999	0.999	0.999
HM609–HM609	0.998	1.000	1.000	1.000	1.000	1.000
HM541–HM609	0.941	0.908	0.931	0.923	0.966	0.977
HM604–HM609	0.860	0.711	0.764	0.744	0.877	0.877
HM541–HM604	0.895	0.859	0.930	0.933	0.966	0.958

To see if the changes reported by MiCId and those published elsewhere agree, we compared our findings to those reported in the original study,^16^ which analyzed the same dataset and discussed the changes in the abundances of three species: *Faecalibacterium prausnitzii*, *Agathobacter rectalis* (also known as *Eubacterium rectale*), and *Bacteroides thetaiotaomicron*. *Faecalibacterium prausnitzii* is one of the most studied species in regard to CD and UC. Previous studies, however, provide conflicting conclusions about the role of this species in IBD and its biomass change relative to normal samples (see, for example, ref^16^ and references therein). In the original study of this dataset, Zhang et al. speculated that these conflicting reports were due to the differences in subspecies-level compositions of *Faecalibacterium prausnitzii* in various studies.^16^ These authors reported different behaviors for various strains of the species, with some strains decreasing and some increasing when comparing HM609 and HM604. On the other hand, HM609 and HM541 were reported to have similar subspecies compositions. The overall abundance of *Faecalibacterium prausnitzii* was reported to be slightly higher in HM604 than that in HM609, which in turn had a marginally higher abundance of this species in comparison with HM541. Our results show comparable abundances for this species in the three samples, although the biomasses are slightly higher in the diseased cases HM541 and HM604 (Figure 5). Zhang et al. reported strain-dependence of change of relative biomasses for *Agathobacter rectalis* and *Bacteroides thetaiotaomicron* as well. The overall biomass of *Agathobacter rectalis* was found to be higher in HM609 compared with the other two samples, which contained comparable abundances of this species. For *Bacteroides thetaiotaomicron*, however, the authors observed higher total (from all strains) biomass for HM604 in comparison with HM609 and HM541, which included comparable amounts of the species. In agreement with these results, Figure 5 shows higher relative biomass of *Agathobacter rectalis* for HM609 in comparison with that for HM604, for which the relative abundance of the species (0.014) is barely higher than 0.01. On the other hand, MiCId did not identify *Agathobacter rectalis* in HM541 at all. *Bacteroides thetaiotaomicron* was not identified by MiCId, so a comparison was not possible. However, at the genus level, Figure S25 shows the same trend observed by Zhang et al., i.e., higher relative abundance in HM604 when compared with the other samples.

We conducted a literature search to verify other changes in relative abundances shown in Figure 5. However, we found some conflicting reports. For instance, *Phocaeicola vulgatus* (also known as *Bacteroides vulgatus*) has been reported^58^ as a pathogen in UC patients, but some of its strains have been shown to be beneficial to patients with IBD. As another example, *Coprococcus comes* has been reported to be both less^59^ and more^60^ abundant in patients with Crohn’s disease. Moreover, for many species no information was found regarding their abundance differences in healthy and diseased samples. For these reasons, we decided to limit our discussion about change in relative abundances to the three cases investigated in the original paper, which used the same dataset.

### Taxon-Protein Quantification

The performance of MiCId in quantifying the identified taxon-protein pairs at each taxonomic level was also assessed. First, comparing technical replicates within each condition, for each taxa, the log fold changes were computed. At the species levels, for HM541, the distributions of log fold changes between the replicates are shown in Figure 6 A. The distributions at other taxonomic levels for this patient, and for the other two patients (HM604 and HM609) at all levels are plotted in Figures S30–S46. These figures demonstrate that, for the vast majority of taxa and regardless of the taxonomic level, the medians of log fold changes are close to zero as they should be (no significant changes should be observed between replicates). For example, for HM541 at the species level (Figure 6 A), the mean error, i.e., average of the absolute values of the medians, is 0.072. In this case, even the maximum error is small (0.342 for *Oscillibacter sp ER4*). In some cases, rather large (between 0.5 and 0.8) deviations from zero are observed (for instance, *Anaeromassilibacillus D41t1 190614 C2* in the HM604 sample; Figure S30). However, these deviations are not statistically significant (*E*-values > 0.01). In each case, statistical significance was assessed using the Wilcoxon signed-rank test. The resulting *P*-values were then multiplied by the number of comparisons to find the *E*-values (correction for multiple hypotheses testing). The *E*-values for all taxa at all taxonomic levels are reported in Tables S2–S7, Tables S8–S13, and Tables S14–S19 for HM541, HM604, and HM609, respectively.

**Figure 6:**
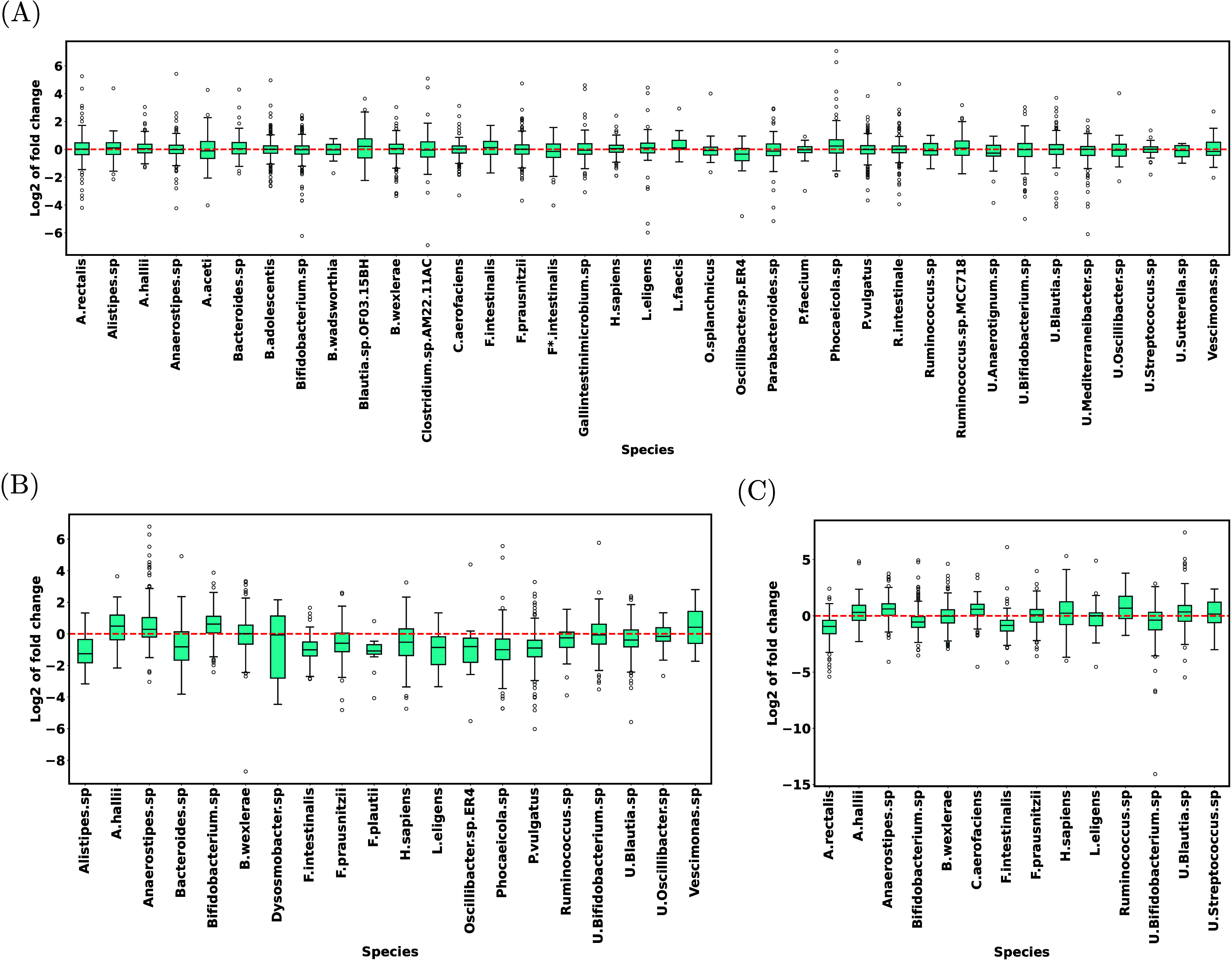
Distribution of log fold change for each species. The distributions compare the protein abundances in HM541 with those in (A) HM541, (B) HM604, and (C) HM609. Only distributions with more than 10 data points (proteins) are shown. The names of the species are abbreviated except for the ones that have not been yet named (the ones containing “.sp”), in which case the genus name has been fully spelled. In panel A, *Faecalibacillus intestinalis* and *Faecousia intestinalis* are abbreviated as *F.intestinalis* and F*.intestinalis, respectively. The “U.” at the beginning of some of the names denotes “Uncultured”. Full names of the species are given in Tables S2 (panel A), S32 (panel B), and S20 (panel C). Note that, unlike previous cases, in (B) and (C) the red lines do not show the expected medians, as they are unknown. They are provided to identify species whose proteins are overall up or down regulated. The following species undergo significant fold changes: (B) *Alistipes sp*, *Anaerostipes sp*, *Bacteroides sp*, *Bifidobacterium sp*, *F. intestinalis*, *F. prausnitzii*, *Homo sapiens*, *L. eligens*, *Phocaeicola sp*, *P. vulgatus*, *Uncultured Blautia sp* (Table S32), and (C) *A. rectalis*, *Anaerostipes sp*, *Bifidobacterium sp*, *C. aerofaciens*, *F. intestinalis*, and *Uncultured Bifidobacterium sp* (Table S20).

The log fold changes were also compared between the taxon-protein pairs identified for the three patients. For HM541, the resulting distributions, at the species level, are plotted in Figures 6 B and C, respectively comparing HM541 with HM604 and HM609. The distributions comparing HM604 and HM609 with each other are shown in Figure S47. The corresponding distributions for other taxonomic levels are plotted in Figures S48–S62. These analyses were limited to taxa that had at least 10 quantified taxon-protein pairs shared between the two conditions being compared. (Obviously, no such analyses can be done for a taxon that is present in one condition, but not in the other.)

Note that, unlike synthetic datasets, the expected values of the median log fold changes are not known in this case. Thus, it is not possible to assess MiCId based on the medians observed in Figures 6 B and C. However, one can investigate whether the changes shown in these figures are in agreement with previously reported observations. To this end, we first identified the taxa for which the median log fold change differed significantly from zero. Specifically, *E*-values were calculated as described in the previous paragraph, and changes with *E*-value <0.01 considered to be significant. For the three taxa discussed in the original study (see the “Relative biomass of the identified taxa” subsection), the median log fold changes and the corresponding *E*-values are given in Table 4. For all taxa at all taxonomic levels, the information is given in Tables S20–S25, Tables S26–S31, and Tables S32–S37 for HM541/HM609, HM604/HM609, and HM541/HM604 comparisons, respectively.

**Table 4:** Some of the Taxa Undergoing Significant Fold Changes between Conditions^a^

	Taxon	Median	IQR	N. proteins	*E*-value
HM541–HM604	Faecalibacterium prausnitzii	–0.59	1.18	213	4.6 × 10^–11^
HM604–HM609	Faecalibacterium prausnitzii	0.67	1.36	137	5.6 × 10^–8^
HM604–HM609	Bacteroides (genus level)	1.73	2.07	23	7.2 × 10^–4^
HM541–HM604	Bacteroides (genus level)	–0.66	1.88	76	5.5 × 10^–4^
HM541–HM609	Agathobacter rectalis	–0.95	1.18	185	2.9 × 10^–21^

a Median: Median of average log fold changes. IQR: Interquartile range of average log fold changes. A negative median means that the protein abundances are lower under the first condition.

Table 4 indicates largely the same trends as those suggested by the relative taxa abundances (Figure 5) and reported by Zhang et al. in the original study. For instance, the table suggests no significant changes between the abundances of *Faecalibacterium prausnitzii* proteins shared in HM541 and HM609. (Note that only cases with *E*-values smaller than 0.01 are listed in the table.) However, as reported by Zhang et al., a higher median abundance is observed for the proteins of *Faecalibacterium prausnitzii* in HM604 relative to both HM541 and HM609. Although the differences are not large, they are highly statistically significant (Table 4). This is interesting as the relative biomasses of *Faecalibacterium prausnitzii* in all three samples are comparable (Figure 5). This example shows that calculating taxon-protein pairs’ abundances can provide more information than taxa abundances. The rest of the trends shown in the table are in agreement with what is observed from Figure 5.

### Functional Analysis

Figures 6 A, B, C, and Table 4 compare the median abundances of all taxon-protein pairs (shared between the samples), and do not contain any information about individual proteins. A frequently used technique for gaining biological insights into the problem at hand is to find taxon-protein pairs whose abundances differ significantly under two or more different conditions. Such a set of taxon-protein pairs are often used for functional analysis to gain biological insights. This type of analysis is not possible for this dataset as for each condition data are available from only one patient and the technical replicates are too few for any meaningful statistical analysis. On the other hand, since there are significant composition differences between the samples (Figure 5), there are many missing values when quantifying taxon-protein pairs. Thus, an alternative approach was taken to perform functional analysis. Specifically, for each patient, only taxon-protein pairs identified/quantified in all of the corresponding replicates were considered. The log-transformed abundances of these taxon-protein pairs, at the species level, were then averaged over the replicates. The median of these averaged log abundances was then subtracted from each value to find patient-specific “log fold changes”. Finally, protein-taxon pairs were ranked, in descending order, based on the resulting values. We considered taxon-protein pairs with log fold change larger than 2, to have “high abundance”. The within-sample fold changes at different taxonomic levels are given in Tables S38–S43, Tables S44–S49, and Tables S50–S55 for HM541, HM604, and HM609, respectively.

We used the sets of high-abundance species-protein pairs and the Gene Ontology (GO)^51^ database to perform functional analyses (Fisher’s exact test; “biological processes” only) for each patient and each species. The identified significant GO terms are given in Tables S56–S58 for HM541, HM604, and HM609, respectively. Comparing two patients, we then focused on the GO terms found to be significant in one patient, but not in the other. These “differentially enriched” GO terms are given in Table 5 for HM541/HM609 and in Tables S59 and S60 for HM604/HM609 and HM541/HM604, respectively. Interestingly, in Table 5, almost all GO terms associated with changes in abundances of human proteins are enriched in HM541 (with IBD), and not in HM609 (normal). Similarly, except for GO:0005975 (carbohydrate metabolic process), GO:0006064 (glucuronate catabolic process), and GO:0006072 (glycerol-3-phosphate metabolic process), respectively associated with *Lachnospira intestinalis*, *Lachnospira eligens*, and *Anaerobutyricum hallii*, all other terms related to microorganisms are enriched in HM541, suggesting these biological processes are more active in the diseased case.

**Table 5:** GO Terms Identified in Sample HM541 (HM609) but Not in Sample HM609 (HM541)

Term	Description	*E*-value	Species	Sample
GO:0050829	defense response to Gram-negative bacterium	1.6 × 10^–9^	*Homo sapiens*	HM541
GO:0042742	defense response to bacterium	4.0 × 10^–9^	*Homo sapiens*	HM541
GO:0050832	defense response to fungus	1.4 × 10^–6^	*Homo sapiens*	HM541
GO:0061844	antimicrobial humoral immune response mediated by antimicrobial peptide	5.8 × 10^–6^	*Homo sapiens*	HM541
GO:0032717	negative regulation of interleukin-8 production	9.9 × 10^–6^	*Homo sapiens*	HM541
GO:0030593	neutrophil chemotaxis	2.1 × 10^–5^	*Homo sapiens*	HM541
GO:0006096	glycolytic process	2.6 × 10^–5^	*Collinsella aerofaciens*	HM541
GO:0019730	antimicrobial humoral response	4.4 × 10^–4^	*Homo sapiens*	HM541
GO:0045087	innate immune response	5.5 × 10^–4^	*Homo sapiens*	HM541
GO:0005975	carbohydrate metabolic process	6.0 × 10^–4^	*Lachnospira intestinalis*	HM609
GO:0006954	inflammatory response	6.9 × 10^–4^	*Homo sapiens*	HM541
GO:0002523	leukocyte migration involved in inflammatory response	7.2 × 10^–4^	*Homo sapiens*	HM541
GO:0006096	glycolytic process	8.5 × 10^–4^	*Bifidobacterium adolescentis*	HM541
GO:0006096	glycolytic process	9.4 × 10^–4^	*Anaerostipes sp*	HM541
GO:0032119	sequestering of zinc ion	1.2 × 10^–3^	*Homo sapiens*	HM541
GO:0070488	neutrophil aggregation	1.2 × 10^–3^	*Homo sapiens*	HM541
GO:0035606	peptidyl-cysteine S-trans-nitrosylation	1.2 × 10^–3^	*Homo sapiens*	HM541
GO:0042119	neutrophil activation	1.3 × 10^–3^	*Homo sapiens*	HM541
GO:0006096	glycolytic process	1.4 × 10^–3^	*Bifidobacterium sp*	HM541
GO:0006064	glucuronate catabolic process	1.8 × 10^–3^	*Lachnospira eligens*	HM609
GO:0006520	cellular amino acid metabolic process	2.4 × 10^–3^	*Odoribacter splanchnicus*	HM541
GO:0006094	gluconeogenesis	2.5 × 10^–3^	*Bacteroides sp*	HM541
GO:0003094	glomerular filtration	2.6 × 10^–3^	*Homo sapiens*	HM541
GO:0042853	l-alanine catabolic process	2.9 × 10^–3^	*Bilophila wadsworthia*	HM541
GO:0031640	killing of cells of another organism	3.3 × 10^–3^	*Homo sapiens*	HM541
GO:0006425	glutaminyl-tRNA aminoacylation	3.4 × 10^–3^	*Faecalibacillus intestinalis*	HM541
GO:0002227	innate immune response in mucosa	3.5 × 10^–3^	*Homo sapiens*	HM541
GO:0002215	defense response to nematode	3.6 × 10^–3^	*Homo sapiens*	HM541
GO:0006909	phagocytosis	4.1 × 10^–3^	*Homo sapiens*	HM541
GO:0006520	cellular amino acid metabolic process	4.2 × 10^–3^	*Oscillibacter sp ER4*	HM541
GO:0060267	positive regulation of respiratory burst	5.3 × 10^–3^	*Homo sapiens*	HM609
GO:0022900	electron transport chain	6.3 × 10^–3^	*Uncultured Oscillibacter sp*	HM541
GO:0031340	positive regulation of vesicle fusion	7.2 × 10^–3^	*Homo sapiens*	HM541
GO:0006096	glycolytic process	7.7 × 10^–3^	*Parabacteroides sp*	HM541
GO:0006090	pyruvate metabolic process	9.5 × 10^–3^	*Faecousia intestinalis*	HM541
GO:0006072	glycerol-3-phosphate metabolic process	9.8 × 10^–3^	*Anaerobutyricum hallii*	HM609

Table 5 indicates that most identified GO terms are related to the host (human), nearly half of which are involved in defense/immune/antimicrobial response. In genomic studies, many of these terms have been reported to be associated with IBD (UC and/or CD), including GO:0042742 (defense response to bacterium),^61^ GO:0045087 (innate immune response),^61^ GO:0006954 (inflammatory response),^61,62^ GO:0019730 (antimicrobial humoral response),^63^ and GO:0061844 (antimicrobial humoral immune response mediated by antimicrobial peptide).^63^ Other host-related terms listed in Table 5, and reported as being involved in IBD, are GO:0030593 (neutrophil chemotaxis)^62,63^ and GO:0006909 (Phagocytosis).^63,64^ The good overlap between the list of identified GO terms (for the host) and those reported in previous studies is indicative of MiCId’s ability to identify abundant human proteins. In fact, in the original study, Zhang et al. identified 35 human proteins highly abundant in all three samples. We found most of these proteins to have high abundance (as defined above) as well, with 31, 31, and 26 proteins identified respectively in HM541, HM604, and HM609.

Importantly, MiCId’s ability to resolve taxon-protein clusters and estimate taxon-specific protein abundances enabled the identification of high-abundance taxon-protein pairs across all detected species, and consequently functional analyses for the microorganisms. However, an assessment of bacterial GO terms identified by MiCId was not possible. Several studies have performed functional analysis for human-protein pairs, but our search did not find any studies that have performed such analyses for identified bacterial proteins at the species level (although our search was not exhaustive). Notably, the host or bacterial entries in Table 5 lacking literature support may be particularly interesting, as they could provide new insights into the biological processes underlying IBD. It is also interesting to note that the three taxa discussed in the original^16^ (see Table 4) study are not present in Table 5. This indicates that, for these taxa, there are not enough high abundance proteins related to the same function (Tables S56–S58), which is also consistent with the fact that median log fold changes between samples (Table 4), although significant, are not that large. It should be noted that in the original study functional analyses were performed differently and, unlike this study, they were not meant to find differentially enriched terms. Thus, in this regard, we do not compare our results with those of that study.

Comparing HM604 vs HM609, Table S59 contains mostly the same terms as Table 5, with 18 out of the 26 terms listed in Table S59 (including most of the ones for which we found evidence of involvement in IBD) being also reported in Table 5. This implies that, in terms of differentially enriched GO terms, HM541 and HM604 are similar. This similarity between the results for HM541 and HM604 is expected as UC and CD are both subtypes of IBD. However, there are some terms in Table 5 that are missing in Table S59 and vice versa. These terms (Comparing HM604 vs HM541) are given in Table S60.

### Limitations of MiCId

At present, MiCId does not contain a normalization method for quantified taxon-protein pairs, which is why we employed directLFQ in this study. This limitation, which will be addressed in future releases, does not hinder the use of MiCId, since its output files can be readily converted into the appropriate input format for directLFQ, a package that is both accessible and easy to use. Another current limitation of MiCId is its reliance on NCBI resources, offering biological functional annotation of taxon–protein pairs only through GO terms extracted from the NCBI database. However, users can exploit the protein identifiers associated with the quantified taxon–protein pairs reported by MiCId to query alternative functional annotation resources, such as the Enzyme Commission (EC),^65^ Kyoto Encyclopedia of Genes and Genomes (KEGG),^66^ and Clusters of Orthologous Genes (COG)^67^ databases. These limitations will also be resolved in future versions of MiCId.

## Conclusions

MiCId has been improved in many ways since it was first introduced,^38^ and has been shown to have great identification specificity and sensitivity.^39–41,56,57^ In this paper, we present an assessment of the recently added functionality, that is protein quantification at different taxonomic levels. Using simple synthetic datasets (Datasets 1–3) with known relative species abundances, we show that species-protein pair fold changes predicted by MiCId have distributions that agree with the known expected values and those predicted by MaxQuant. Employing a more complex 24-species synthetic dataset (Dataset 4), we demonstrate MiCId’s ability of accurately splitting the EIC between taxon-protein pairs that share peptides, and so cluster together, but belong to different species/taxa. Finally, utilizing Dataset 5 containing data from clinical human stool microbiome, we show that MiCId accurately reproduces the expected median log fold changes when comparing technical replicates, and produces results that largely agree with those reported previously. Moreover, functional analyses based on MiCId’s results for Dataset 5 leads to several differentially enriched GO terms that have been reported to be implicated in IBD. These observations indicate an overall good performance by MiCId in quantifying proteins when the sample contains many species that might have shared peptides.

This work introduces a novel method for protein analysis in metaproteomics, and we anticipate that its integration into MiCId’s workflow will benefit the MS-based metaproteomics community. To ensure accessibility, the approach has been fully implemented in MiCId, whose workflow, source code (C++), GUI, and executables are freely available for Linux at https://www.ncbi.nlm.nih.gov/CBBresearch/Yu/downloads.html.

## Supplementary Material





## Notes

Text S1 on detailed mathematical description of the EM algorithm, Figure S1 showing log fold changes (MaxQaunt) for Dataset 1, Figures S2–S6 showing log fold changes at different levels (MiCId) for Dataset 1, Figures S7 and S8 showing log fold changes (MiCId) for Datasets 2 and 3, Figure S8 showing distribution of number of shared peptides for Dataset 4, Figures S10–S14 showing SetUp plots at different levels (Dataset 4), Figures S15–S19 showing log fold changes (Dataset 4), Figure S20 showing distribution of number of species in clusters, Figure S21 showing distribution of number protein-species pairs sharing a peptide, Figures S22–S24 showing number of proteins of a species in multispecies clusters, Figures S25–S29 showing samples’ compositions at different levels, Figures S30–S62 showing within-sample and between-sample log fold changes at different taxonomic levels (PDF)
Table S1 showing list of microorganisms in the database, Tables S2–S37 listing within-sample and between-sample log fold changes at different taxonomic levels, Tables S38–S55 showing lists of high abundance taxon-protein pairs, Tables S56–S58 showing lists of enriched GO terms for each sample, Tables S59 and S60 showing lists of differentially enriched GO terms (XLSX)
