# Supplementary material for: Taxonomic-Level Protein Quantification in Metaproteomics Using a Biomass-Constrained Expectation–Maximization Approach
Source: J Am Soc Mass Spectrom. 2026 Jan 15;37(2):424–39. doi: 10.1021/jasms.5c00332 (PMC12879945; doi:10.1021/jasms.5c00332)
Supplement: Supplementary file 1 [file js5c00332_si_001.pdf]

Supporting Information. “Taxonomic-Level Protein Quantification in Metaproteomics Using a Biomass-Constrained Expectation–Maximization Approach”

Gelio Alves,* Mehdi B. Hamaneh,* Aleksey Y. Ogurtsov, and Yi-Kuo Yu*

Division of Intramural Research, National Library of Medicine, National Institutes of Health, Bethesda, MD 20894, USA

E-mail: alves@ncbi.nlm.nih.gov; bagherih@ncbi.nlm.nih.gov; yyu@ncbi.nlm.nih.gov

Phone: +1 301-435-5989

Text S1: Detailed Computational Framework for the Modified EM Algorithm Incorporating Biomass Constraints

We begin by establishing the required notation. In a metaproteomics experiment, thousands of peptides can be identified. Let $\Pi = \{\pi_1, \pi_2, \ldots, \pi_N\}$ represent a set of N non-redundant peptides, each identified and mapped to proteins. Assume that each π_i has been observed with EIC denoted by n_i. The M taxa included at a specified taxonomy level are indexed by α, where t_α represents the αth taxon

Let the variable $1 \leq k \leq K$ index the K proteins, and d_α denote the peptidome of taxon t_α. Let $p(\pi_{i,k} \leftarrow t_\alpha) \geq 0$ denote the probability that π_i, appearing in protein k, originated from taxon t_α. Also, let $n_{i,k}$ be the EIC of $\pi_{i,k}$, and $0 \leq n_{i,k,\alpha} \leq n_{i,k}$ represent the EIC of π_i in protein k coming from taxon t_α. Clearly, the following conditions must hold: $\sum_\alpha n_{i,k,\alpha} = n_{i,k}$, and $\sum_{k=1}^{K} n_{i,k} = n_i$.

Since our application is not limited to using taxon-specific peptides, the taxon from which π_i originates is not known in advance. Therefore, the values of $n_{i,k,\alpha}$ are treated as hidden variables. The objective of the expectation maximization algorithm is to estimate the parameters $p(\pi_{i,k} \leftarrow t_\alpha)$ that maximize the likelihood of the observed data n_i. Clearly, $\sum_\alpha p(\pi_{i,k} \leftarrow t_\alpha) I(\pi_i \in d_\alpha) = p(\pi_{i,k}) = \frac{n_{i,k}}{\sum_j n_{j,k}}$ where $p(\pi_{i,k})$ represents the prior probability that peptide π_i is associated with protein k, and the indicator function $I(\pi_i \in d_\alpha)$ equals 1 if π_i belongs to the peptidome of t_α and 0 otherwise. As might be expected, we have that $\sum_{k=1}^{K} p(\pi_{i,k}) = p(\pi_i)$, which is the prior probability of peptide π_i, and $\sum_{i=1}^{N} p(\pi_i) = 1$.

Here, we introduce the likelihood function. Assuming independence among the occurrences of identified peptides, we can express the likelihood function for the hidden variables $n_{i,k,\alpha}$, consistent with the observed data $n_{i,k}$ (assuming that the parameters $p(\pi_{i,k} \leftarrow t_\alpha)$ are known), as follows

$$L\left(\{n_{i,k,\alpha}\} | \{p(\pi_{i,k} \leftarrow t_\alpha)\}\right) = \frac{\left(\sum_{i=1}^{N} \sum_{k=1}^{K} n_{i,k}\right)!}{\prod_{i'=1}^{N} \prod_{k'=1}^{K} \prod_{\alpha=1}^{M} n_{i',k',\alpha}!} \prod_{i'=1}^{N} \prod_{k'=1}^{K} \prod_{\alpha=1}^{M} p(\pi_{i',k'} \leftarrow t_\alpha)]^{n_{i',k',\alpha}} \tag{S1}$$

The first step of EM is to compute the expected values of the hidden variables. Because $n_{i,k} = \sum_\alpha n_{i,k,\alpha} I(\pi_i \in d_\alpha)$, the expected value of $n_{i,k,\alpha}$ for a fixed i can be easily computed for any t_α whose peptidome contain π_i. Let $\varsigma(i,k)$ denote a realization of $\{n_{i,k,\alpha}\}$ satisfying $n_{i,k} = \sum_\alpha n_{i,k,\alpha} I(\pi_i \in d_\alpha)$. The expected value of $n_{i,k,\beta}$ can be written as

$$\begin{aligned} E[n_{i,k,\beta} | \{p(\pi_{j,k} \leftarrow t_\alpha)\}] &= \frac{\sum_{\varsigma(i,k)} L\left(\{n_{i,k,\alpha}\} | \{p(\pi_{j,k} \leftarrow t_\alpha)\}\right) n_{i,k,\beta}}{\sum_{\varsigma(i,k)} L\left(\{n_{i,k,\alpha}\} | \{p(\pi_{j,k} \leftarrow t_\alpha)\}\right)} \\ &= \frac{\partial \ln[\sum_{\varsigma(i,k)} L\left(\{n_{i,k,\alpha}\} | \{p(\pi_{j,k} \leftarrow t_\alpha)\}\right)]}{\partial \ln[p(\pi_{i,k} \leftarrow t_\beta)]} \end{aligned} \tag{S2}$$

Because

$$\sum_{\varsigma(i,k)} L\left(\{n_{i,k,\alpha}\}|\{p(\pi_{j,k}\leftarrow t_\alpha)\}\right) \propto \left[\sum_\alpha p(\pi_{i,k}\leftarrow t_\alpha) I(\pi_i \in d_\alpha)\right]^{n_{i,k}}$$

we find

$$E[n_{i,k,\beta}|\{p(\pi_{j,k}\leftarrow t_\alpha)\}] = n_{i,k}\frac{p(\pi_{i,k}\leftarrow t_\beta)I(\pi_i \in d_\beta)}{\sum_\alpha p(\pi_{i,k}\leftarrow t_\alpha)I(\pi_i \in d_\alpha)}\,. \tag{S3}$$

In the second step (maximization), one finds the next set of $\{p^{(\ell+1)}(\pi_{i,k}\leftarrow t_\alpha)\}$ to replace the previous set of $\{p^{(\ell)}(\pi_{i,k}\leftarrow t_\alpha)\}$ that was used to compute the expected values $E[n_{i,k,\beta}|\{p^{(\ell)}(\pi_{j,k}\leftarrow t_\alpha)\}]$. This is done by maximizing the log likelihood in Eq. (S1) (with the $n_{i,k,\alpha}$s replaced by their expected values and treated as known numbers) via differentiation with respect to $\{p(\pi_{i,k}\leftarrow t_\alpha)\}$. There is of course a constraint that $1 = \sum_{i=1}^N p(\pi_i) = \sum_{i=1}^N \sum_{\alpha=1}^M p(\pi_i \leftarrow t_\alpha) = \sum_{i=1}^N \sum_{k=1}^K \sum_{\alpha=1}^M p(\pi_{i,k} \leftarrow t_\alpha)$. Introducing this constraint via a Lagrange multiplier, one ends up having

$$p^{(\ell+1)}(\pi_{i,k}\leftarrow t_\alpha) = \frac{E[n_{i,k,\alpha}|\{p^{(\ell)}(\pi_{j,k}\leftarrow t_\beta)\}]}{\sum_{i=1}^N \sum_{k=1}^K \sum_{\alpha=1}^M E[n_{i,k,\alpha}|\{p^{(\ell)}(\pi_{j,k}\leftarrow t_\beta)\}]}\,. \tag{S4}$$

A few considerations lead us to take a simplified form of the above EM procedure. First, it requires a lot of data to faithfully estimate $\{p(\pi_{i,k}\leftarrow t_\alpha)\}$. Therefore, we make the simple choice that

$$p(\pi_{i,k}\leftarrow t_\alpha) \Rightarrow p(\pi_i\leftarrow t_\alpha)p(k|t_\alpha) \Rightarrow p(t_\alpha)p(\pi_i)p(k|t_\alpha) \tag{S5}$$

to reduce the number of parameters to be fitted.

Second, even though peptide π_i is identified, there is no good way to infer its total ion count n_i as there might be multiple identifications (some more confident and some less confident) or unfragmented MS^1 ions of the same peptide π_i. To simplify, we retain only the most confident identification per peptide weighted by the identification confidence

$$Z[E(\pi_i)] = \frac{n_i}{1 + E(\pi_i)/E_c} \equiv z_i. \tag{S6}$$

Basically, we replace n_i by z_i and $n_{i,k,\alpha}$ ($n_{i,k}$) by $z_{i,k,\alpha}$ ($z_{i,k}$). Here E_c is the E-value cutoff used to control the expected number of false positives (FPs). The expected number of FP peptides identified is strongly controlled to be no more than 100 by setting E_c equal to 100 divided by the total number of MS/MS spectra.

With the simplifications and modifications above, Eq. (S3) becomes

$$\begin{aligned} E[z_{i,k,\beta}|\{p(t_\alpha), p(k|t_\alpha)\}] &= z_{i,k}\frac{p(t_\beta)p(\pi_i)p(k|t_\beta)I(\pi_i \in d_\beta)}{\sum_\alpha p(t_\alpha)p(\pi_i)p(k|t_\alpha)I(\pi_i \in d_\alpha)} \\ &= z_{i,k}\frac{p(t_\beta)p(k|t_\beta)I(\pi_i \in d_\beta)}{\sum_\alpha p(t_\alpha)p(k|t_\alpha)I(\pi_i \in d_\alpha)}\,. \end{aligned} \tag{S7}$$

Before proceeding to maximization, we first note that the number of variables has decreased substantially under the simplification.

Here, we present a simplified form of the likelihood function in Eq. (S1). Assuming

independence among the occurrences of identified peptides, we can express the simplified likelihood function for the hidden variables $n_{i,k,\alpha}$, consistent with the observed data $n_{i,k}$, as follows

$$
\begin{aligned}
L\left(\{z_{i,k,\alpha}\}|\{p(\pi_{i,k}\leftarrow t_\alpha)\}\right) &\Rightarrow G(z_{i,k,\alpha}) \prod_{i=1}^{N}\prod_{k=1}^{K}\prod_{\alpha=1}^{M}[p(\pi_i)p(t_\alpha)p(k|t_\alpha)]^{z_{i,k,\alpha}} \\
&= G(z_{i,k,\alpha}) \left\{\prod_{i=1}^{N}[p(\pi_i)]^{z_i}\right\}\left\{\prod_{\alpha=1}^{M}[p(t_\alpha)]^{\sum_i z_{i,\alpha}}\right\}\left\{\prod_{k=1}^{K}\prod_{\alpha=1}^{M}[p(k|t_\alpha)]^{\sum_i z_{i,k,\alpha}}\right\} \\
&\equiv G(z_{i,k,\alpha}) \cdot L_0(\{z_i\}|\{p(\pi_i)\}) \cdot L_1(\{z_{i,\alpha}\}|\{p(t_\alpha)\}) \cdot L_2(\{z_{i,k,\alpha}\}|\{p(k|t_\alpha)\}), \qquad \text{(S8)}
\end{aligned}
$$

where $G(z_{i,k,\alpha})$ is given by

$$
G(z_{i,k,\alpha}) = \frac{\Gamma(1+\sum_i\sum_k z_{i,k})}{\prod_i\prod_k\prod_\alpha \Gamma(z_{i,k,\alpha}+1)}.
$$

In our implementation of the EM algorithm with constraints, we first maximize the likelihood function L_1, subject to the constraint $\sum_\alpha p(t_\alpha) = 1$, to obtain the recursion for $p(t_\alpha)$ until convergence. During this process, the prior $p(\pi_i)$ for each π_i is initially set to $1/N$. Setting

$$
0 = \frac{d\ln L_1}{d\,p(t_\alpha)}
$$

gives

$$
\begin{aligned}
p^{(\ell+1)}(t_\alpha) &\propto \sum_{i=1}^{N} E[z_{i,\alpha}|\{p(t_\alpha)\}] \\
&= \sum_{i=1}^{N} z_i \frac{p(t_\alpha)I(\pi_i\in d_\alpha)}{\sum_\beta p(t_\beta)I(\pi_i\in d_\beta)} \equiv C_\alpha^{(\ell+1)}. \qquad \text{(S9)}
\end{aligned}
$$

Along with the constraint condition $\sum_\alpha p(t_\alpha) = 1$, this leads to

$$
p^{(\ell+1)}(t_\beta) \Rightarrow \frac{C_\beta^{(\ell+1)}}{\sum_\alpha C_\alpha^{(\ell+1)}}. \qquad \text{(S10)}
$$

Once the values of $p(t_\alpha)$'s have converged in Eq. S9, we then proceed to maximize L_2 with respect to $p(k|t_\alpha)$, subject to the constraint that $\sum_k p(k|t_\alpha) = 1\ \forall\ \alpha$. Setting

$$
0 = \frac{d\ln L_2}{d\,p(k|t_\alpha)}
$$

leads to

$$\begin{aligned} p^{(\ell+1)}(k|t_\alpha) &\propto \sum_{i=1}^{N} E[z_{i,k,\alpha}|\{p(t_\alpha),\ p^{(\ell)}(k|t_\alpha)\}] \\ &= \sum_{i=1}^{N} z_{i,k} \frac{p(t_\alpha)p^{(\ell)}(k|t_\alpha)I(\pi_i \in d_\alpha)}{\sum_\beta p(t_\beta)p^{(\ell)}(k|t_\beta)I(\pi_i \in d_\beta)} \equiv C_{k,\alpha}^{(\ell+1)}, \end{aligned} \tag{S11}$$

The constraints conditions $\sum_k p(k|t_\alpha) = 1$ lead to

$$p^{(\ell+1)}(r|t_\beta) \Rightarrow \frac{C_{r,\beta}^{(\ell+1)}}{\sum_k C_{k,\alpha}^{(\ell+1)}} \,. \tag{S12}$$

In summary, during the first round of the EM algorithm (biomass estimation) for the L_1 part, the priors $p(t_\alpha)$ are initialized to $1/M$, where M is the total number of identified taxa. The expectation and maximization steps are repeated until numerical convergence is achieved for the priors $p(t_\alpha)$. Once the taxon priors are determined, the algorithm proceeds to the second round, L_2, of the EM algorithm. Here, the $p(k|t_\alpha)$ values are initialized to $1/K$, where K is the total number of proteins. The computed expected probabilities $p(t_\alpha)$ can be interpreted as the relative biomasses of the identified taxa (t_α), whereas the estimated $p(k|t_\alpha)$ represent the relative biomasses contributions of specific taxon–protein pairs (i.e., protein k belonging to taxon t_α).

Figure S1

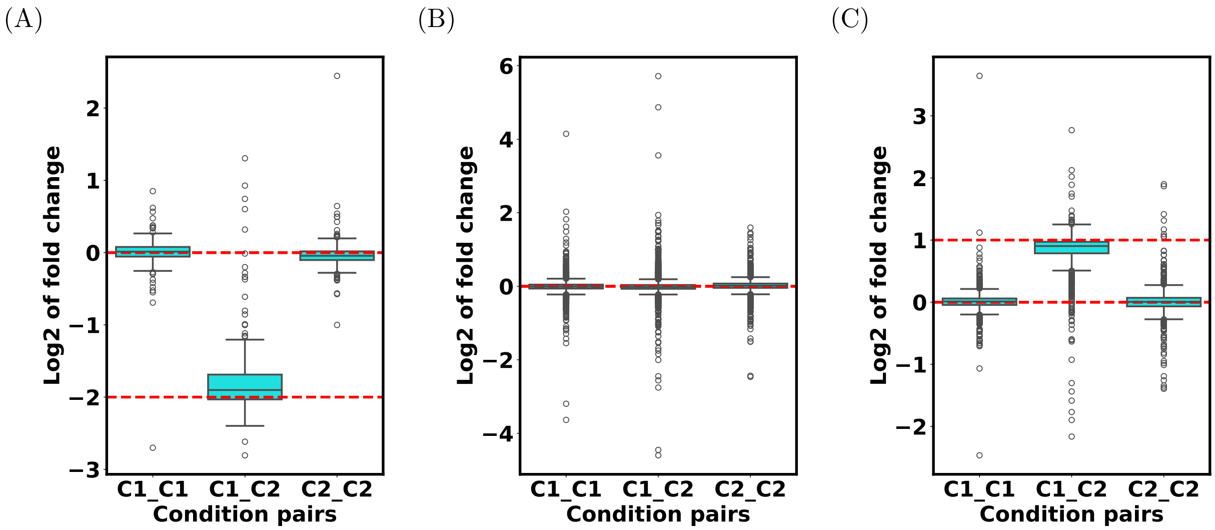

Figure S1: Species-level protein log fold changes for Dataset 1 obtained using MaxQuant. Box plots of distributions of average log fold changes are shown for (A) E.coli, (B) Human, and (C) Yeast for Dataset 1 (PXD028735). Comparing this figure with Figure 2 of the main text indicates comparable performances for MaxQuant+directLFQ and MiCId+directLFQ.

Figure S2

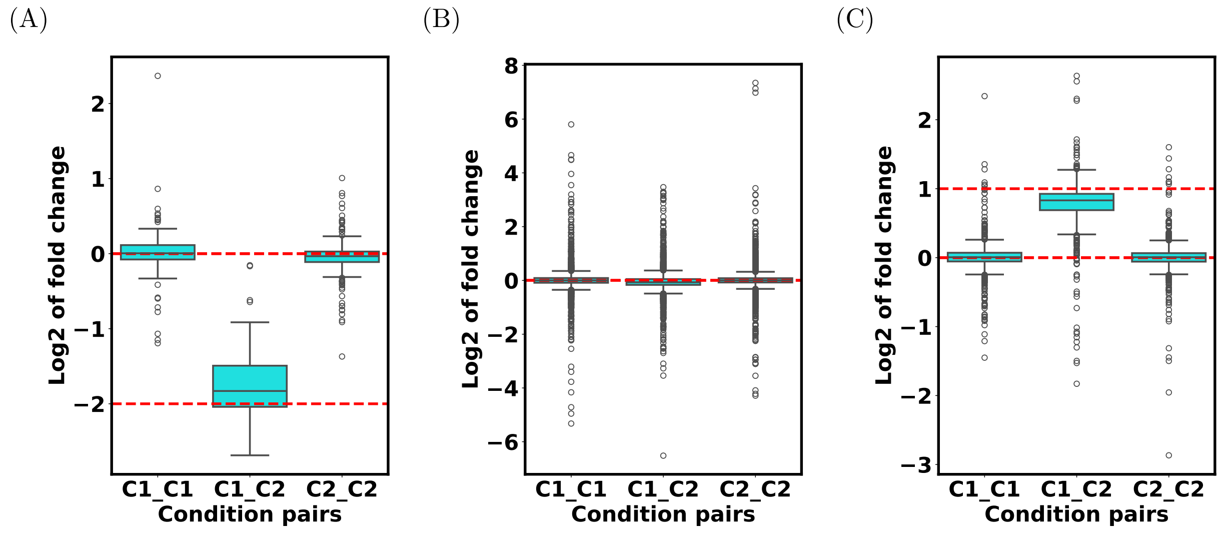

Figure S2: Genus-level protein log fold changes for Dataset 1. Box plots of distributions of average log fold changes are shown for (A) Escherichia, (B) Homo, and (C) Saccharomyces for Dataset 1 (PXD028735). Comparing this figure with Figure 2 of the main text indicates virtually identical distributions.

Figure S3

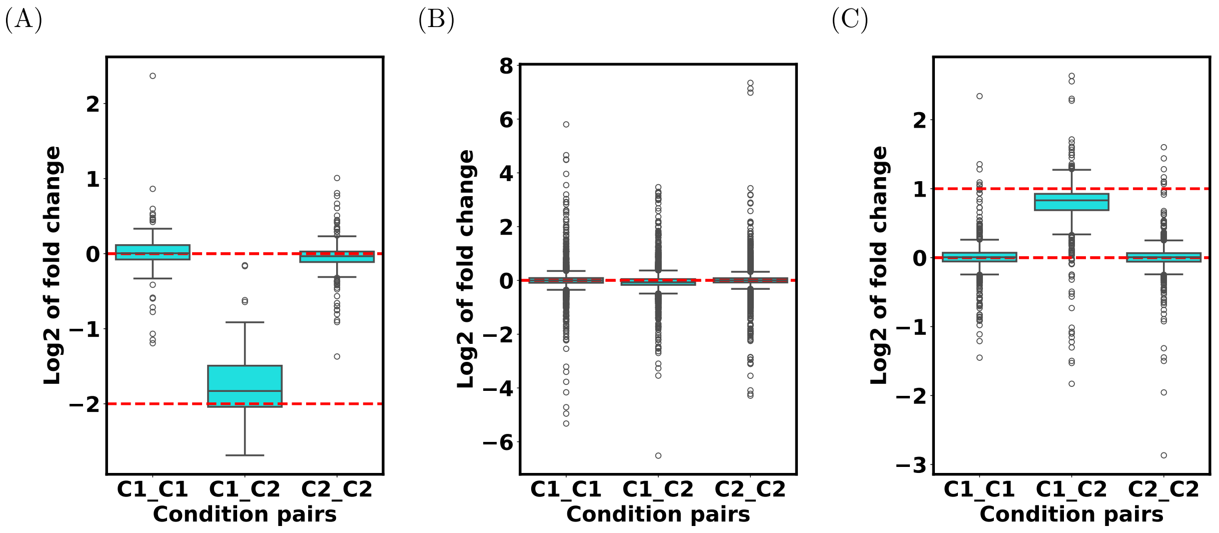

Figure S3: Family-level protein log fold changes for Dataset 1. Box plots of distributions of average log fold changes are shown for (A) Enterobacteriaceae, (B) Hominidae, and (C) Saccharomycetaceae for Dataset 1 (PXD028735). Comparing this figure with Figure 2 of the main text indicates virtually identical distributions.

Figure S4

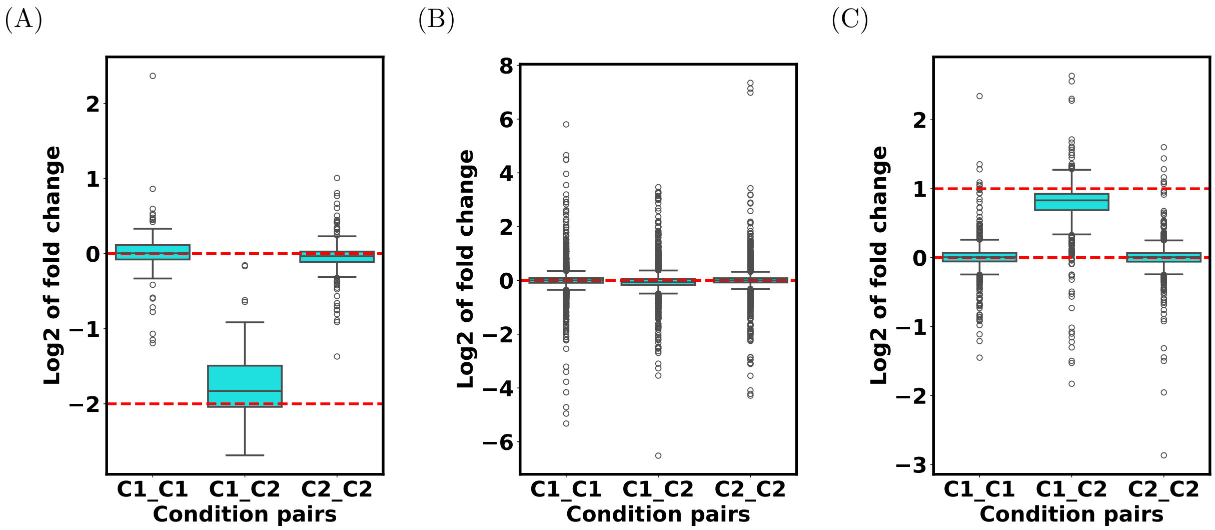

Figure S4: Order-level protein log fold changes for Dataset 1. Box plots of distributions of average log fold changes are shown for (A) Enterobacterales, (B) Primates, and (C) Saccharomycetales for Dataset 1 (PXD028735). Comparing this figure with Figure 2 of the main text indicates virtually identical distributions.

Figure S5

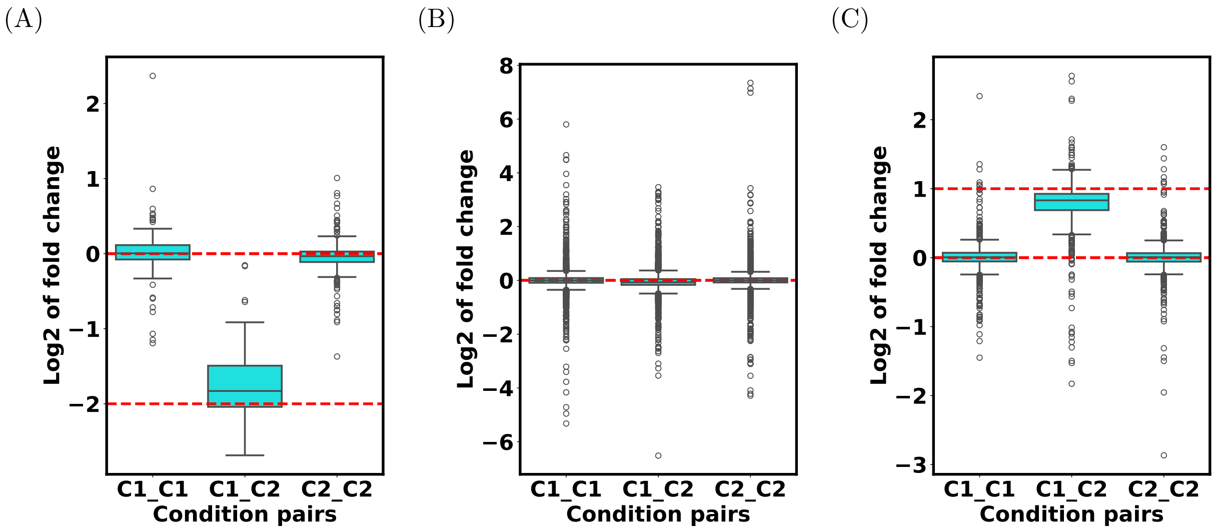

Figure S5: Class-level protein log fold changes for Dataset 1. Box plots of distributions of average log fold changes are shown for (A) Gammaproteobacteria, (B) Mammalia, and (C) Saccharomycetes for Dataset 1 (PXD028735). Comparing this figure with Figure 2 of the main text indicates virtually identical distributions.

Figure S6

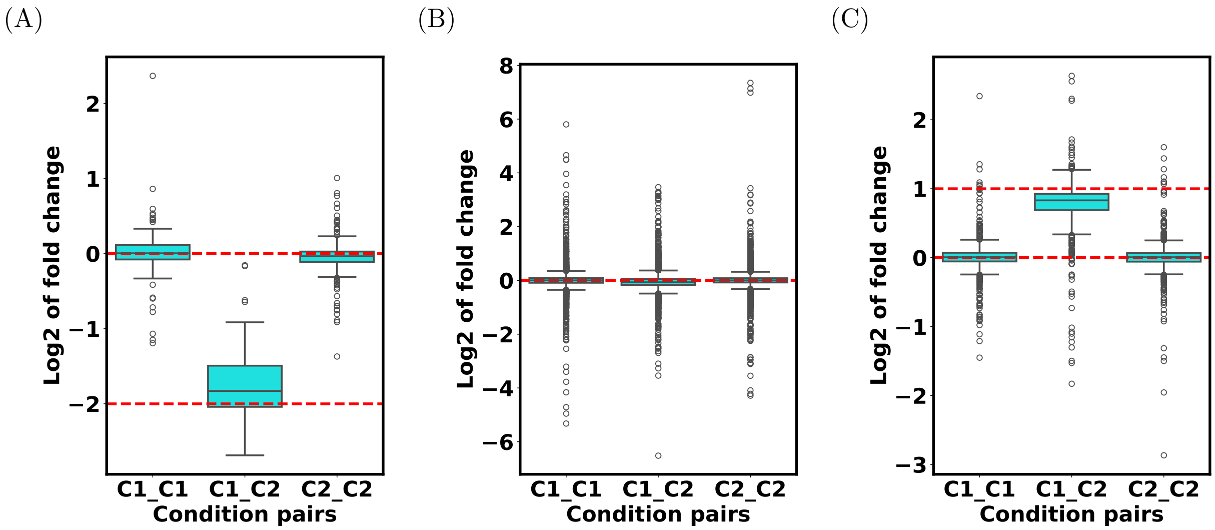

Figure S6: Phylum-level protein log fold changes for Dataset 1. Box plots of distributions of average log fold changes are shown for (A) Pseudomonadota, (B) Chordata, and (C) Ascomycota for Dataset 1 (PXD028735). Comparing this figure with Figure 2 of the main text indicates virtually identical distributions.

Figure S7

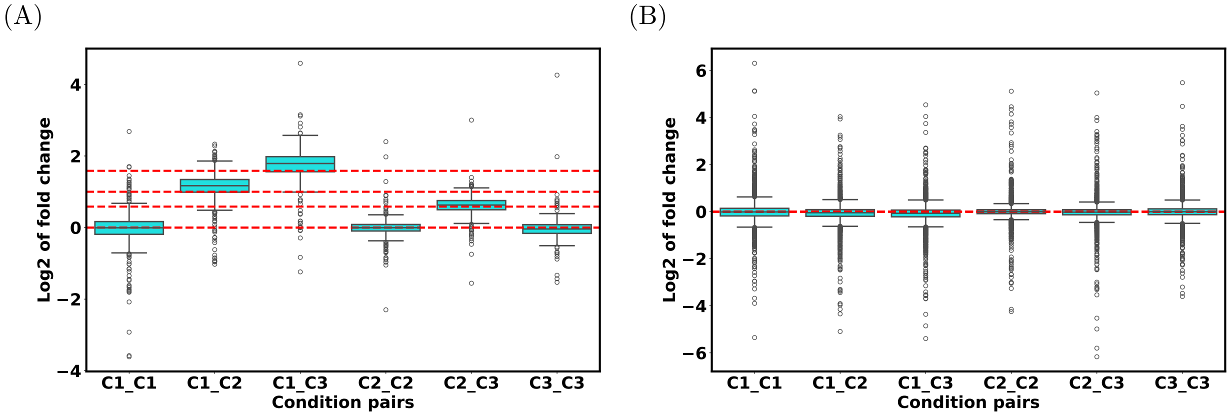

Figure S7: Species-level protein log fold changes for Dataset 2 obtained using MiCId. Box plots of distributions of average log fold changes are shown for (A) Yeast, and (B) Human for Dataset 2 (PXD007683). The figures show overall good performance by MiCId.

Figure S8

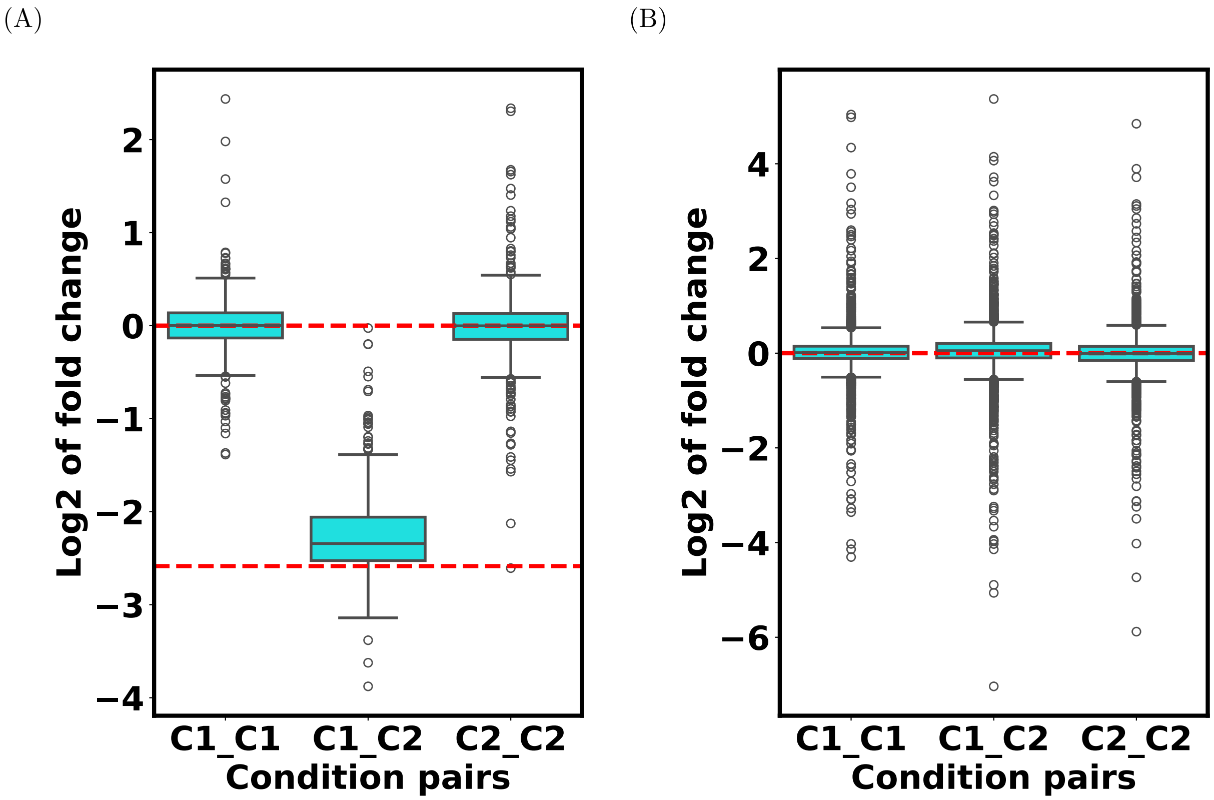

Figure S8: Species-level protein log fold changes for Dataset 3 obtained using MiCId. Box plots of distributions of average log fold changes are shown for (A) E.coli, and (B) Human for Dataset 3 (PXD006109). The figures show overall good performance by MiCId.

Figure S9

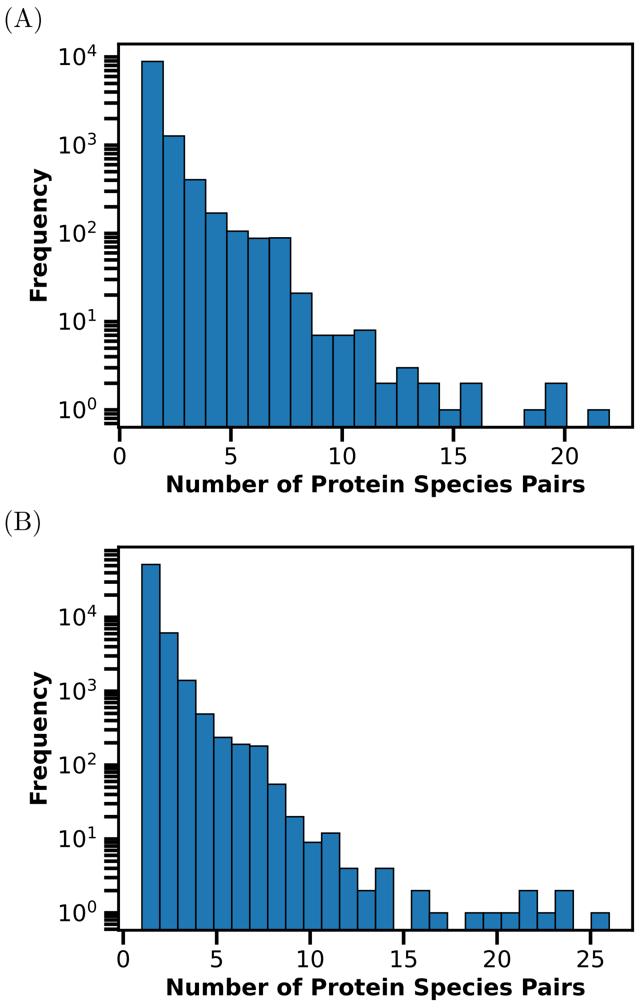

Figure S9: Distribution of the number of peptides shared among protein-species pairs. The figures show the distributions for (A) the mixture, and (B) the combined datasets.

Figure S10

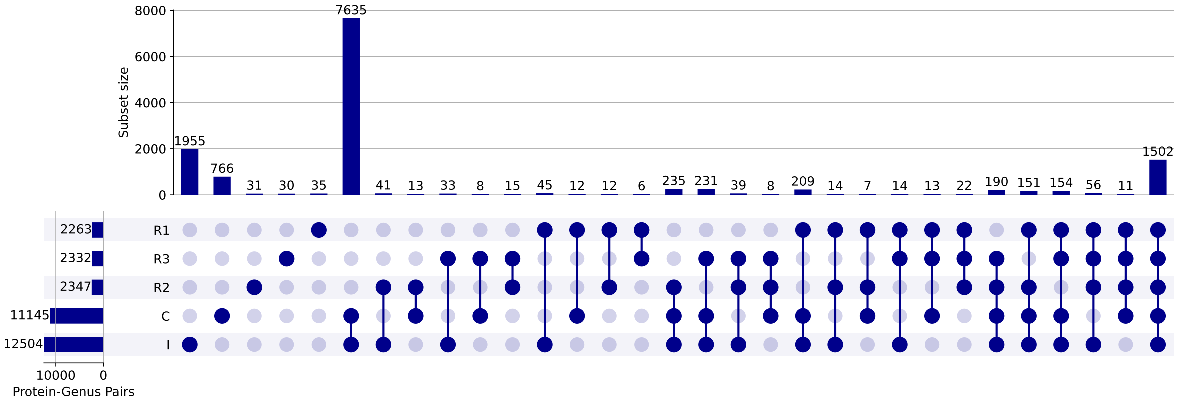

Figure S10: Assessing protein clustering and identification by MiCId at the genus level. A SetUp plot visualizing the overlap between the genus-protein pairs identified by MiCId when replicate 1 ($R1$), replicate 2 ($R2$), replicate 3 ($R3$), the combined dataset (C), and the individual (I) raw files are used as input to MiCId.

Figure S11

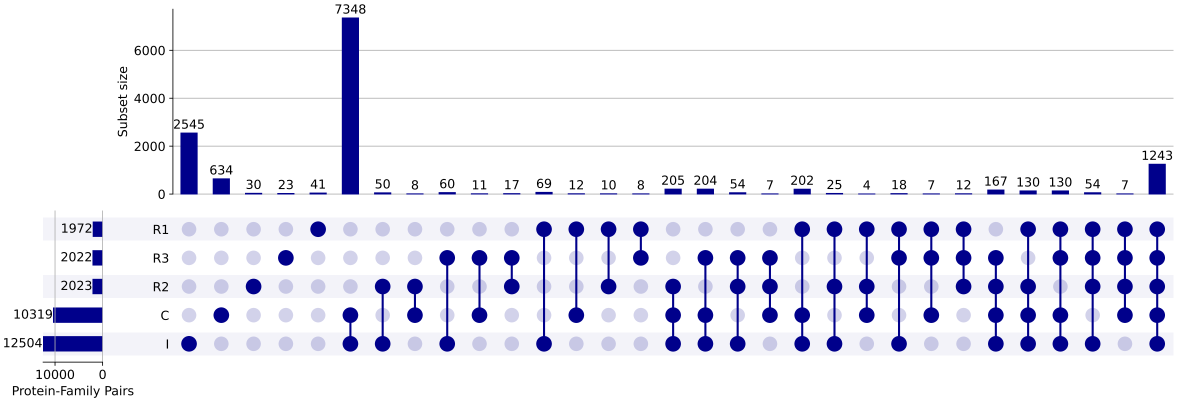

Figure S11: Assessing protein clustering and identification by MiCId at the family level. A SetUp plot visualizing the overlap between the family-protein pairs identified by MiCId when replicate 1 ($R1$), replicate 2 ($R2$), replicate 3 ($R3$), the combined dataset (C), and the individual (I) raw files are used as input to MiCId.

Figure S12

(A)

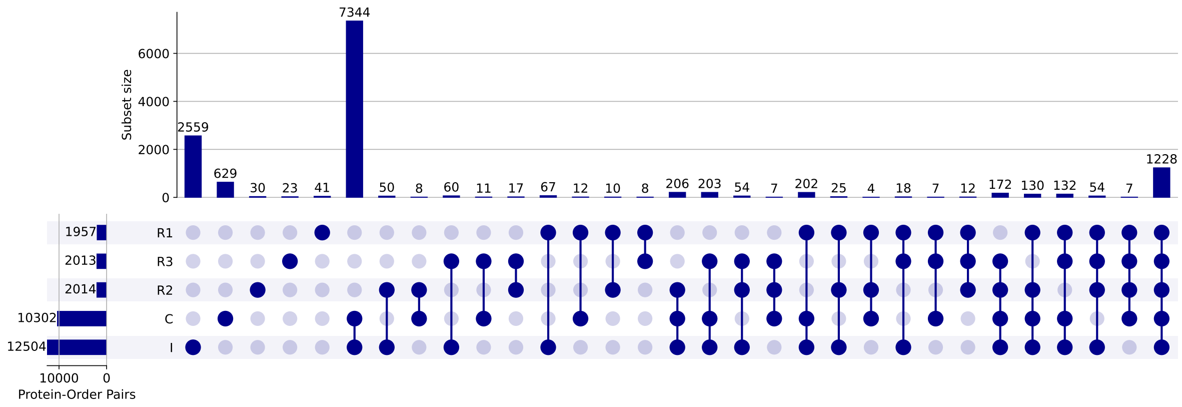

Figure S12: Assessing protein clustering and identification by MiCId at the order level. A SetUp plot visualizing the overlap between the order-protein pairs identified by MiCId when replicate 1 ($R1$), replicate 2 ($R2$), replicate 3 ($R3$), the combined dataset (C), and the individual (I) raw files are used as input to MiCId.

Figure S13

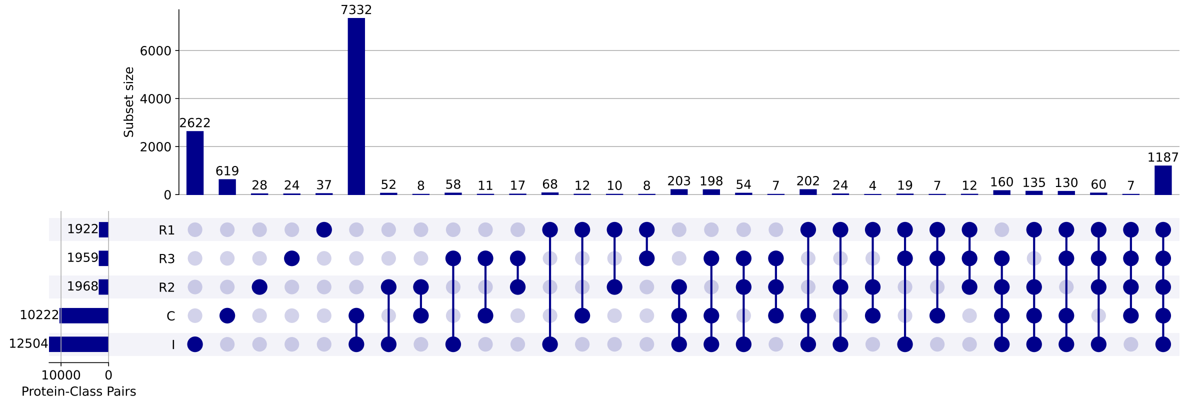

Figure S13: Assessing protein clustering and identification by MiCId at the class level. A SetUp plot visualizing the overlap between the class-protein pairs identified by MiCId when replicate 1 ($R1$), replicate 2 ($R2$), replicate 3 ($R3$), the combined dataset (C), and the individual (I) raw files are used as input to MiCId.

Figure S14

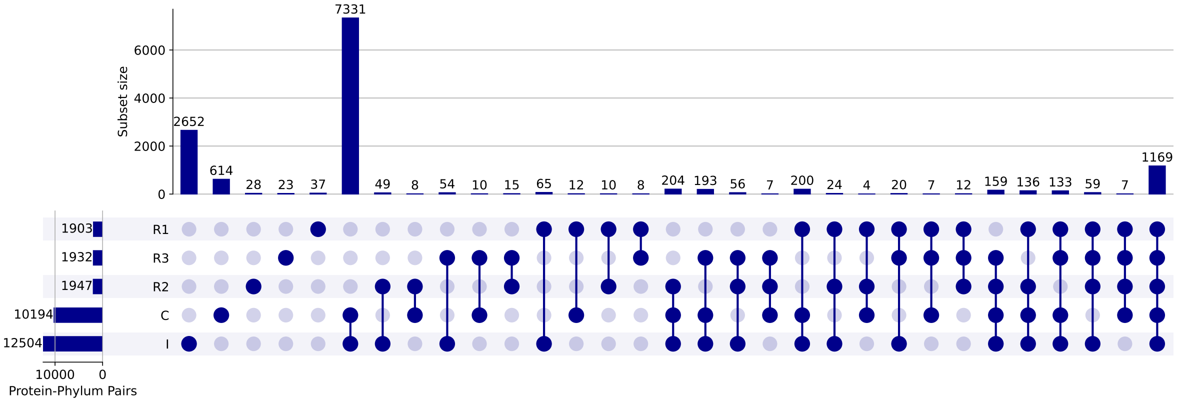

Figure S14: Assessing protein clustering and identification by MiCId at the phylum level. A SetUp plot visualizing the overlap between the phylum-protein pairs identified by MiCId when replicate 1 ($R1$), replicate 2 ($R2$), replicate 3 ($R3$), the combined dataset (C), and the individual (I) raw files are used as input to MiCId.

Figure S15

(A)

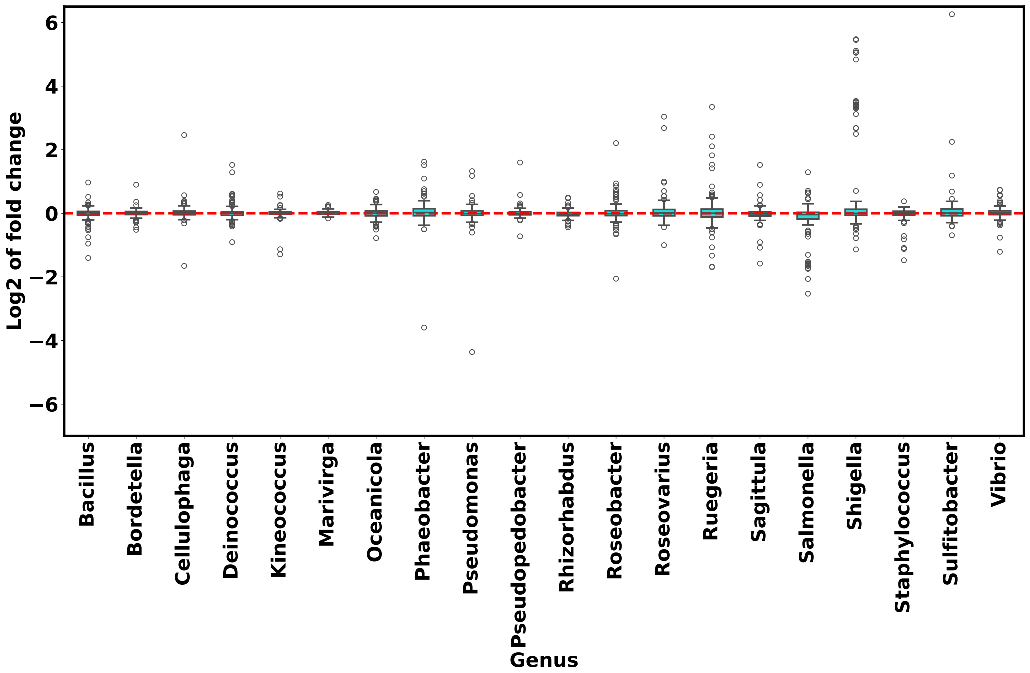

(B)

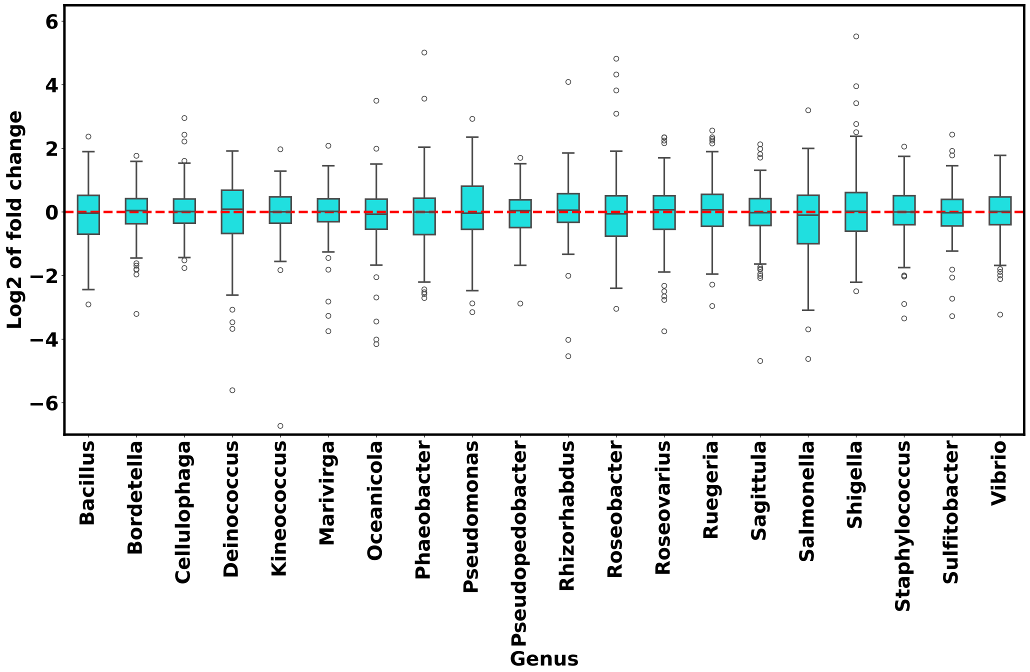

Figure S15: Average log fold changes at the genus level for Dataset 4 (PXD005776, PXD005728). Box plots show the distributions of mean log fold changes when comparing (A) the three replicate mixtures, and (B) the mixture vs. species-specific experiments.

Figure S16

(A)

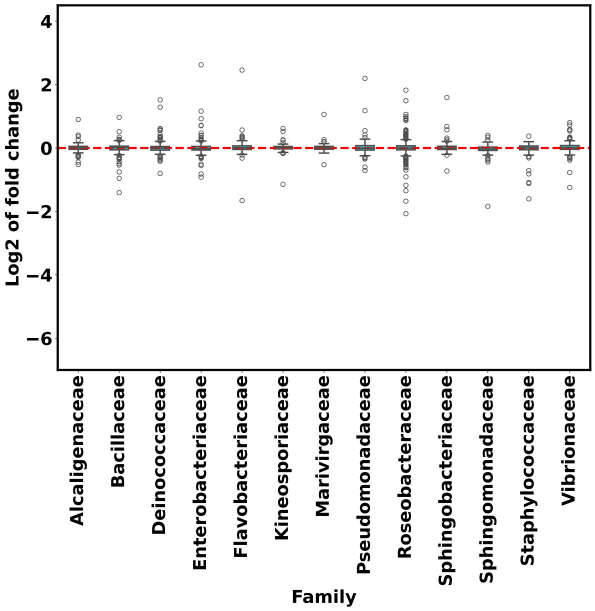

(B)

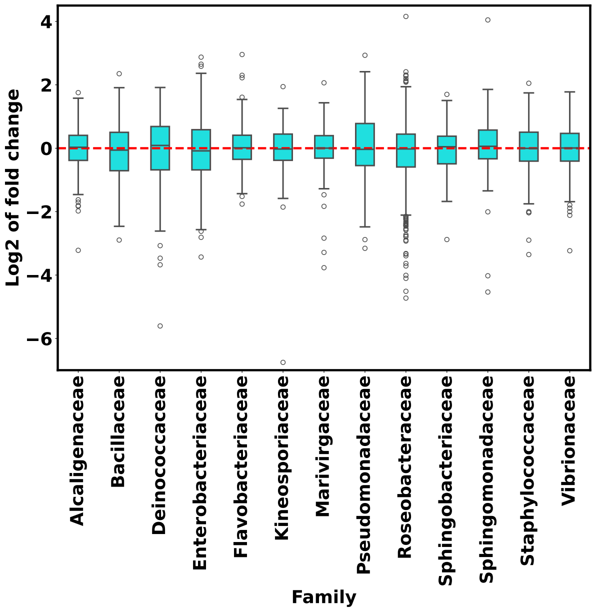

Figure S16: Average log fold changes at the family level for Dataset 4 (PXD005776, PXD005728). Box plots show the distributions of mean log fold changes when comparing (A) the three replicate mixtures, and (B) the mixture vs. species-specific experiments.

Figure S17

(A)

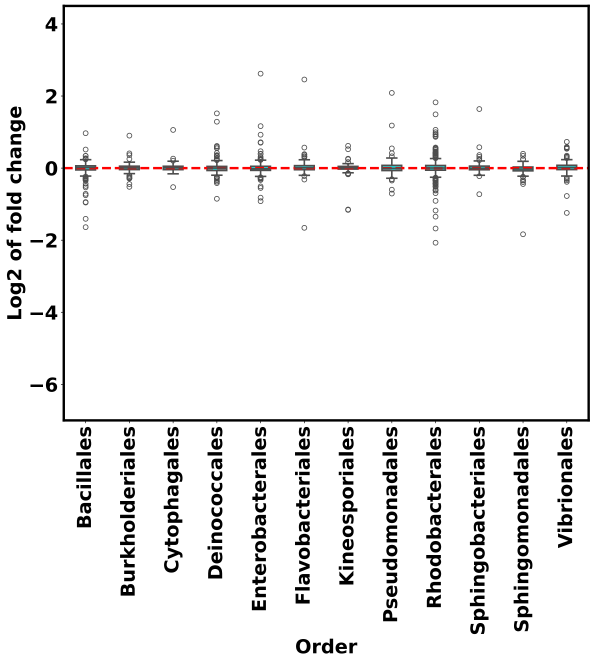

(B)

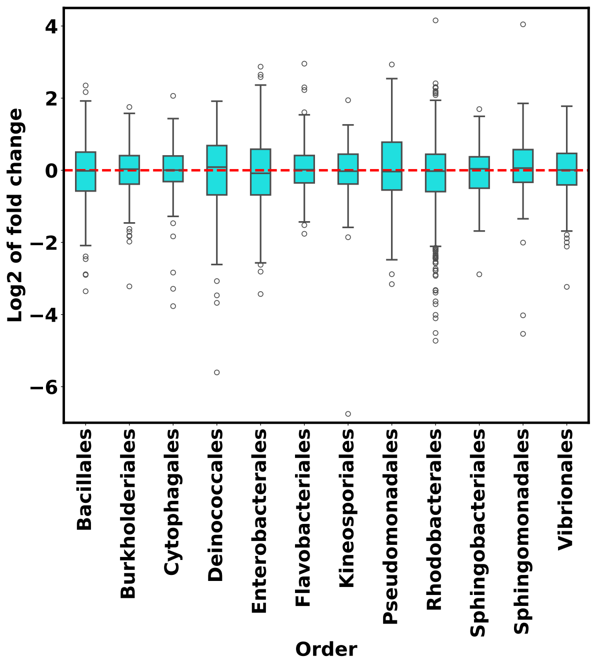

Figure S17: Average log fold changes at the order level for Dataset 4 (PXD005776, PXD005728). Box plots show the distributions of mean log fold changes when comparing (A) the three replicate mixtures, and (B) the mixture vs. species-specific experiments.

Figure S18

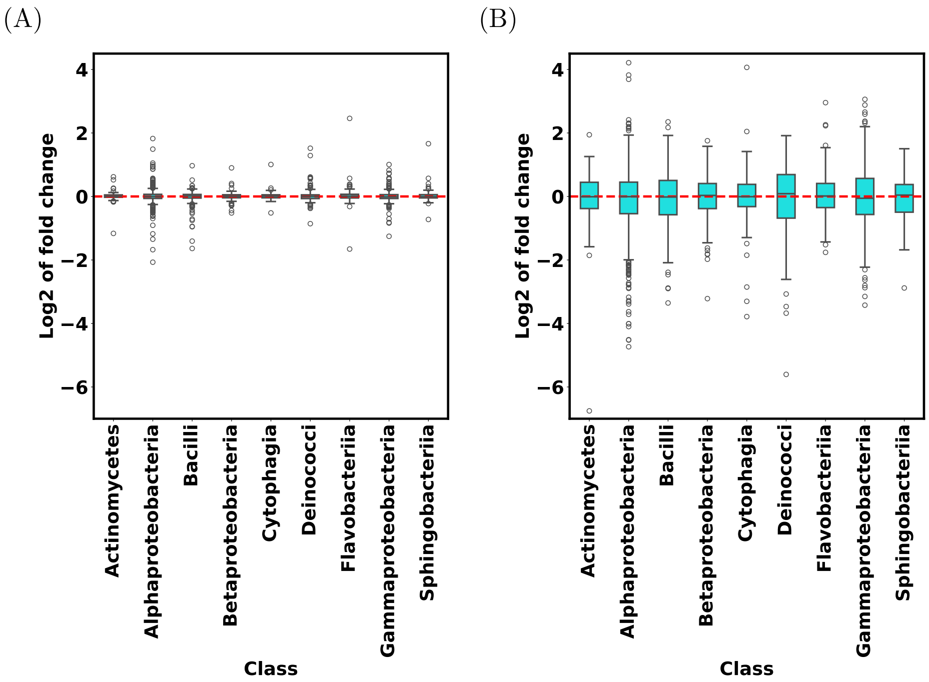

Figure S18: Average log fold changes at the class level for Dataset 4 (PXD005776, PXD005728). Box plots show the distributions of mean log fold changes when comparing (A) the three replicate mixtures, and (B) the mixture vs. species-specific experiments.

Figure S19

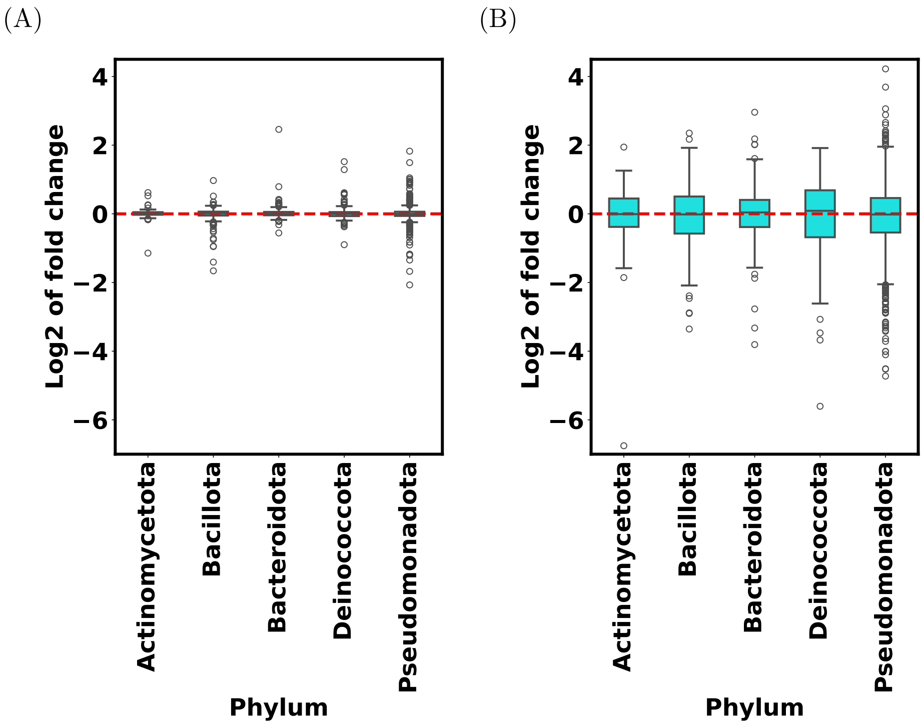

Figure S19: Average log fold changes at the phylum level for Dataset 4 (PXD005776, PXD005728). Box plots show the distributions of mean log fold changes when comparing (A) the three replicate mixtures, and (B) the mixture vs. species-specific experiments.

Figure S20

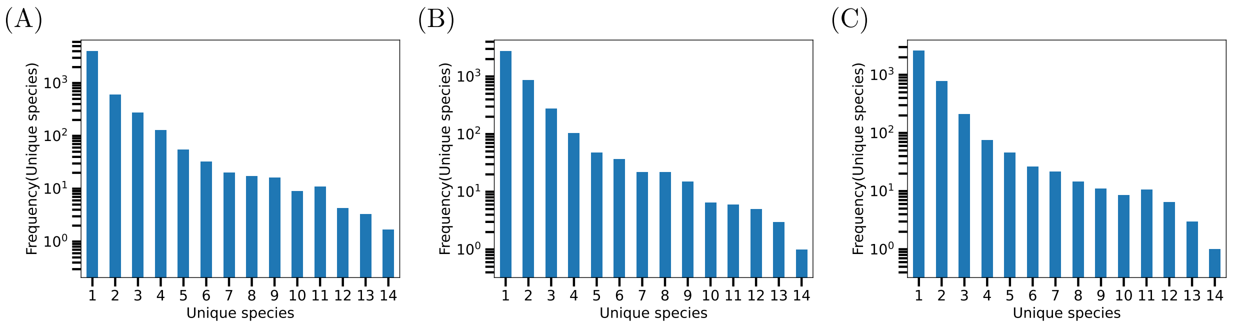

Figure S20: Frequency of unique species. The distributions of the number of unique species in protein clusters are plotted for (A) HM541, (B) HM604, and (C) HM609. The numbers are averaged over the replicates.

Figure S21

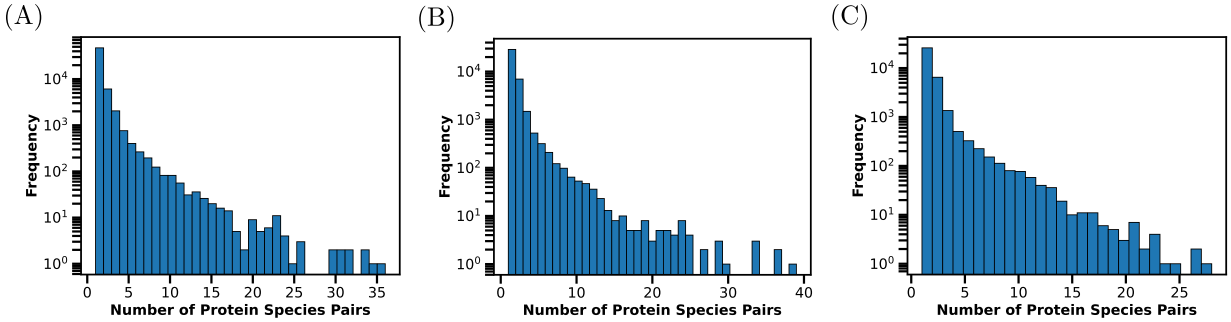

Figure S21: Distribution of the number protein-species pairs sharing a peptide. The distributions are shown for (A) HM541, (B) HM604, and (C) HM609.

Figure S22

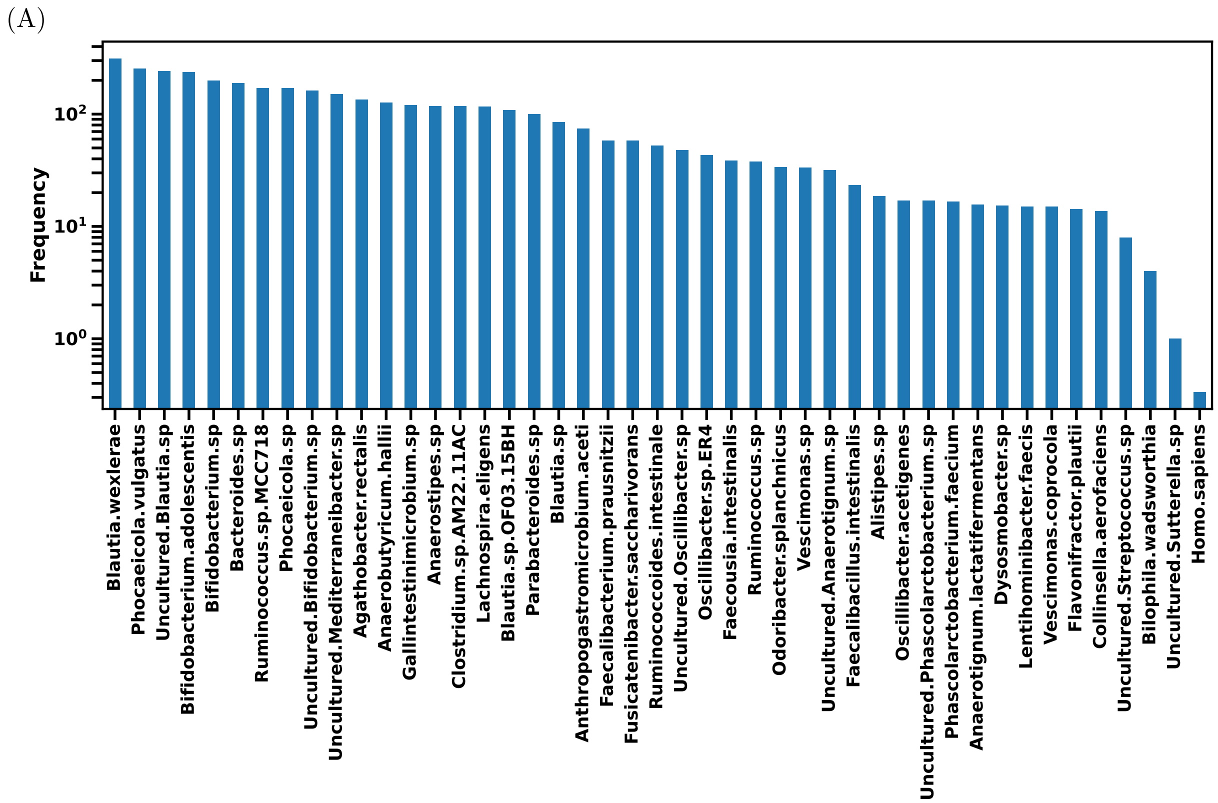

Figure S22: The number of proteins of a particular species that are in clusters containing other species. For sample HM541 the average number (frequency) of proteins is plotted for each species. The number is averaged over the replicates.

Figure S23

(A)

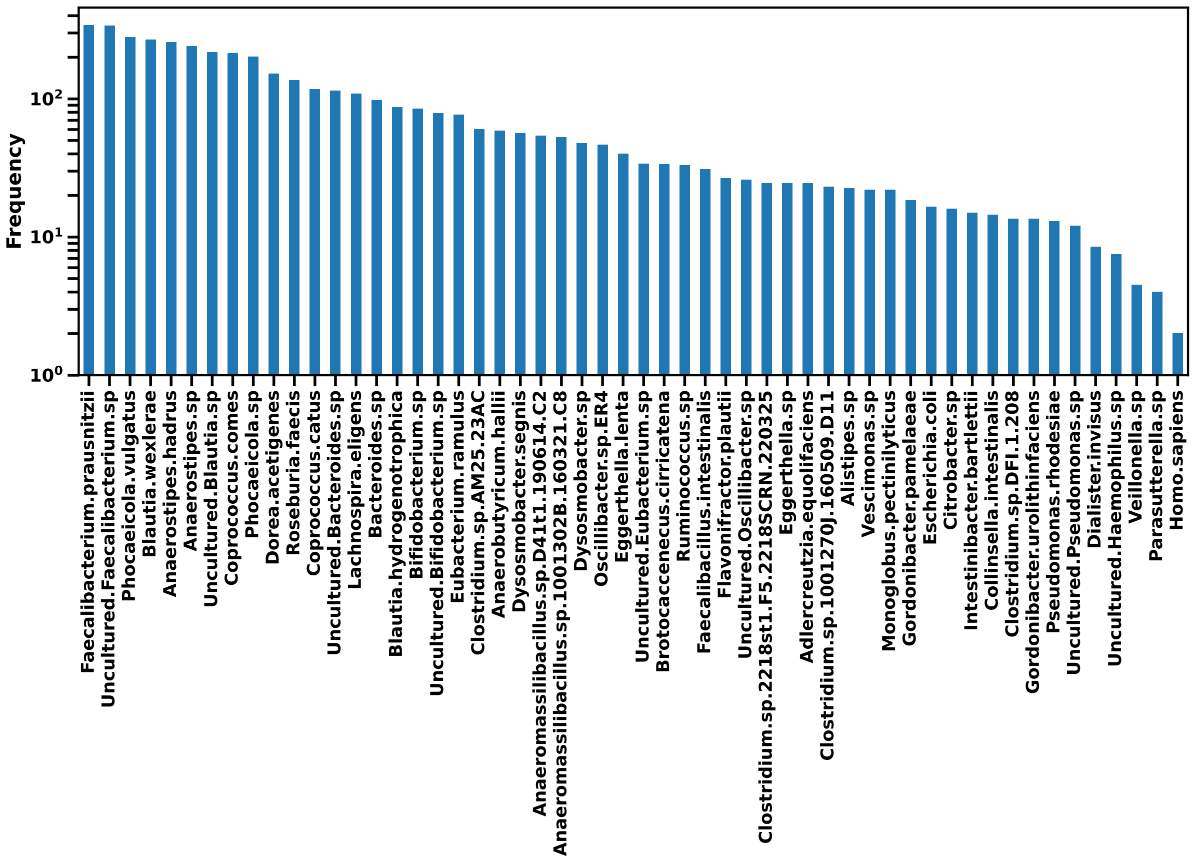

Figure S23: The number of proteins of a particular species that are in clusters containing other species. For sample HM604 the average number (frequency) of proteins is plotted for each species. The number is averaged over the replicates.

Figure S24

(A)

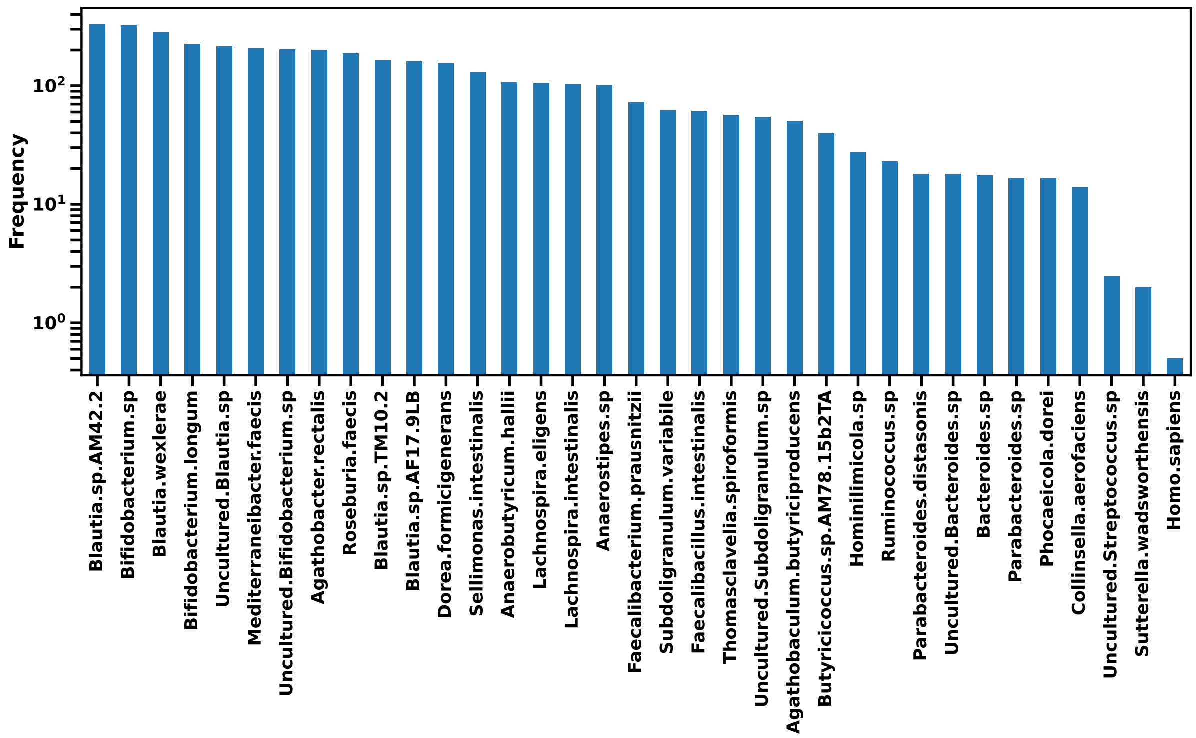

Figure S24: The number of proteins of a particular species that are in clusters containing other species. For sample HM609 the average number (frequency) of proteins is plotted for each species. The number is averaged over the replicates.

Figure S25

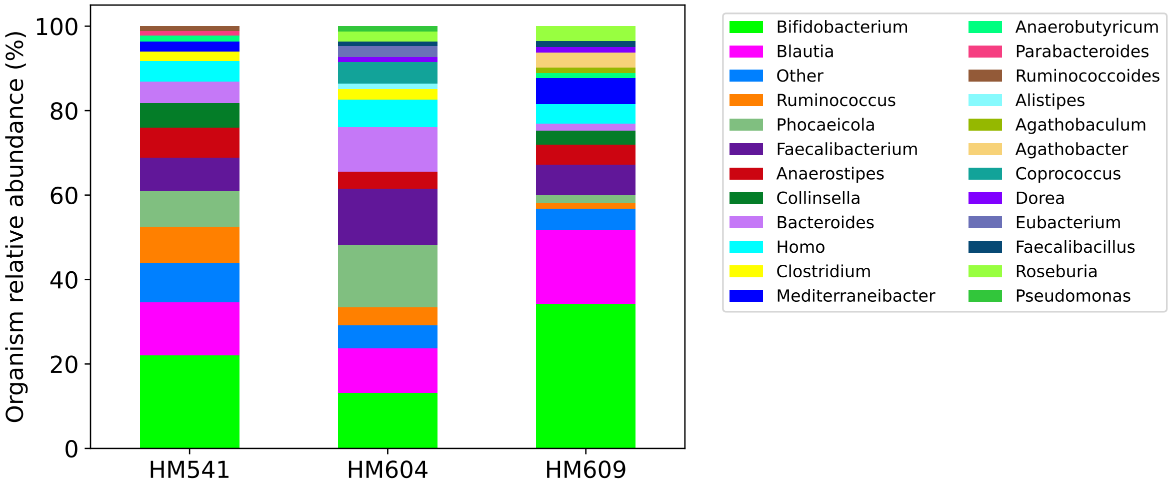

Figure S25: Genus-level biomass plot. Relative abundances (larger than 0.01) at the genus level are shown for the three samples.

Figure S26

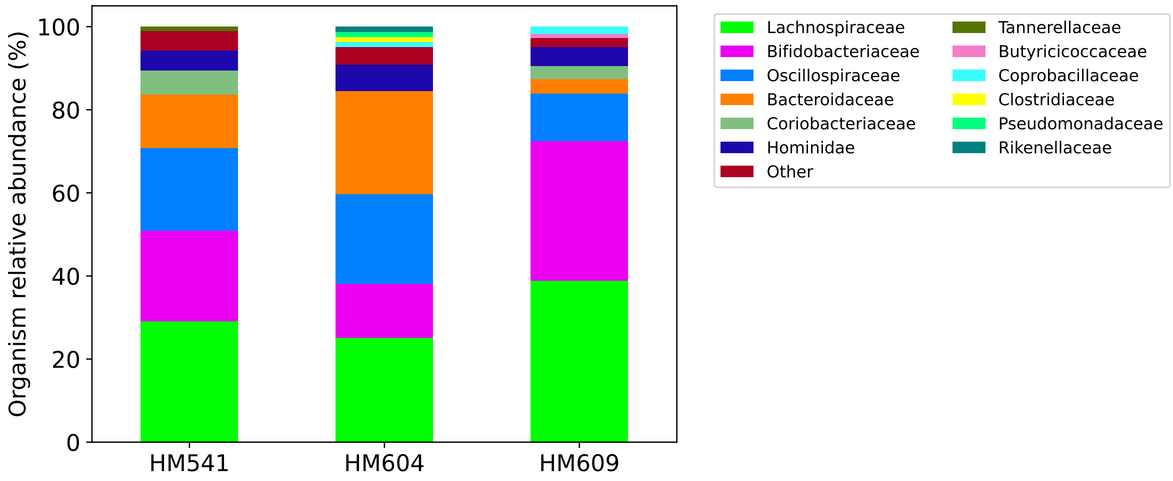

Figure S26: Family-level biomass plot. Relative abundances (larger than 0.01) at the family level are shown for the three samples.

Figure S27

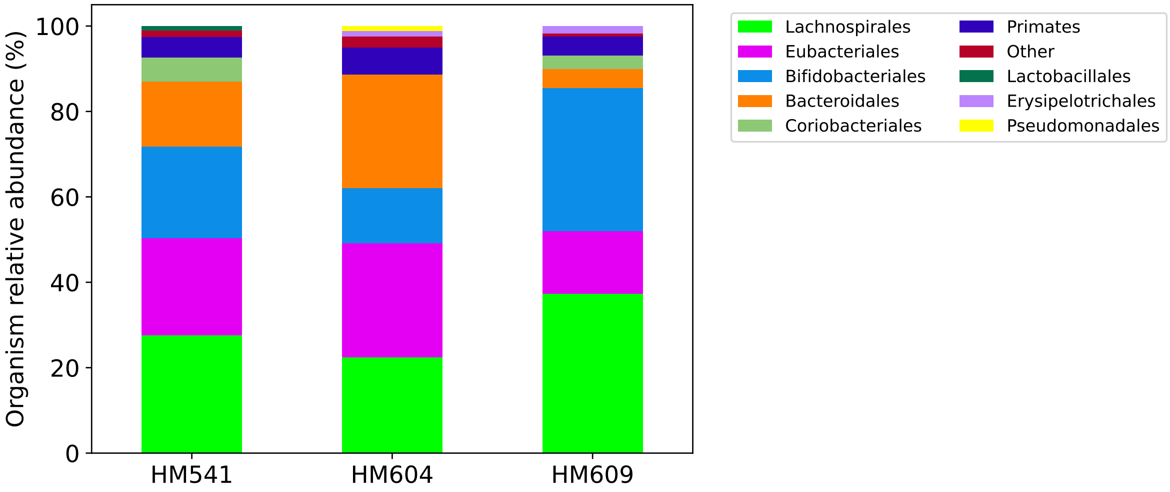

Figure S27: Order-level biomass plot. Relative abundances (larger than 0.01) at the order level are shown for the three samples.

Figure S28

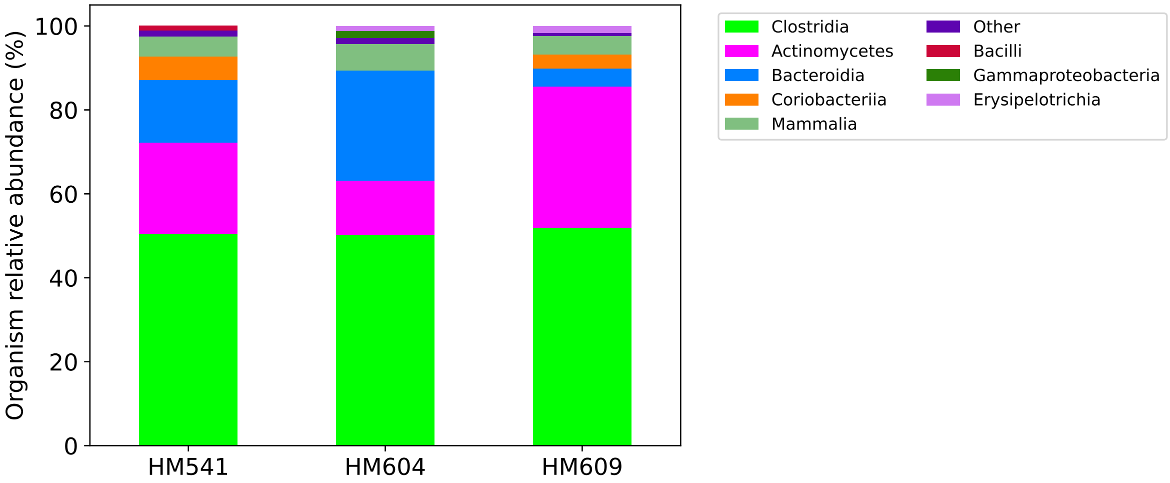

Figure S28: Class-level biomass plot. Relative abundances (larger than 0.01) at the class level are shown for the three samples.

Figure S29

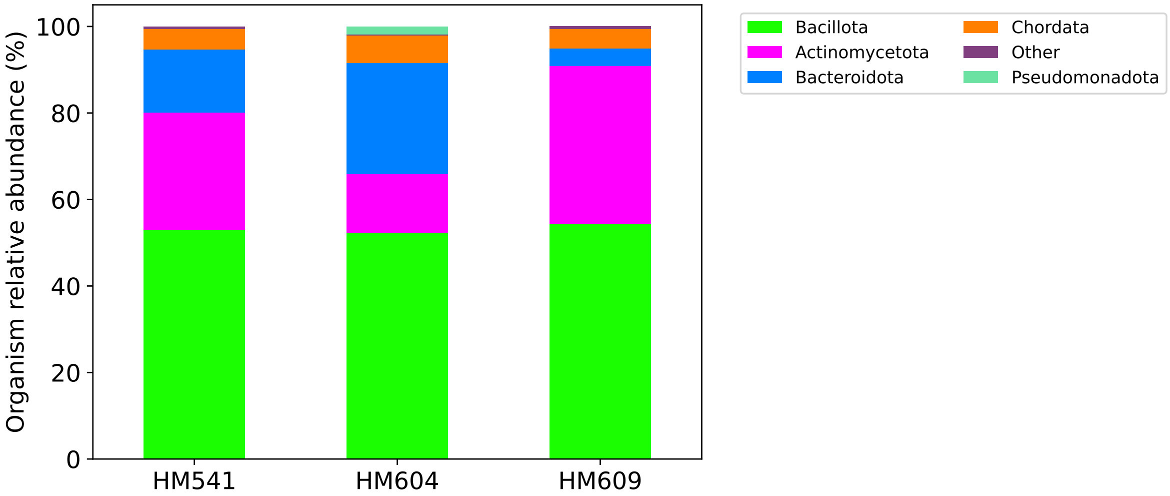

Figure S29: Phylum-level biomass plot. Relative abundances (larger than 0.01) at the phylum level are shown for the three samples.

Figure S30

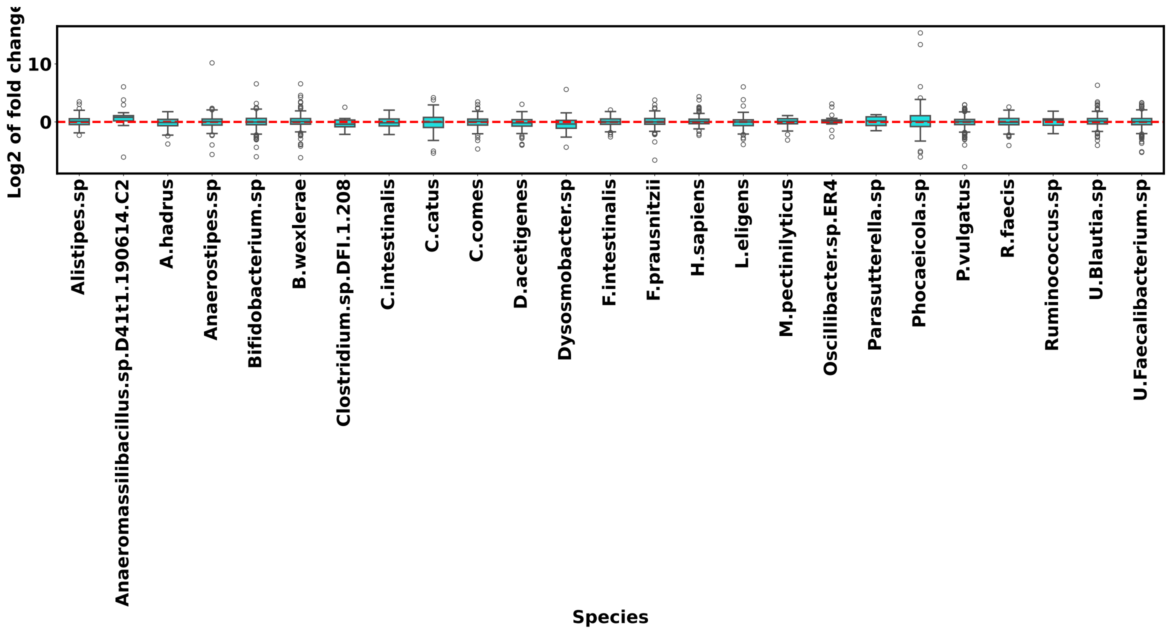

Figure S30: Distributions of log fold change for each species. For patient HM604, the distributions are shown for all species with more than 10 identified proteins. The fold changes were calculated between the technical replicates. For species that have not been yet named (the ones containing ".sp"), the genus name has been fully spelled. The "U." at the beginning of some of the names denotes "Uncultured".

Figure S31

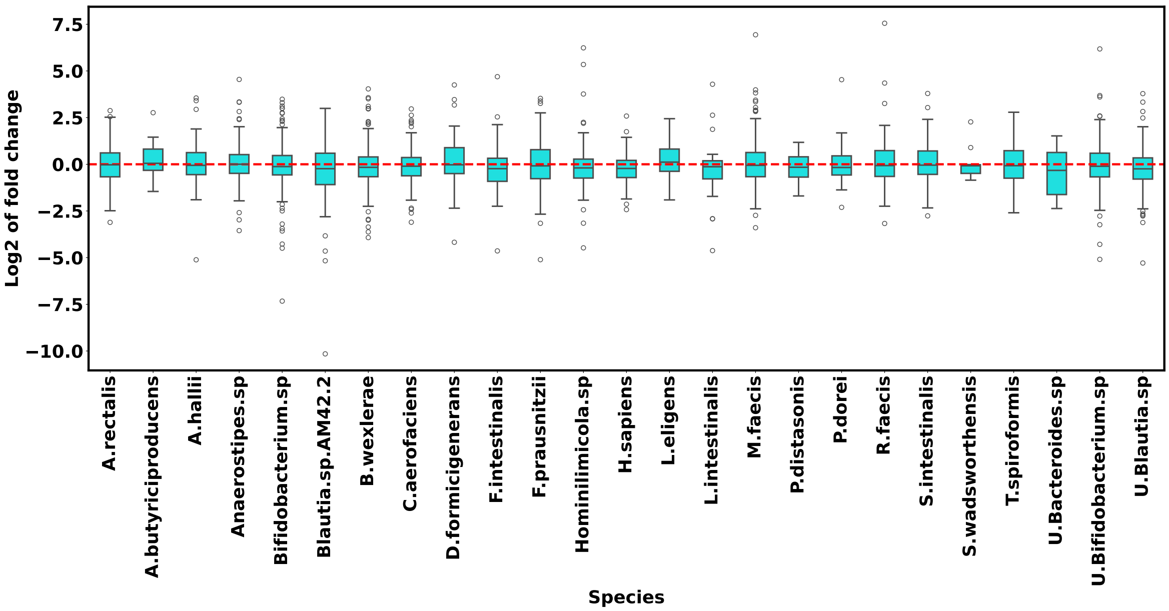

Figure S31: Distributions of log fold change for each species. For patient HM609, the distributions are shown for all species with more than 10 identified proteins. The fold changes were calculated between the technical replicates. For species that have not been yet named (the ones containing “.sp”), the genus name has been fully spelled. The “U.” at the beginning of some of the names denotes “Uncultured”.

Figure S32

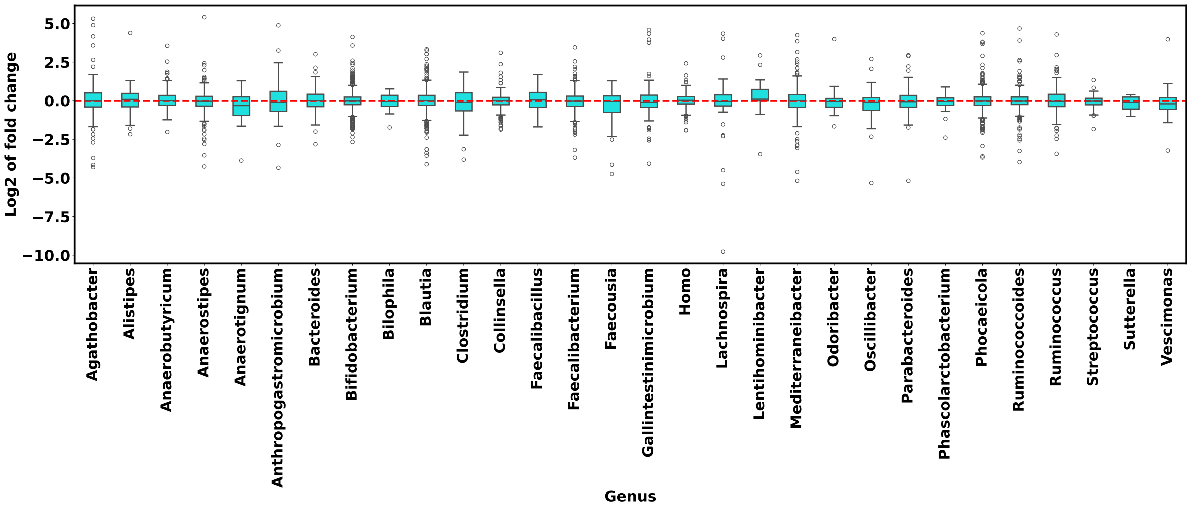

Figure S32: Distributions of log fold change for each genus. For patient HM541, the distributions are shown, at the genus level, if there were more than 10 identified proteins for the genus.

Figure S33

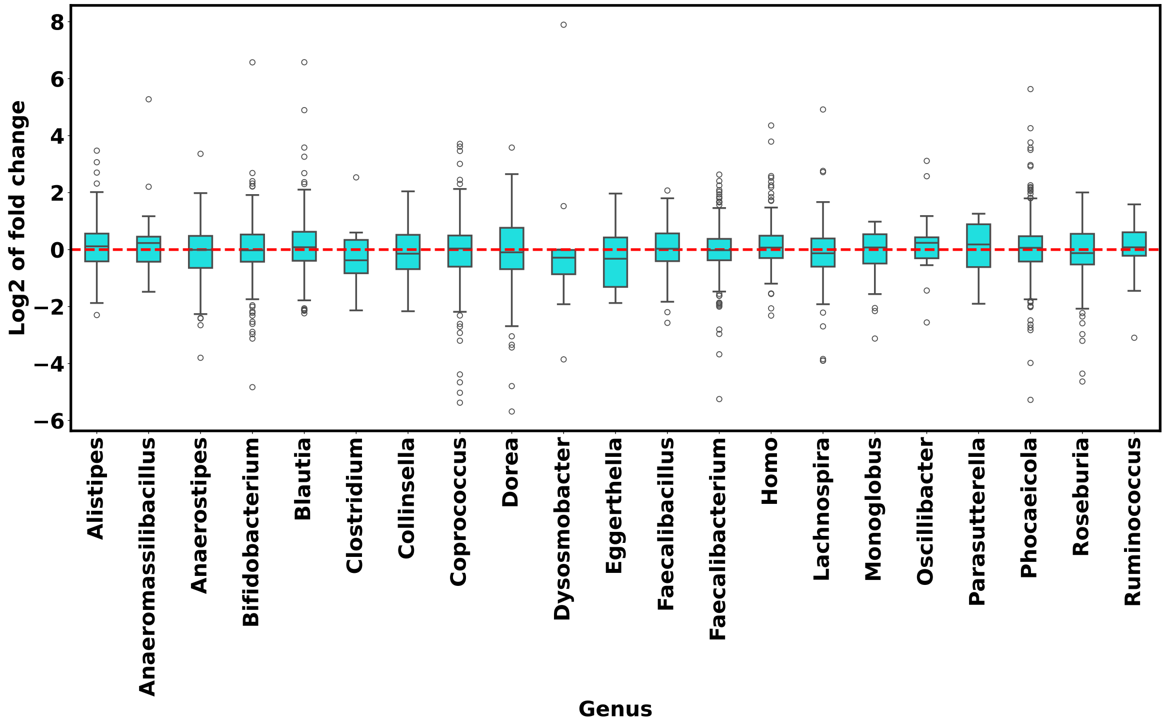

Figure S33: Distributions of log fold change for each genus. For patient HM604, the distributions are shown, at the genus level, if there were more than 10 identified proteins for the genus.

Figure S34

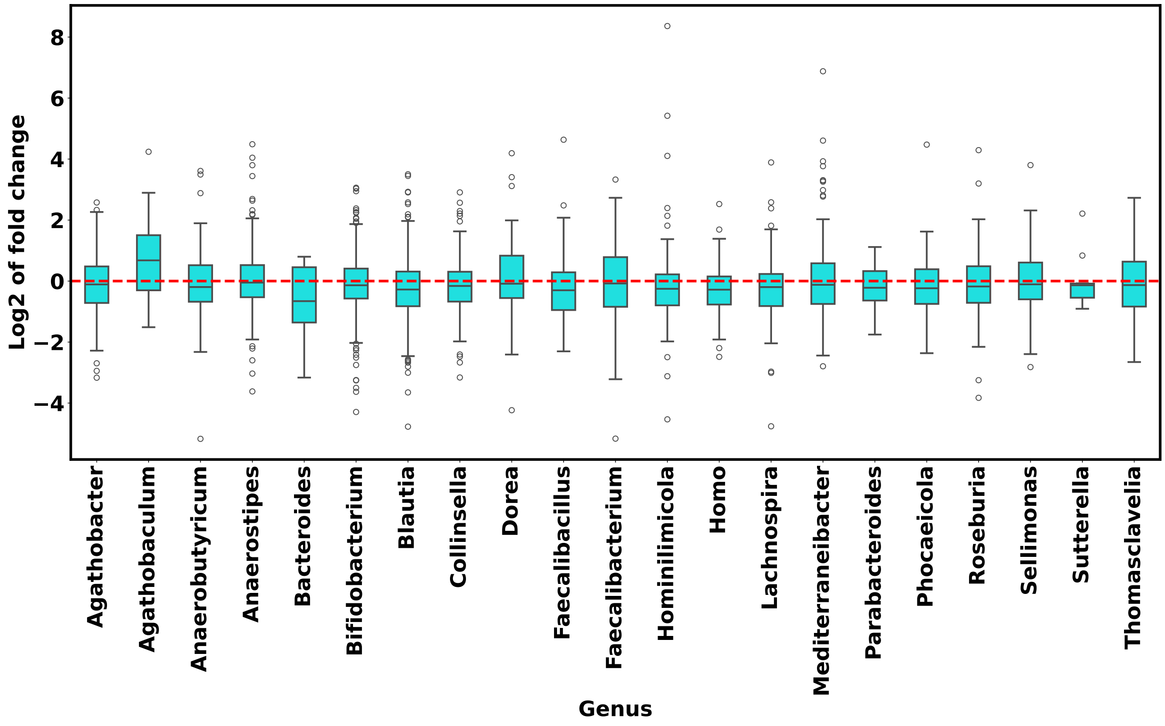

Figure S34: Distributions of log fold change for each genus. For patient HM609, the distributions are shown, at the genus level, if there were more than 10 identified proteins for the genus.

Figure S35

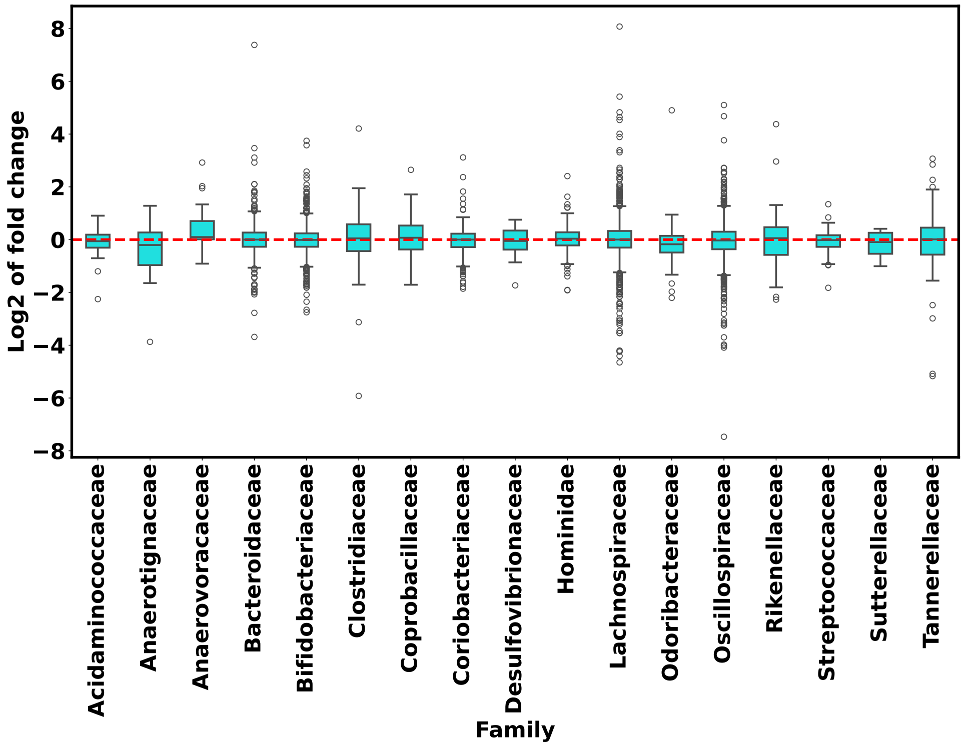

Figure S35: Distributions of log fold change for each family. For patient HM541, the distributions are shown, at the family level, if there were more than 10 identified proteins for the family.

Figure S36

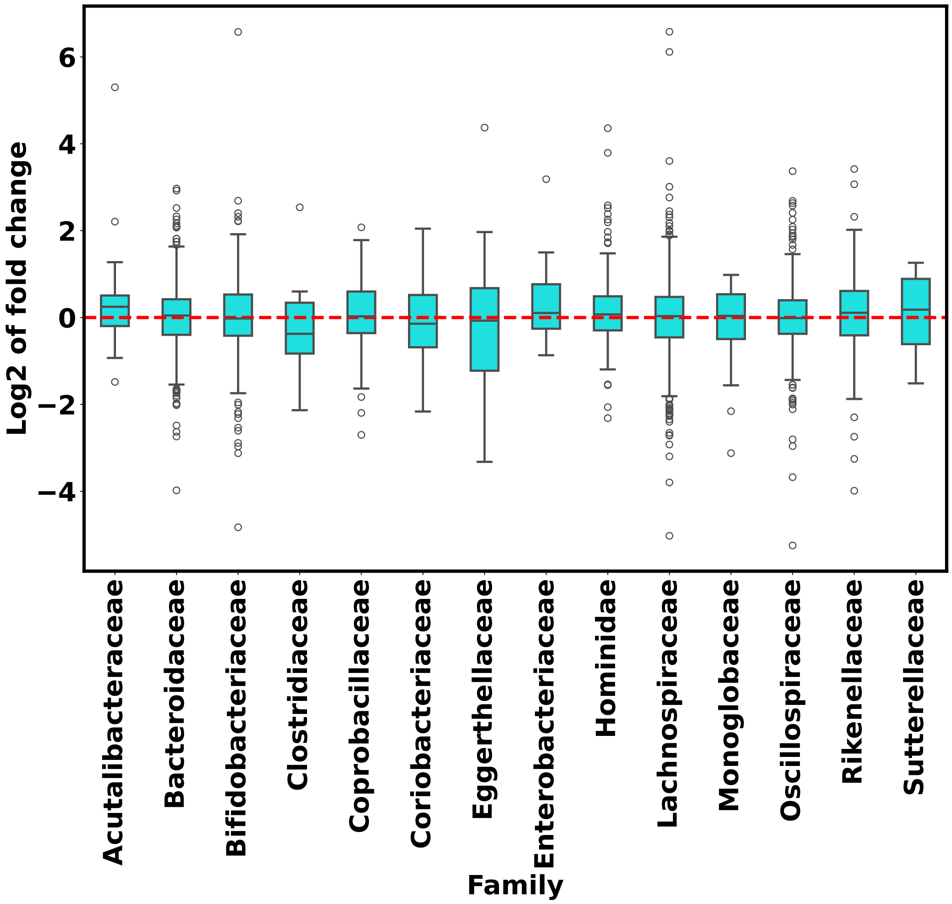

Figure S36: Distributions of log fold change for each family. For patient HM604, the distributions are shown, at the family level, if there were more than 10 identified proteins for the family.

Figure S37

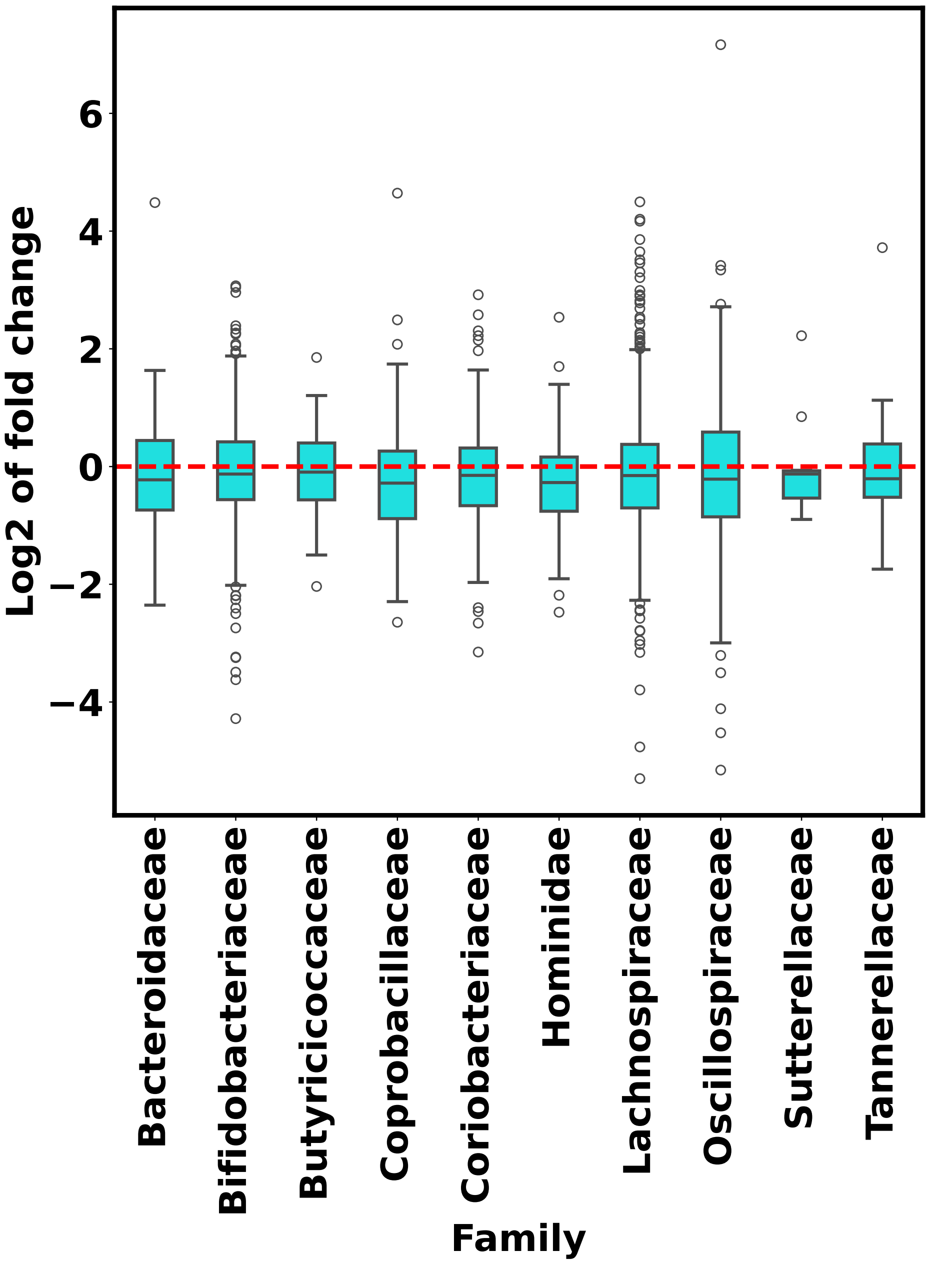

Figure S37: Distributions of log fold change for each family. For patient HM609, the distributions are shown, at the family level, if there were more than 10 identified proteins for the family.

Figure S38

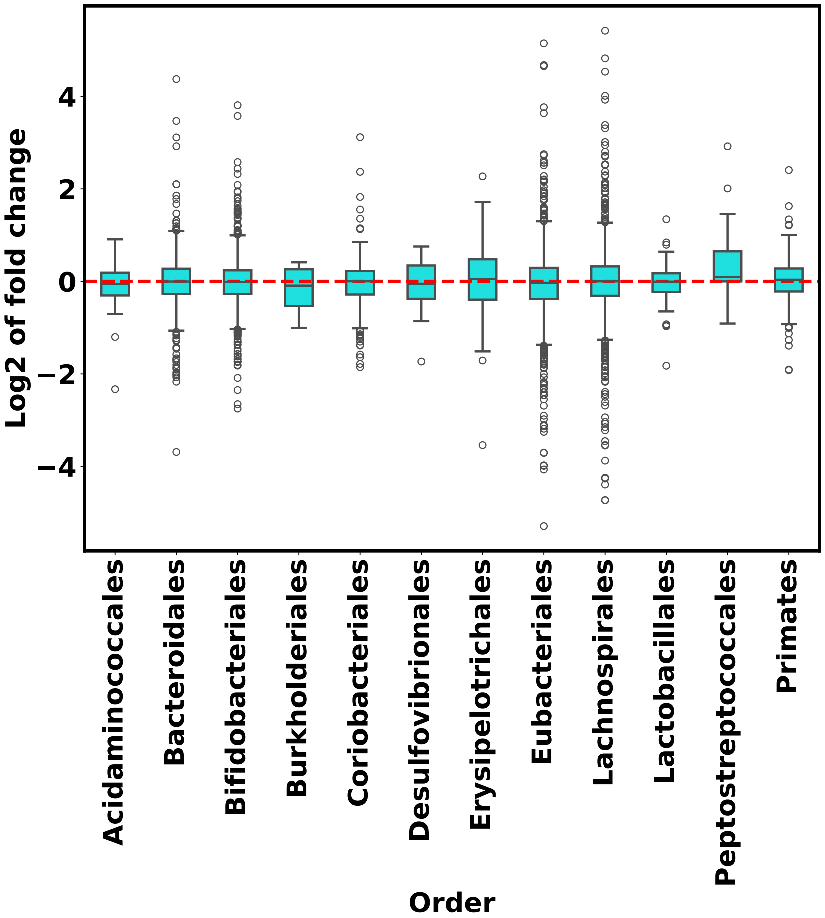

Figure S38: Distributions of log fold change for each order. For patient HM541, the distributions are shown, at the order level, if there were more than 10 identified proteins for the order.

Figure S39

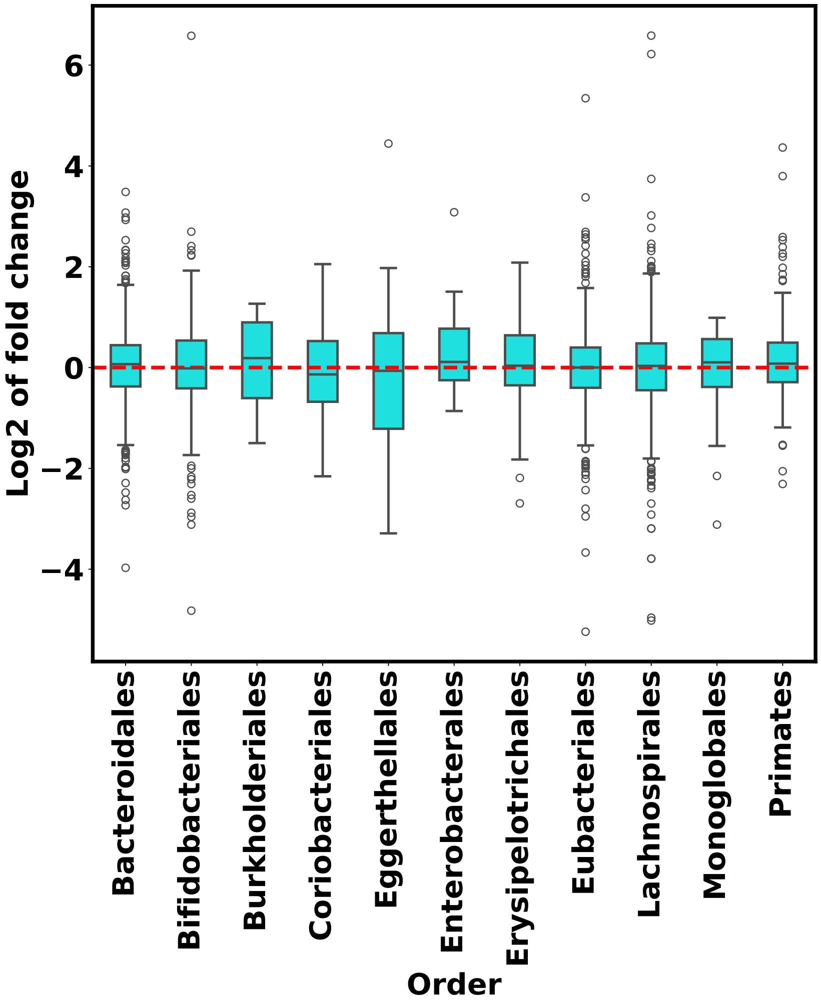

Figure S39: Distributions of log fold change for each order. For patient HM604, the distributions are shown, at the order level, if there were more than 10 identified proteins for the order.

Figure S40

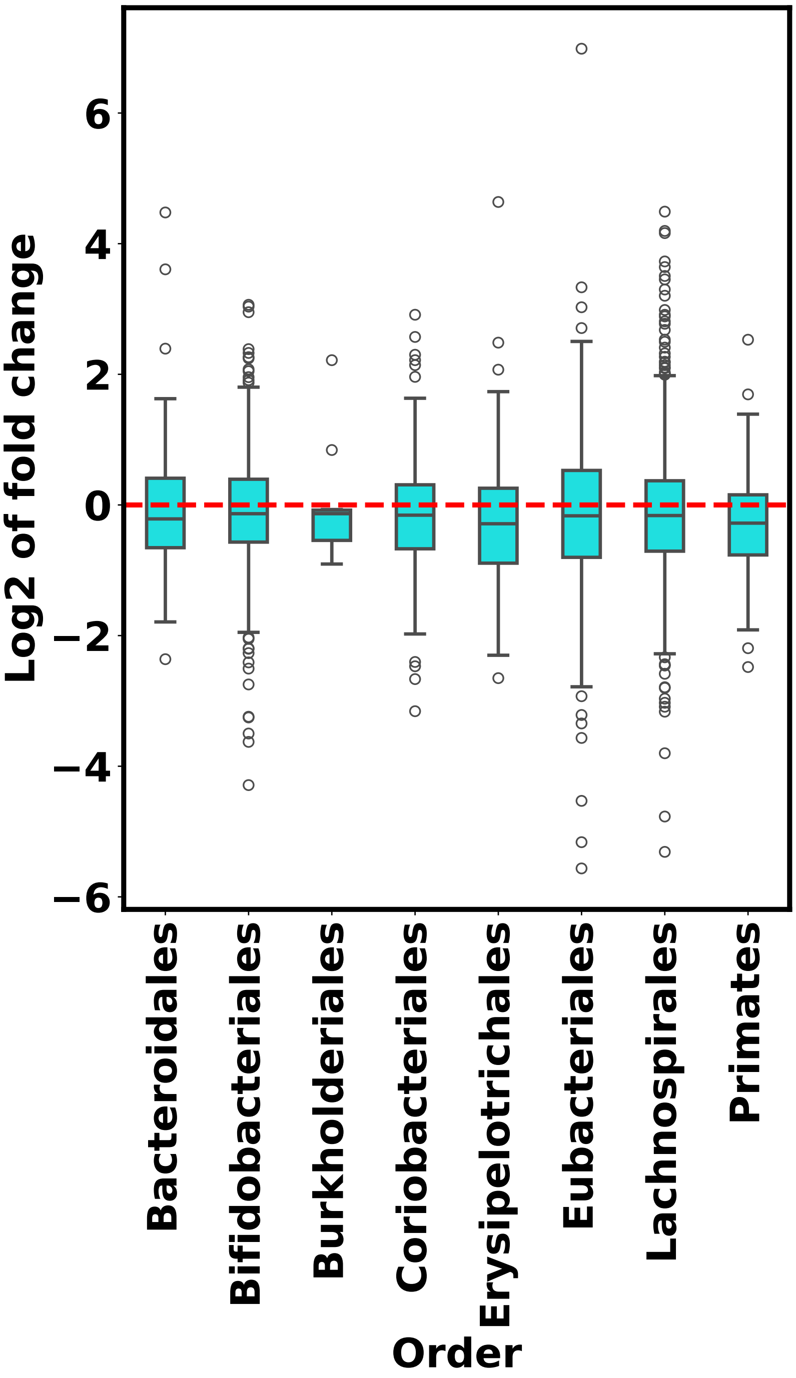

Figure S40: Distributions of log fold change for each order. For patient HM609, the distributions are shown, at the order level, if there were more than 10 identified proteins for the order.

Figure S41

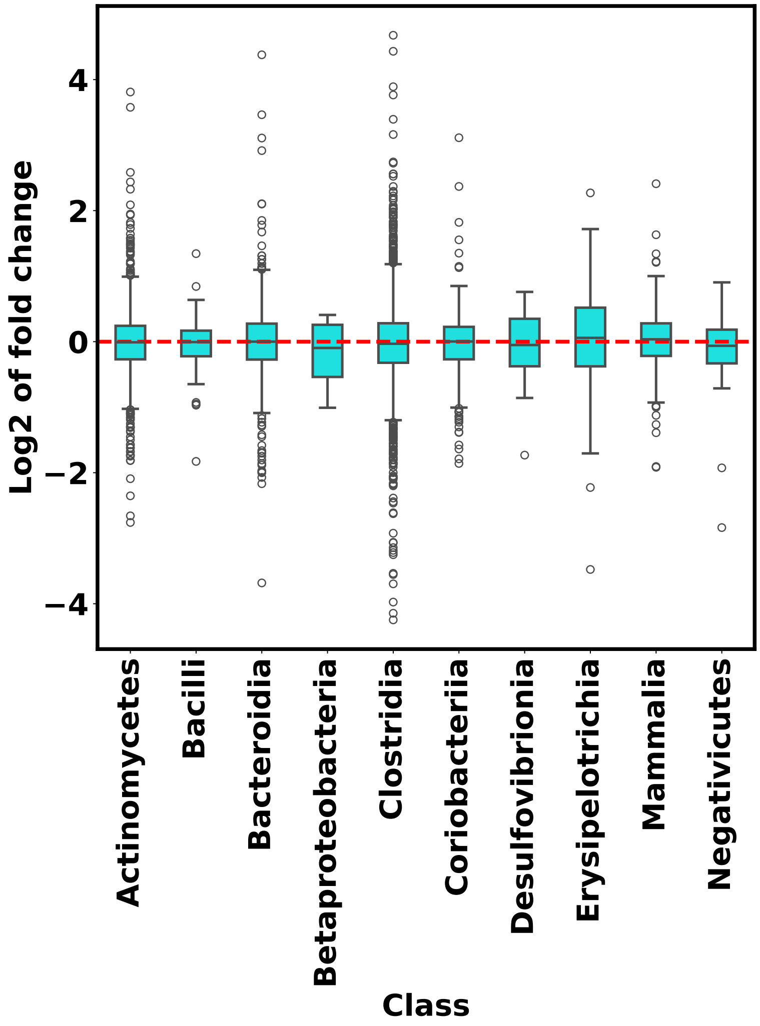

Figure S41: Distributions of log fold change for each class. For patient HM541, the distributions are shown, at the class level, if there were more than 10 identified proteins for the class.

Figure S42

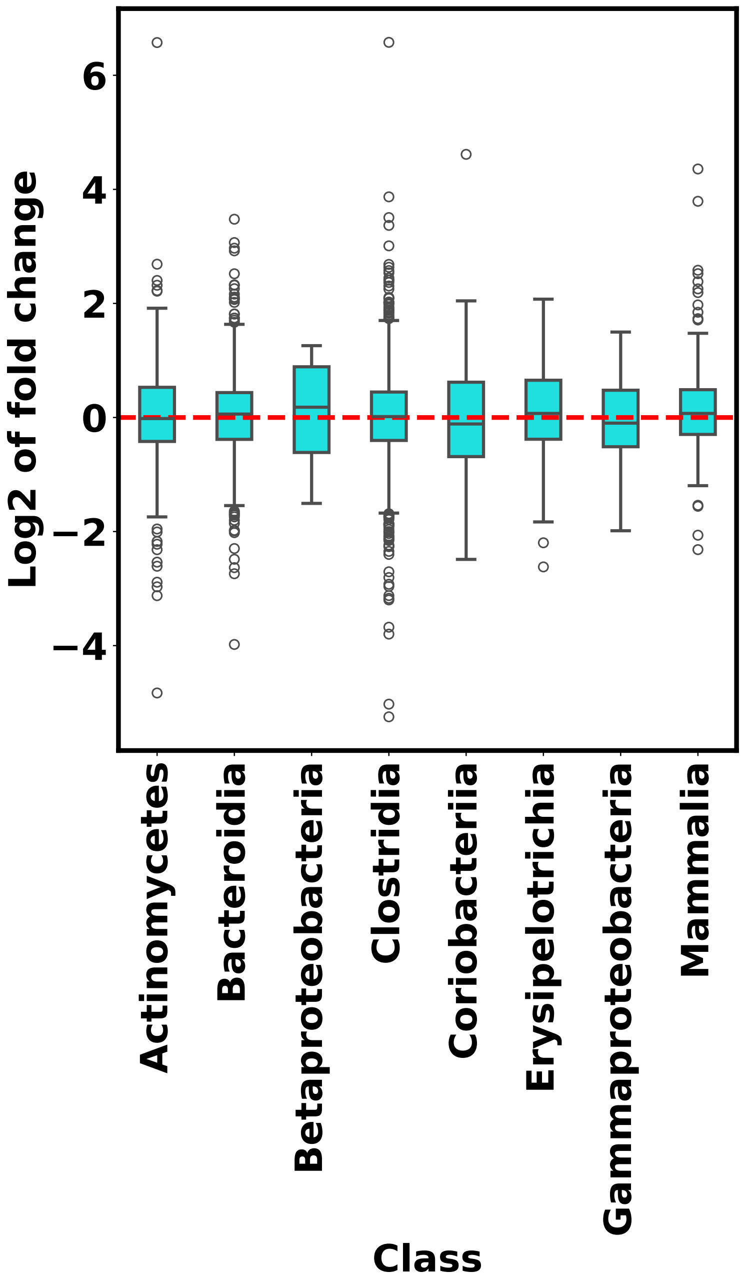

Figure S42: Distributions of log fold change for each class. For patient HM604, the distributions are shown, at the class level, if there were more than 10 identified proteins for the class.

Figure S43

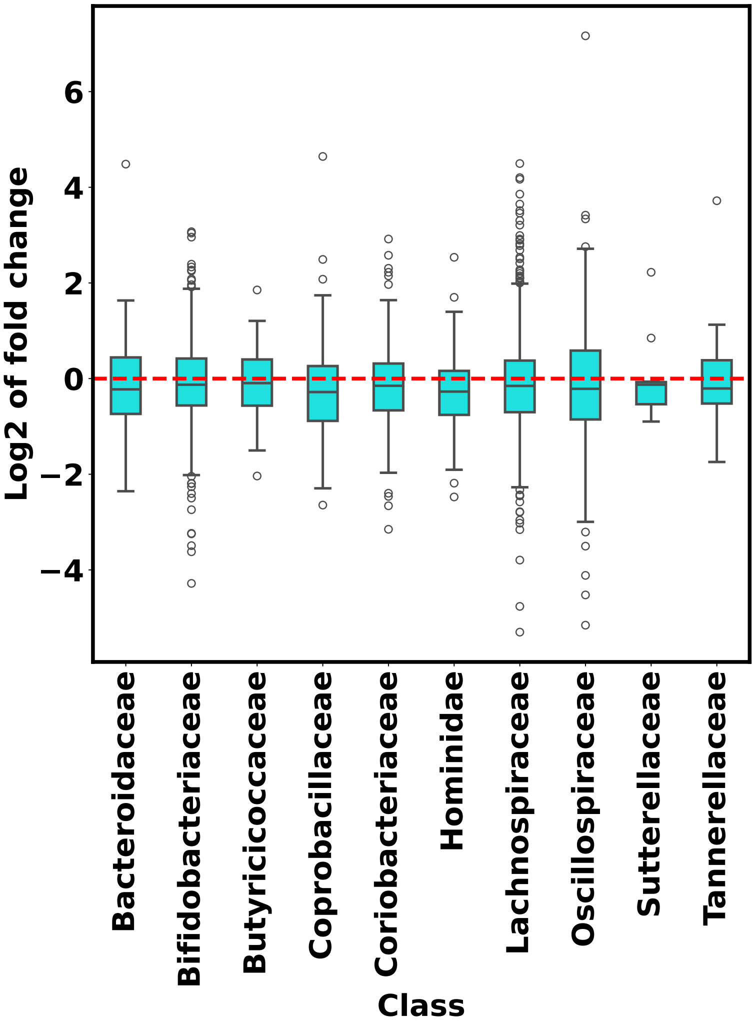

Figure S43: Distributions of log fold change for each class. For patient HM609, the distributions are shown, at the class level, if there were more than 10 identified proteins for the class.

Figure S44

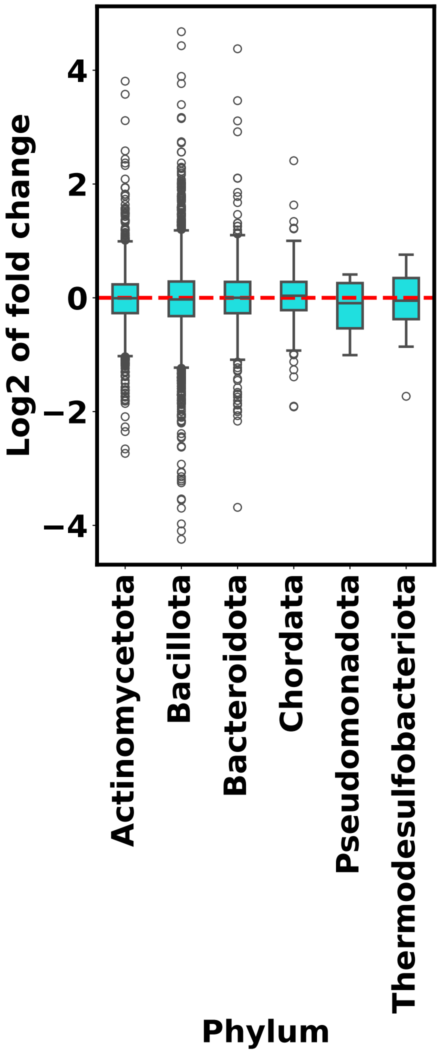

Figure S44: Distributions of log fold change for each phylum. For patient HM541, the distributions are shown, at the phylum level, if there were more than 10 identified proteins for the phylum.

Figure S45

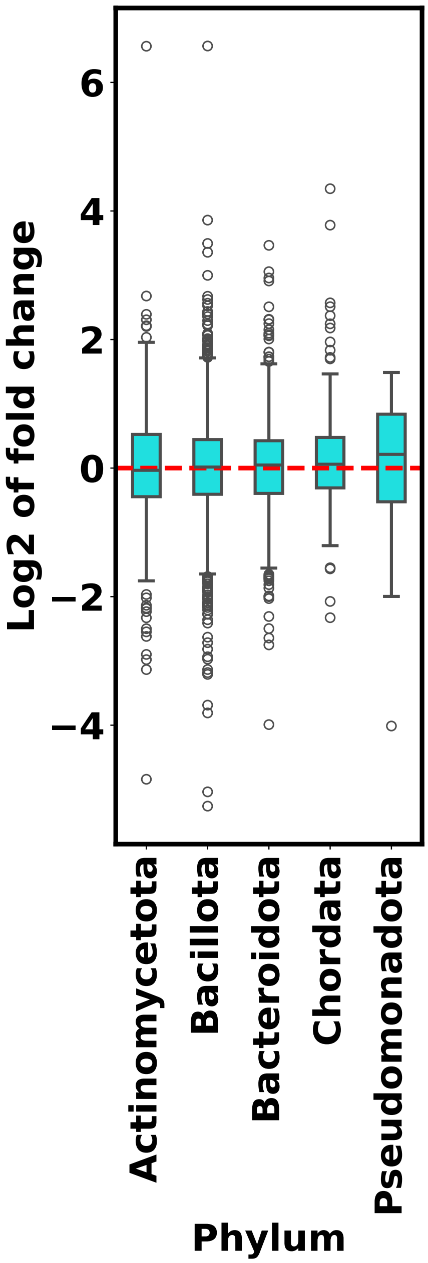

Figure S45: Distributions of log fold change for each phylum. For patient HM604, the distributions are shown, at the phylum level, if there were more than 10 identified proteins for the phylum.

Figure S46

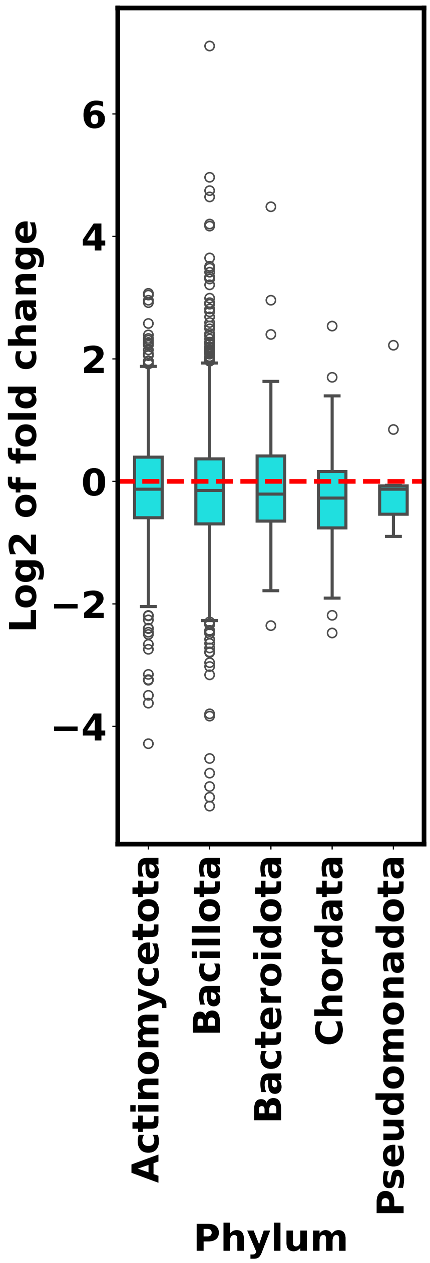

Figure S46: Distributions of log fold change for each phylum. For patient HM609, the distributions are shown, at the phylum level, if there were more than 10 identified proteins for the phylum.

Figure S47

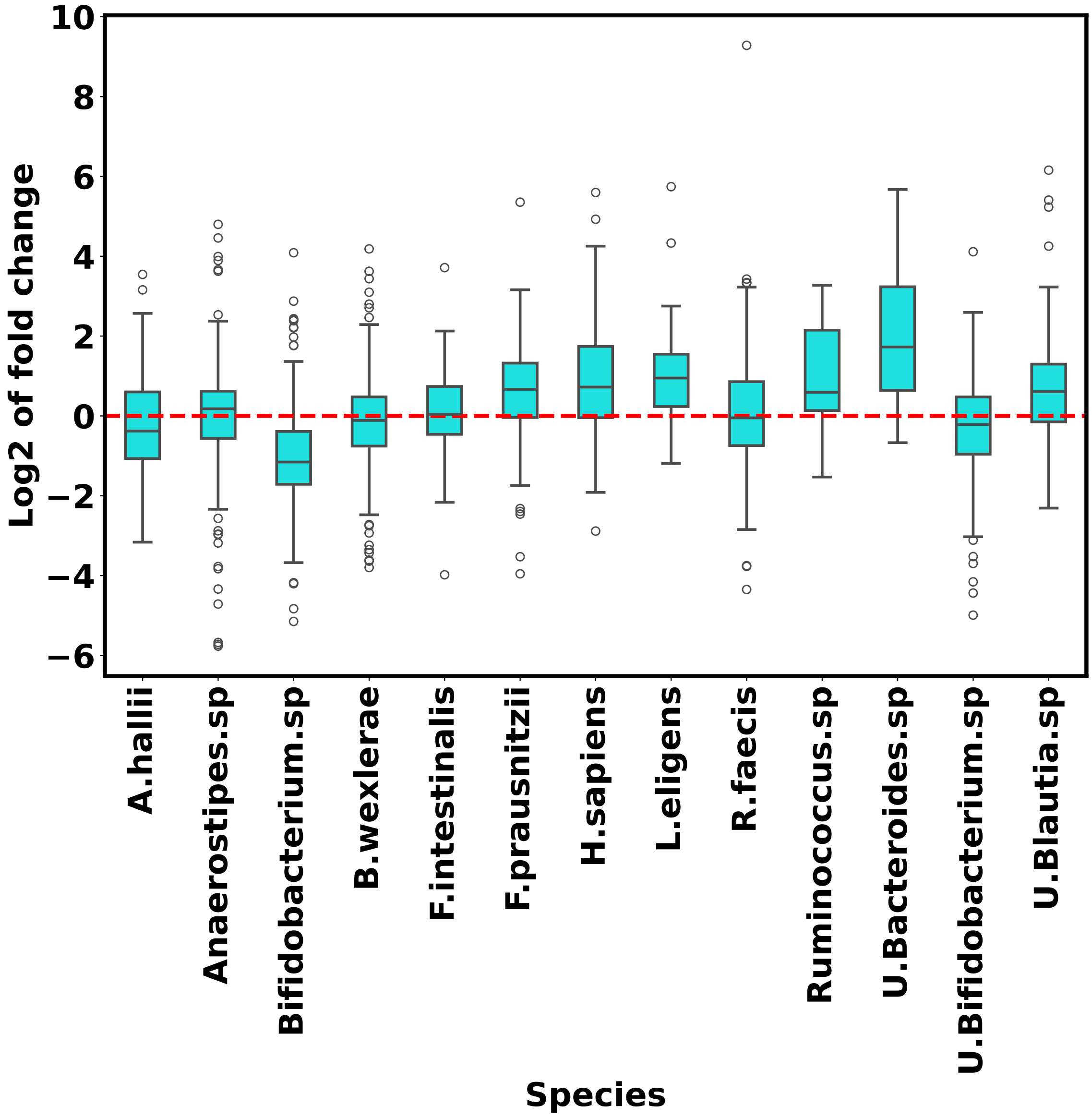

Figure S47: Distributions of log fold change between HM609 and HM604 for each species. The distributions are shown for all species with more than 10 data points (ptoeins). For species that have not been yet named (the ones containing ".sp"), the genus name has been fully spelled. The "U." at the beginning of some of the names denotes "Uncultured".

Figure S48

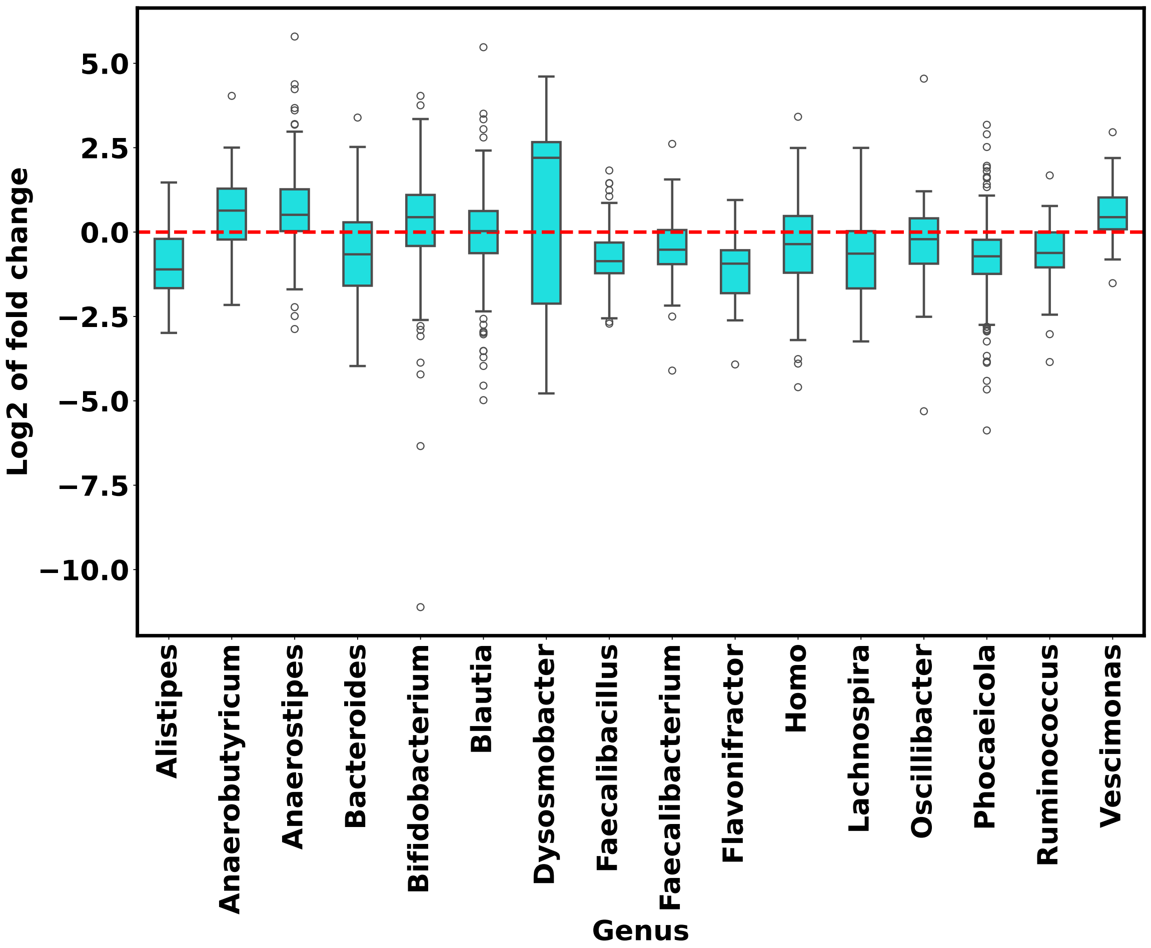

Figure S48: Distributions of log fold change between HM541 and HM604 for each genus.

Figure S49

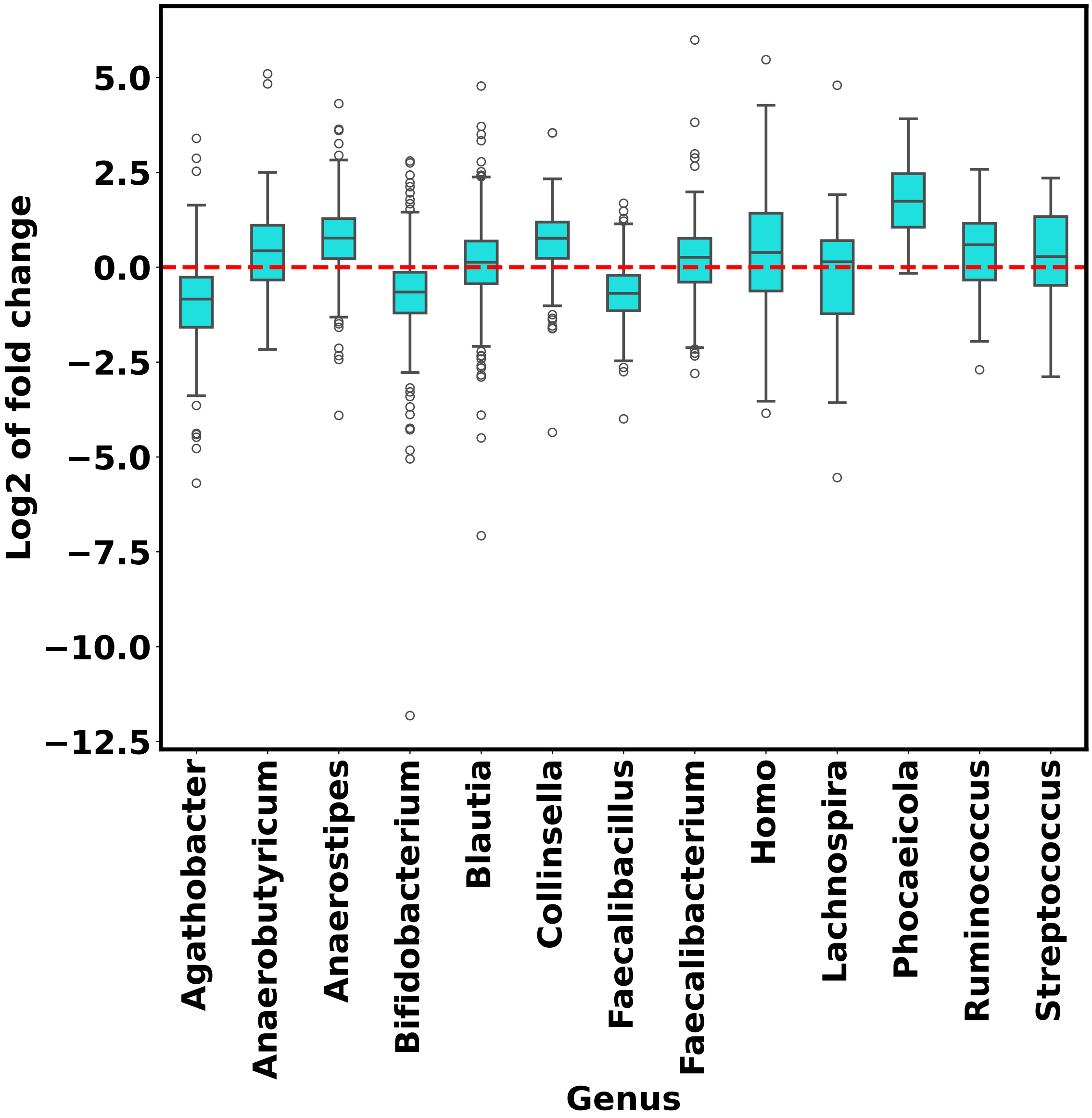

Figure S49: Distributions of log fold change between HM541 and HM609 for each genus.

Figure S50

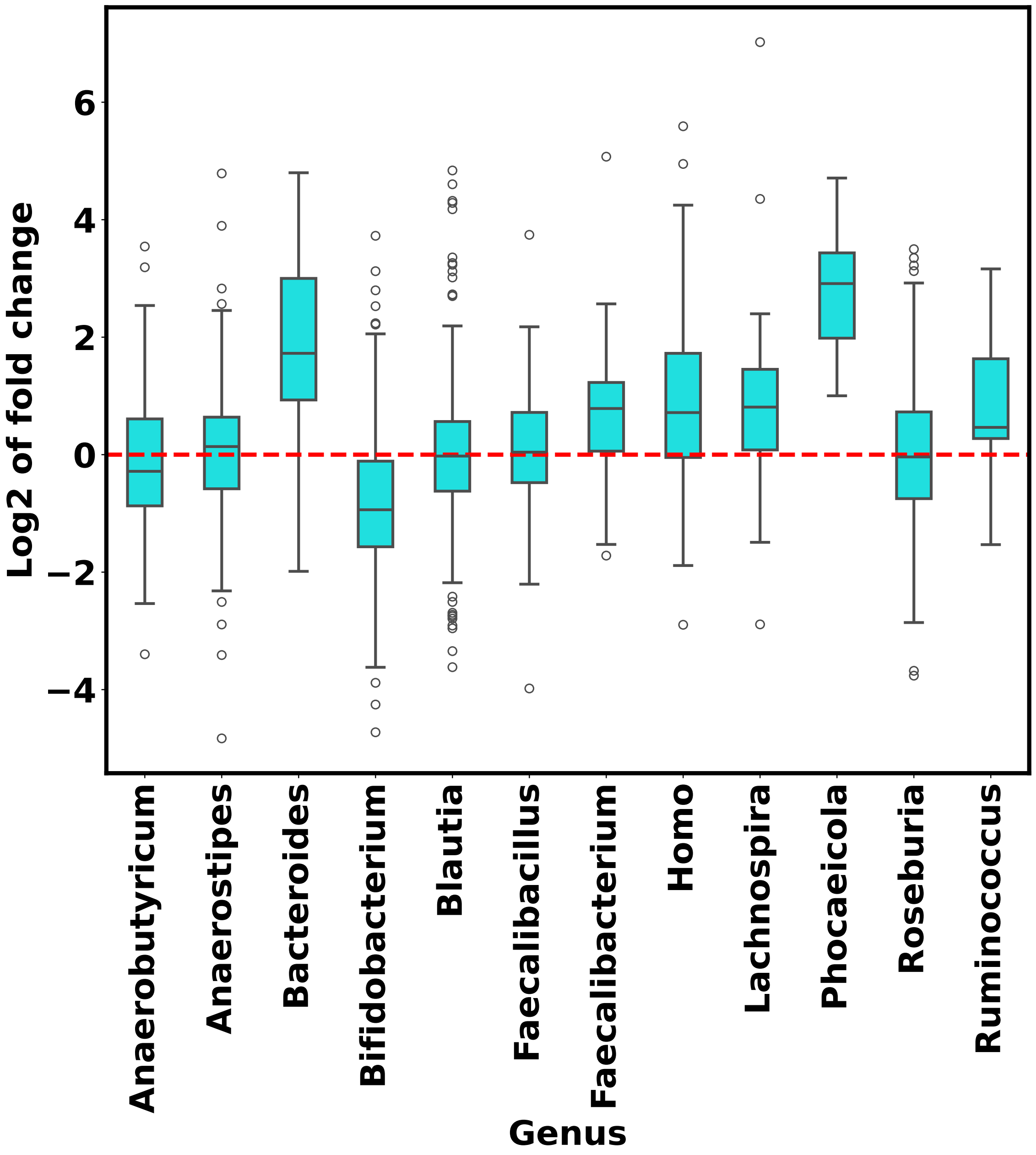

Figure S50: Distributions of log fold change between HM604 and HM609 for each genus.

Figure S51

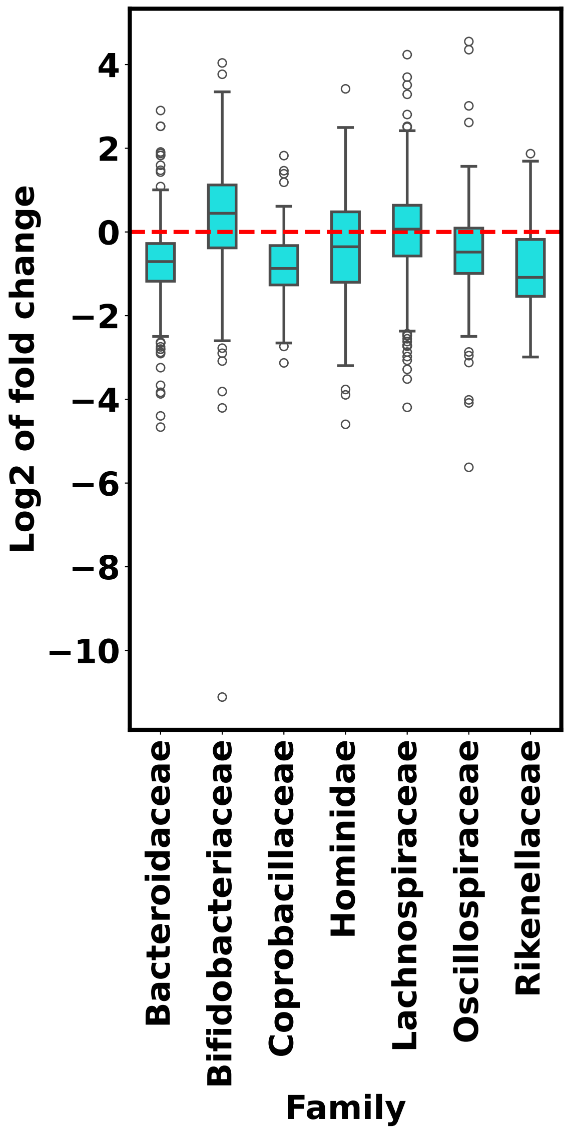

Figure S51: Distributions of log fold change between HM541 and HM604 for each family.

Figure S52

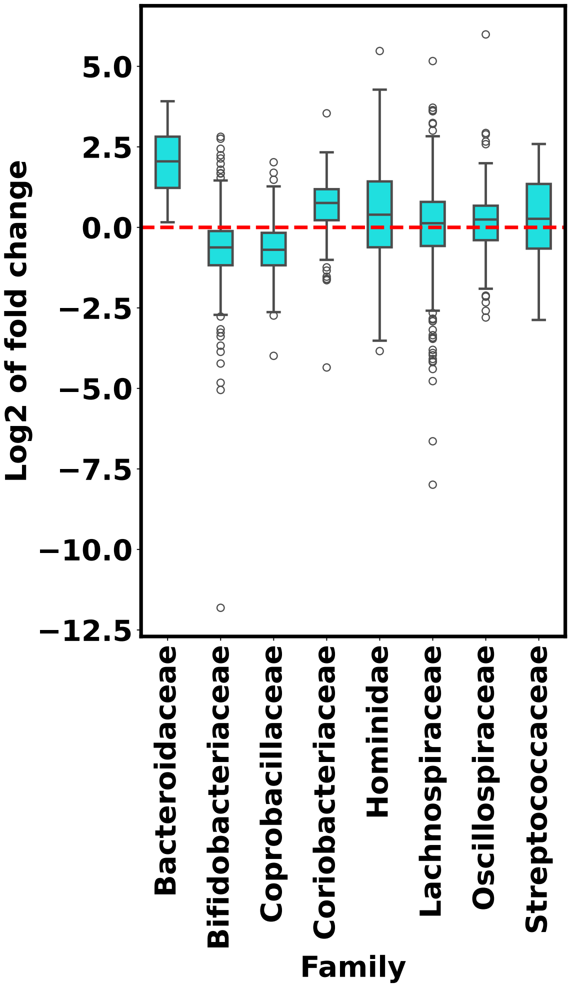

Figure S52: Distributions of log fold change between HM541 and HM609 for each family.

Figure S53

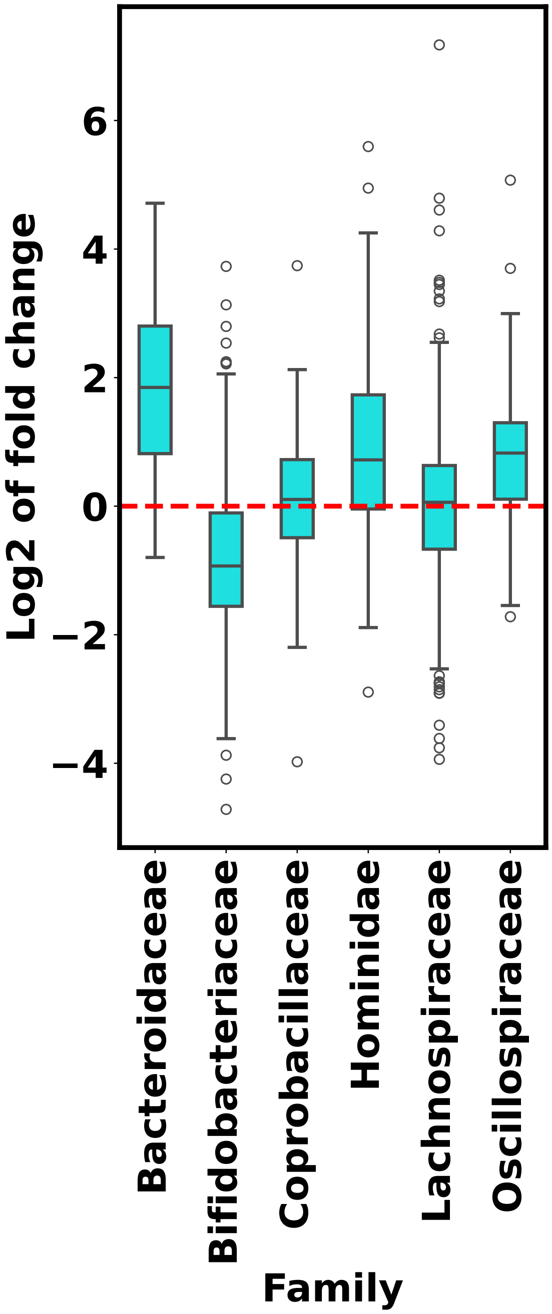

Figure S53: Distributions of log fold change between HM604 and HM609 for each family.

Figure S54

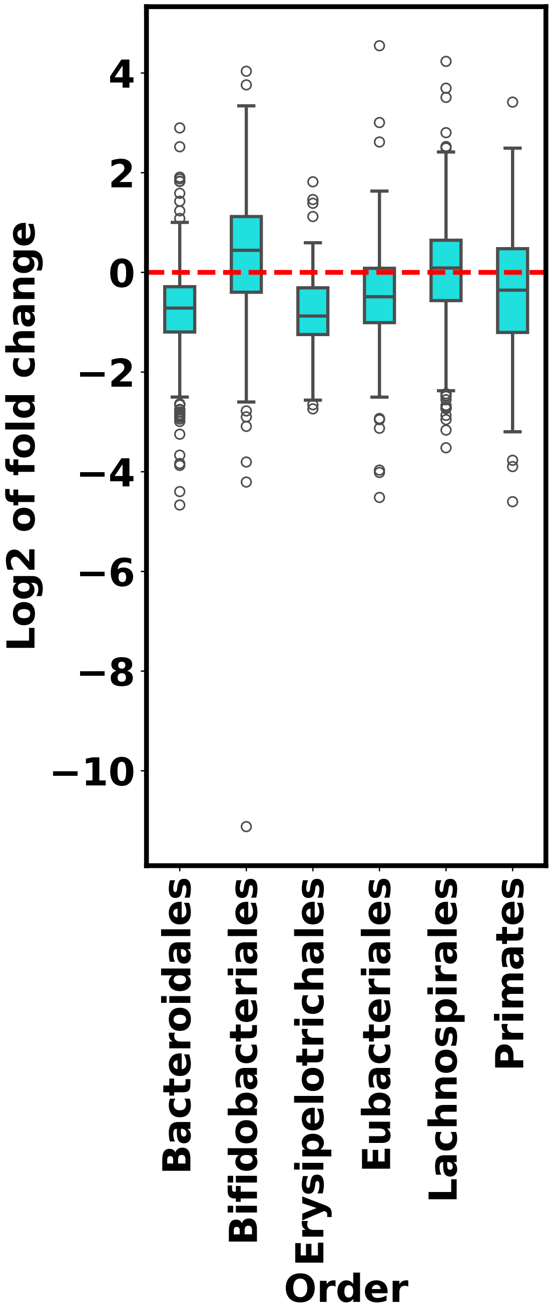

Figure S54: Distributions of log fold change between HM541 and HM604 for each order.

Figure S55

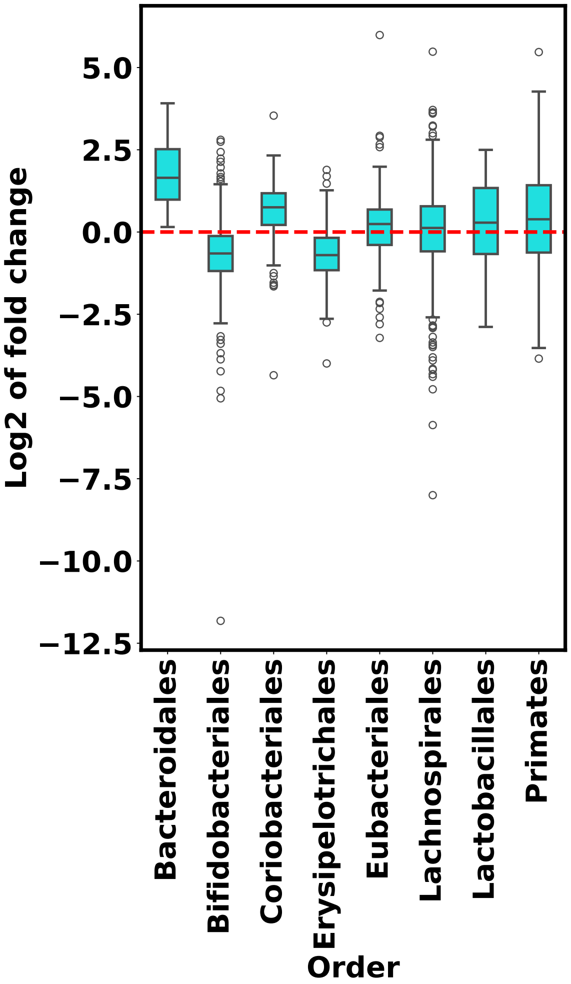

Figure S55: Distributions of log fold change between HM541 and HM609 for each order.

Figure S56

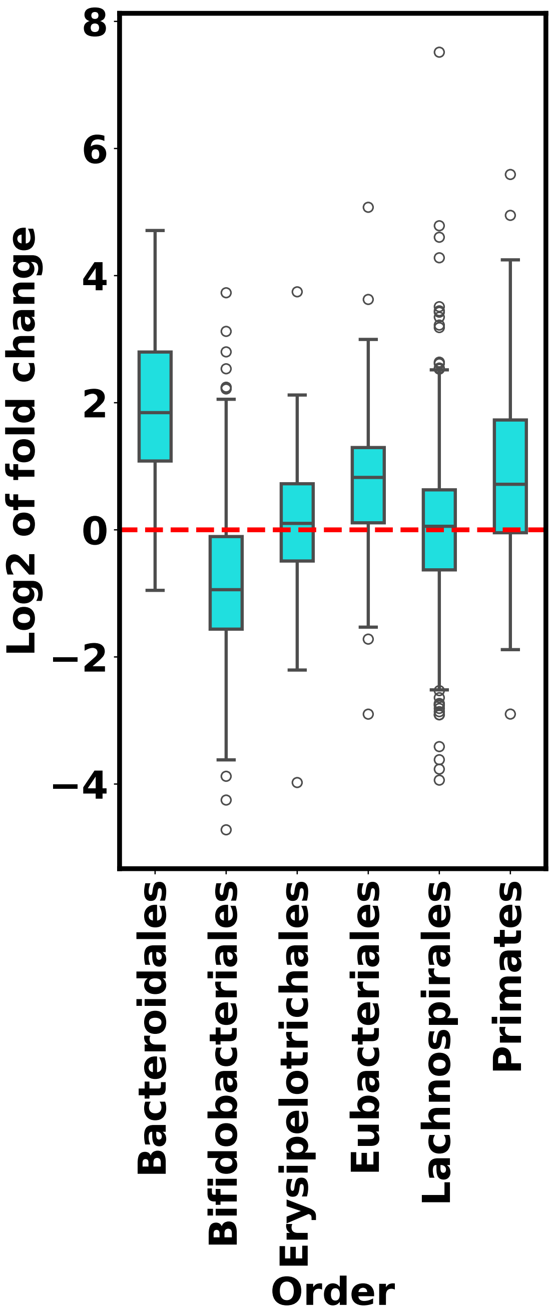

Figure S56: Distributions of log fold change between HM604 and HM609 for each order.

Figure S57

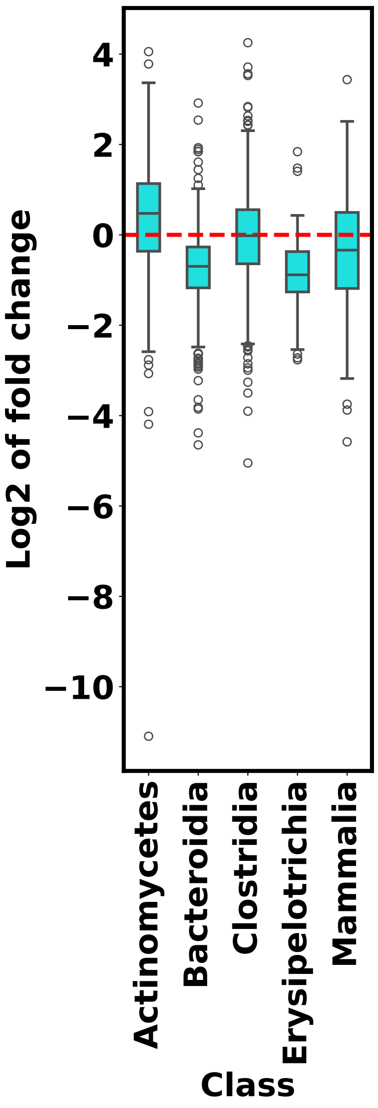

Figure S57: Distributions of log fold change between HM541 and HM604 for each class.

Figure S58

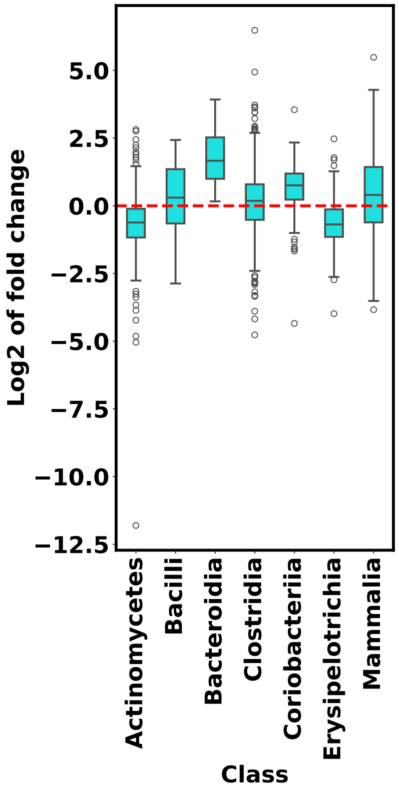

Figure S58: Distributions of log fold change between HM541 and HM609 for each class.

Figure S59

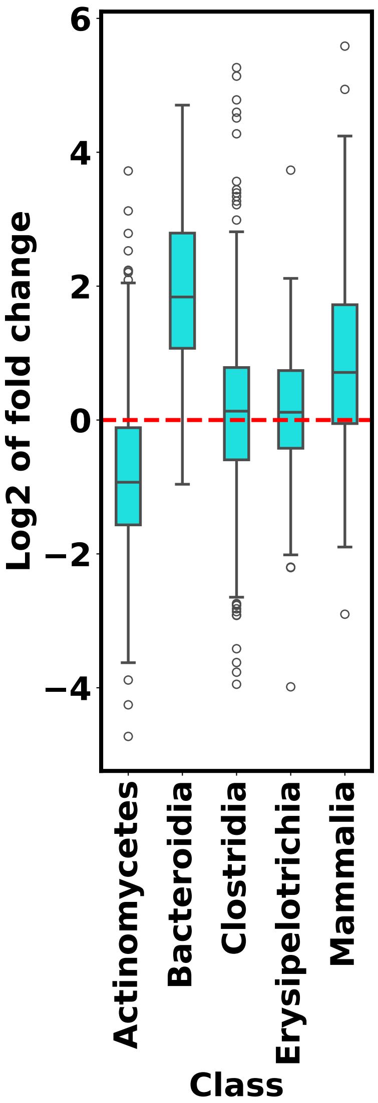

Figure S59: Distributions of log fold change between HM604 and HM609 for each class.

Figure S60

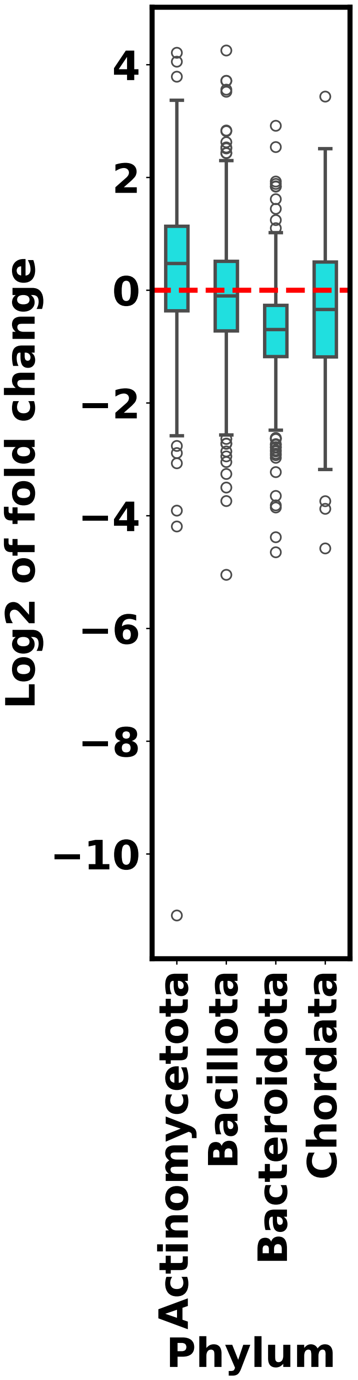

Figure S60: Distributions of log fold change between HM541 and HM604 for each phylum.

Figure S61

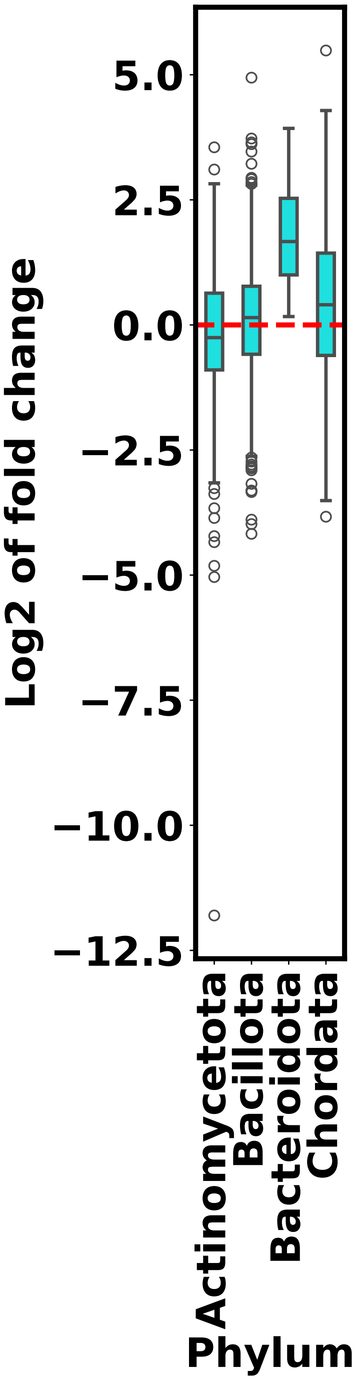

Figure S61: Distributions of log fold change between HM541 and HM609 for each phylum.

Figure S62

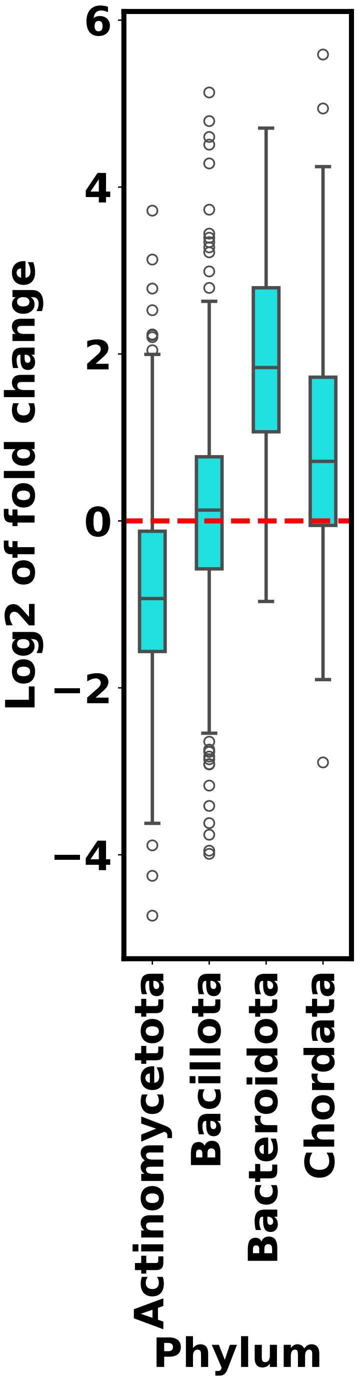

Figure S62: Distributions of log fold change between HM604 and HM609 for each phylum.
